# Lung endothelial cells regulate pulmonary fibrosis through FOXF1/R-Ras signaling

**DOI:** 10.1038/s41467-023-38177-2

**Published:** 2023-05-04

**Authors:** Fenghua Bian, Ying-Wei Lan, Shuyang Zhao, Zicheng Deng, Samriddhi Shukla, Anusha Acharya, Johnny Donovan, Tien Le, David Milewski, Matthew Bacchetta, Ahmed Emad Hozain, Yuliya Tipograf, Ya-Wen Chen, Yan Xu, Donglu Shi, Vladimir V. Kalinichenko, Tanya V. Kalin

**Affiliations:** 1 Division of Pulmonary Biology, the Perinatal Institute of Cincinnati Children’s Research Foundation, Cincinnati, OH USA; 2 Center for Lung Regenerative Medicine, Perinatal Institute, Cincinnati Children’s Hospital Medical Center, Cincinnati, OH USA; 3 The Materials Science and Engineering Program, College of Engineering and Applied Science, University of Cincinnati, Cincinnati, OH USA; 4 Departments of Thoracic and Cardiac Surgery, Department of Biomedical Engineering, Vanderbilt University Medical Center, Nashville, TN USA; 5 Department of Surgery, State University of New York Downstate Medical Center, Brooklyn, NY USA; 6 Department of Cell, Developmental, and Regenerative Biology, Department of Otolaryngology, Institute for Airway Sciences, Black Family Stem Cell Institute, Icahn School of Medicine at Mount Sinai, New York, NY USA; 7 Department of Pediatrics, University of Cincinnati College of Medicine, Cincinnati, OH USA

**Keywords:** Respiratory tract diseases, Transcription

## Abstract

Pulmonary fibrosis results from dysregulated lung repair and involves multiple cell types. The role of endothelial cells (EC) in lung fibrosis is poorly understood. Using single cell RNA-sequencing we identified endothelial transcription factors involved in lung fibrogenesis, including FOXF1, SMAD6, ETV6 and LEF1. Focusing on FOXF1, we found that FOXF1 is decreased in EC within human idiopathic pulmonary fibrosis (IPF) and mouse bleomycin-injured lungs. Endothelial-specific Foxf1 inhibition in mice increased collagen depositions, promoted lung inflammation, and impaired R-Ras signaling. In vitro, FOXF1-deficient EC increased proliferation, invasion and activation of human lung fibroblasts, and stimulated macrophage migration by secreting IL-6, TNFα, CCL2 and CXCL1. FOXF1 inhibited TNFα and CCL2 through direct transcriptional activation of Rras gene promoter. Transgenic overexpression or endothelial-specific nanoparticle delivery of Foxf1 cDNA decreased pulmonary fibrosis in bleomycin-injured mice. Nanoparticle delivery of FOXF1 cDNA can be considered for future therapies in IPF.

## Abstract (web summary)

Pulmonary fibrosis results from dysregulated lung repair, but the role of endothelial cells (EC) in fibrosis is unclear. Here, the authors show that FOXF1/R-Ras signalling in EC inhibits profibrotic mediators and that ECspecific nanoparticle FOXF1 gene therapy decreases lung fibrosis in mice.

## Introduction

Existing anti-fibrotic treatments for pulmonary fibrosis have not significantly improved survival. There is a critical need for new therapeutic approaches. Interstitial lung diseases and pulmonary fibrosis are characterized by injury to the lung parenchyma with varying patterns of inflammation and fibrotic remodeling^1,2^. The etiology and the pathogenesis of lung fibrosis are not completely understood. It has been shown that environmental, age-related, and genetic factors create an alveolar epithelium that is susceptible to injury from either endogenous or exogenous factors^3,4^. It is currently believed that recurrent injury of pulmonary epithelium initiates the pathology, followed by a mild inflammatory response and dysregulated repair process^5^. Dysregulated lung repair results in accumulation of unrestrained myofibroblasts and excessive matrix deposition leading to scarring of pulmonary parenchyma and decline of lung functions. While most studies in pulmonary fibrosis are focused on myofibroblasts, epithelial and inflammatory cells, very few studies examine the role of endothelial cells in pathogenesis of lung fibrosis. The vascular changes in human pulmonary fibrosis got much more attention after recent publications that described a decline in lung capillary cells, and the increased presence of peribronchial endothelial cells in the lung parenchyma^6,7^.

There is a general agreement that alterations in microvessels and vascular remodeling are involved in pulmonary fibrosis. Vascular integrity and endothelial repair are dysregulated, leading to increased vessel permeability, partial loss of capillaries, focal increase in angiogenesis and increased presence of peribronchial endothelial cells in the lung parenchyma^6–8^. The presence of fibrotic stimuli induces the re-programming of normal lung endothelial cells (EC) into fibrosis-associated EC. While normal EC are quiescent and provide tight endothelial barrier, fibrosis-associated EC have high permeability, form leaky endothelial barrier causing focal edema and contributing to fibrosis-associated epithelial injury and inflammation. Perfusion through fibrosis-associated pulmonary vessels is decreased, leading to increased hypoxia within fibrotic lesions and further exacerbating epithelial injury and inflammation^9^.

In addition to barrier functions, tissue-specific endothelium establishes specialized vascular niches that provide angiocrine growth factors necessary to maintain tissue homeostasis as well as guide organ repair and regeneration^10^. In the lungs, the alveolar epithelial cells and their progenitors reside in the vicinity of pulmonary capillary endothelial cells. Angiocrine factors provided by lung endothelial cells include secreted and membrane-bound growth factors, chemokines, cytokines, extracellular matrix components, exosomes that regulate homeostatic and regenerative processes in a paracrine manner^10^. While the changes in angiocrine factors after lung injury have been well documented, the transcriptional regulators driving these changes are not well established. There is a critical need to determine the mechanisms used by lung endothelium to maintain physiological levels of angiocrine factors and to support the normal integrity of endothelial barrier. Normalization of lung vasculature alleviates hypoxia and increases efficacy of drug delivery^11^. Vascular normalization also decreases endothelial permeability and improves the perfusion of blood vessels^11^. Restoring normal microvascular integrity and physiological levels of angiocrine factors will provide additional therapeutic approaches for treatment of pulmonary fibrosis and to promote organ repair without fibrotic scarring.

We sought to uncover the molecular mechanisms critical for vascular normalization and to determine the transcriptional regulators that control the transition of normal EC into fibrosis-associated EC. We used transcriptional profiling of endothelial cells from human and mouse fibrotic lungs and identified FOXF1 as a critical transcriptional regulator of transition from normal to fibrosis-associated EC. FOXF1 is a member of the Forkhead Box (FOX) family of transcription factors. *Foxf1*^−/−^ mice are embryonic lethal^12^. Deletions or “loss-of-function” point mutations in *FOXF1* gene locus were found in most patients with Alveolar Capillary Dysplasia with Misalignment of Pulmonary Veins (ACDMPV)^13,14^, a severe congenital disorder which causes mortality during the first weeks of life. FOXF1 is required for oncogenic properties of PAX3-FOXO1 in rhabdomyosarcoma^15^. In mice, FoxF1 deficiency in EC decreases fetal lung angiogenesis due to inability of FOXF1-deficient ECs to respond to VEGF/FLK1 and BMP9/ACVRL1 signaling^16,17^. While FOXF1 induces angiogenesis during embryogenesis, FOXF1 is also expressed in endothelial cells of adult lungs, where angiogenesis is inactive^18^. Deletion of both Foxf1 alleles in the adult lung disrupts EC adherence junctions and caused vascular leakage^18^. The role of FOXF1 in lung endothelial cells during pulmonary fibrosis is unknown.

In the present study, we used single cell RNA-sequencing and mouse genetics to demonstrate that EC regulate pulmonary fibrosis through FOXF1/R-Ras-dependent inhibition of profibrotic mediators. Our results support the use of FOXF1-activating therapies for vascular normalization in pulmonary fibrosis.

## Results

### Identification of transcription factors that regulate re-programming of normal EC into fibrosis-associated EC in IPF lungs

**Fig. 1:**
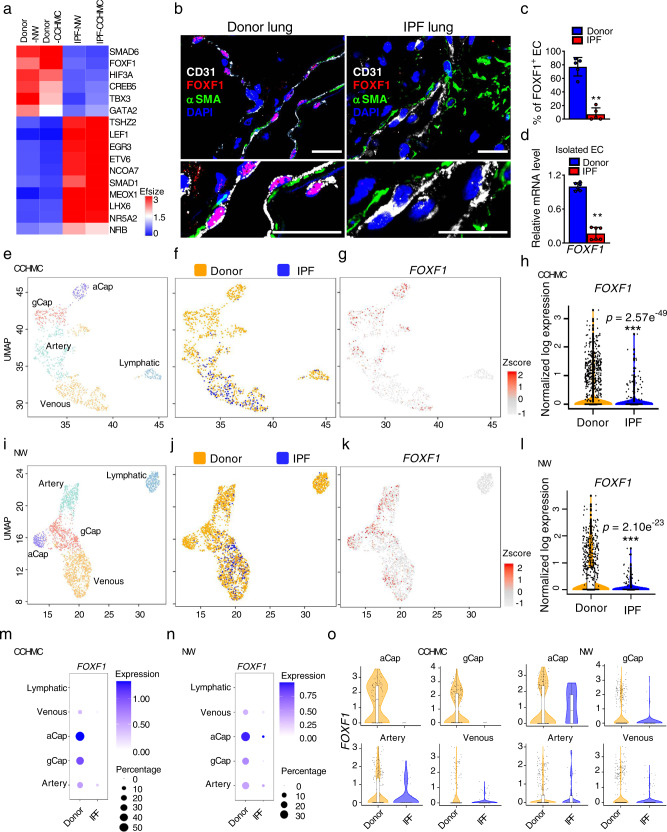
FOXF1 is decreased in ECs of human IPF lung. **a** Heat map shows top differentially expressed transcription factors in EC of IPF lungs compared to EC of donor lungs. scRNA-seq was performed using 3 donor (Donor-CCHMC) and 2 IPF (IPF-CCHMC) lungs. scRNA-seq datasets were also downloaded from GSE 122960 (Donor-NW, *n* = 8 lungs; IPF-NW, *n* = 4 lungs). **b** Co-localization for FOXF1, CD31 and αSMA show decreased FOXF1 in human EC within IPF fibrotic foci (*n* = 5 lungs per group). DAPI (blue) was used to stain cell nuclei. Bar = 20 μm. **c** Decreased percent of FOXF1-positive ECs in IPF lungs. Percentage of FOXF1+/CD31+ double positive ECs were counted in 5 random fields and presented as mean ± SD (*n* = 5 lungs per group), **p = 0.0079, Mann–Whitney Two-tailed test. **d** Decreased *FOXF1* mRNA in EC isolated from human IPF lungs compared to donors (*n* = 6 per group) is shown by qRT-PCR. *ACTB* mRNA was used for normalization, **p = 0.0022, Mann–Whitney Two-tailed test. **e**, **f** Unsupervised UMAP clustering of lung EC from scRNA-seq CCHMC datasets. **g** UMAP plots show *FOXF1* expression in IPF and donor EC clusters after Z-score normalization. **h** Violin plots show decreased expression of *FOXF1* mRNA in ECs from IPF lungs compared to donor lungs (CCHMC datasets). **i**, **j** Unsupervised clustering of lung EC is shown using NW scRNA-seq datasets (GSE 122960). IPF samples (*n* = 4) were compared with donor samples (*n* = 8). **k** Expression of *FOXF1* in IPF and control EC clusters after Z-score normalization. **l** Violin plots show decreased expression of *FOXF1* mRNA in ECs from IPF lungs (NW scRNA-seq datasets). Dot-plots show the decreased *FOXF1* mRNA in venous, aCap, gCap, and arterial clusters of IPF endothelial cells using CCHMC (**m**) and NW (**n**) scRNA-seq datasets. Size of the dots represent frequency of *FOXF1*+ cells in each cluster. Color of the dots represent the expression levels of *FOXF1* in each cluster. *FOXF1* expression is log normalized. **o** Violin plots show decreased *FOXF1* mRNA in aCap, gCap, arterial and venous endothelial cells of IPF lungs compared to donor lungs in both CCHMC and NW data sets. *FOXF1* expression is log normalized. Source data are provided as a Source Data file.

To identify transcriptional regulators that control re-programming of normal EC into fibrosis-associated EC, we performed single cell RNA-sequencing (10X genomics) to compare the transcriptome of endothelial cells in donor and idiopathic pulmonary fibrosis (IPF) lungs (Cincinnati Children’s Hospital Medical Center (CCHMC) datasets, Supplementary Fig. S1a, b). We also downloaded and analyzed the published scRNA-seq data^19^ from donor and IPF lungs (Northwestern University (NW) datasets, Supplementary Fig. S1c, d). Endothelial cell in both datasets were identified as cells expressing *Pecam1* (CD31)*, Cdh5* (VE cadherin) and lacking *Ptprc* (CD45) mRNAs, consistent with published studies^20^. Cross-comparison of two scRNA-seq datasets identified several top differentially expressed endothelial transcription factors. Among universally downregulated transcription factors in IPF endothelial cells were FOXF1, SMAD6, HIF3A, CREB5, TBX3 and GATA2 (Fig. 1a). Universally upregulated transcription factors included ETV6, LEF1, EGR3, MEOX1 and SMAD1 (Fig. 1a). For the follow-up studies, we decided to focus on FOXF1 transcription factor because it’s expression is highly enriched in lung endothelial cells compared to endothelial cells of other organs^18,21^ and because the FOXF1 loss-of-function gene mutations are linked to ACDMPV, a fatal congenital lung disease with significant fibrotic remodeling^13^. To verify that identified decrease in *FOXF1* is not limited to two datasets, we have also analyzed the publicly available datasets from Yale University and Vanderbilt University^6,7^. We have confirmed the decreased levels of *FOXF1* mRNA in endothelial cells of patients with IPF compared to donor controls (Supplementary Fig. S2a). We sought to determine whether downregulation of FOXF1 in fibrosis-associated EC is essential for lung fibrogenesis.

### Expression of FOXF1 is decreased in endothelial cells within fibrotic lesions of human IPF lungs

Using immunostaining with FOXF1, CD31 and αSMA antibodies, we found that FOXF1 protein was undetectable in EC within fibrotic lesions of IPF lungs identified as αSMA-positive regions (Fig. 1b and Supplementary Fig. S2b). While in human donor lungs, FOXF1 protein was detected in approximately 80% of endothelial cells, only 5-10% of endothelial cells expressed FOXF1 in IPF lungs (Fig. 1c). We further verified by qRT-PCR that *FOXF1 mRNA* was decreased in FACS-sorted EC from IPF lungs compared to FACS-sorted EC from donor lungs (Fig. 1d). Thus, both mRNA and protein levels of FOXF1 are decreased in endothelial cells of IPF lungs.

To identify FOXF1-expressing lung endothelial cells in scRNA-seq CCHMC dataset, endothelial cells from donor and IPF lungs were visualized using uniform manifold approximation and projection (UMAP) after samples integration with Harmony^22^ (Fig. 1e–g). Both *FOXF1* mRNA levels and the total number of FOXF1-expressing EC were decreased in IPF lungs compared to donor lungs (Fig. 1h). To demonstrate that these results are not limited to CCHMC scRNA-seq dataset, we analyzed the publicly available NW scRNA-seq dataset containing endothelial cells from IPF and donor lungs (GSE 122960^19^). Consistent with the results from CCHMC dataset, both the percentage of FOXF1-positive endothelial cells and *FOXF1* mRNA levels were decreased in IPF lungs from NW dataset (Fig. 1i–l).

Based on gene expression signatures, pulmonary endothelial cells from both CCHMC and NW datasets were subdivided into five sub-clusters: arterial, general capillary (gCAP), alveolar capillary (aCAP), venous and lymphatic ECs (Fig. 1e, i, Supplementary Fig. S1a–d). Genes known to be selectively expressed in different sub-types of endothelial cells were used to annotate these sub-clusters^23,24^. The arterial EC sub-cluster was identified as cells expressing *EFNB2, GJA5, DKK2 and HEY1*, whereas the venous EC sub-cluster included cells expressing *ACKR1*, CLU and VWF (Supplementary Fig. S1a, b). The gCAP sub-cluster was enriched in GPIHBP1 and SLC6A4 transcripts, aCAP was enriched in EDNRB, APLN and HPGD, and lymphatic EC sub-cluster expressed *PROX1, CCL21* and *PDPN* (Supplementary Fig. S1a–d). *FOXF1* was detected in arterial, venous, and capillary sub-clusters, with the highest expression of *FOXF1* in aCAPs and gCAPs (Fig. 1m, n). *FOXF1* was undetectable in lymphatic EC (Fig. 1m, n). Also, *FOXF1* was not expressed in the COL15+ endothelial cells that were identified in the IPF lungs, but not in the control donor lungs (Supplementary Fig. S2c), confirming the published data^6^. In both scRNA-seq datasets, *FOXF1* expression was decreased in capillary, arterial and venous sub-clusters of IPF lungs compared to donor lungs (Fig. 1m–o). Consistent with scRNA-seq data, RNA in situ hybridization showed that both the percentage of FOXF1-positive EC and expression levels of FOXF1 transcripts were decreased in capillary, arterial and venous sub-clusters of IPF lungs compared to donor lungs (Supplementary Fig. S3).

### FOXF1 is decreased in endothelial cells within fibrotic lesions of mouse bleomycin-injured lungs

**Fig. 2:**
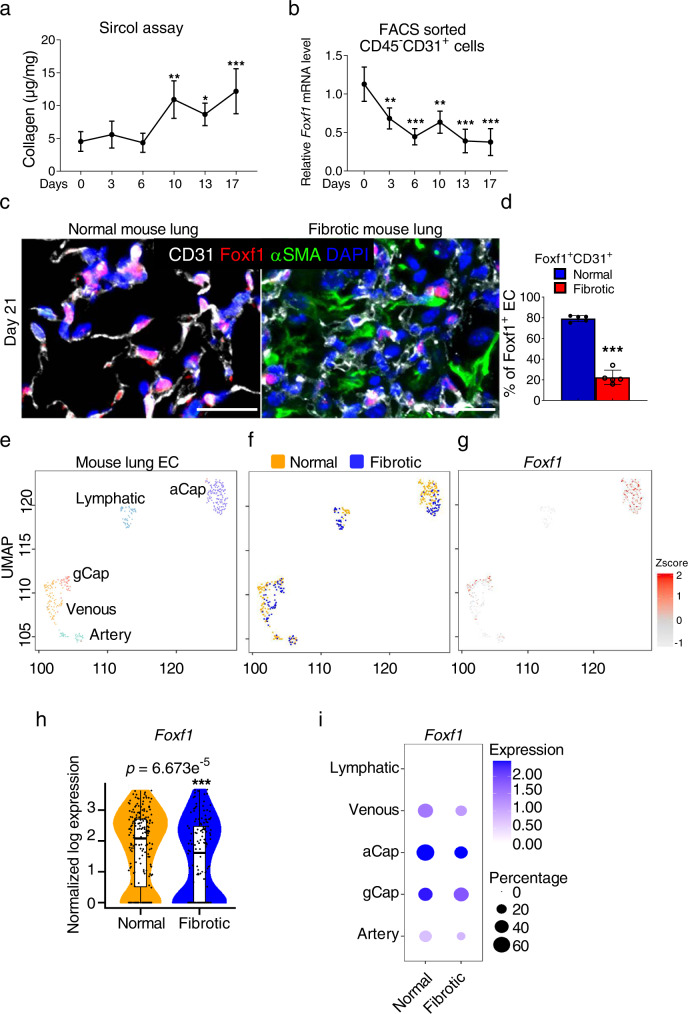
Expression of FOXF1 is decreased in endothelial cells within fibrotic lesions of mouse lungs. **a** Time-dependent accumulation of collagen in murine lungs after chronic bleomycin injury is quantified using Sircol collagen assay. Wild type mice were treated with three weekly IT injections of bleomycin to induce lung fibrosis (Day0, 3, 6, 10, 17, *n* = 4 mice per group; Day 13, *n* = 6 mice). **b** Time-dependent decrease of *Foxf1 mRNA* is shown in FACS-sorted lung endothelial cells during lung fibrogenesis using qRT-PCR (Day 0, 6,10, *n* = 3; Day 3, *n* = 4; Day13 *n* = 7; Day 17, *n* = 8, mice per group). **c** Co-localization studies show decreased FOXF1 in endothelial cells and decreased number of FOXF1^+^ endothelial cells within lung fibrotic foci at day 21 after bleomycin treatment. Mouse normal lungs (*n* = 5) and bleomycin-treated lungs (*n* = 5) were stained with antibodies against FOXF1 (red), CD31 (white) and αSMA (green). DAPI (blue) was used to visualize the nuclei. Bar = 25 µm. **d** Percent of FOXF1^+^/CD31^+^ double positive cells among CD31^+^ cells were counted in 5 random fields and presented as mean ± SD (*n* = 5 mice per group). ***p < 0.001. The integrated projection of lung EC from normal and bleomycin-treated fibrotic lungs (**e**, colored by cell type. **f**, yellow- normal lungs; blue- fibrotic lungs). Single cell RNA-seq was performed using pooled normal controls (*n* = 4) and bleomycin-treated (*n* = 6) mouse lungs. **g** UMAP plots show *Foxf1* expression in endothelial cells of normal and fibrotic mouse lungs after Z-score normalization. **h** Violin plots show decreased expression of *Foxf1* mRNA in ECs from fibrotic lungs. **i** Both *Foxf1* mRNA expression and the frequency of *Foxf1*-positive cells are decreased in venous, aCap, gCap, and arterial endothelial cells from fibrotic lungs. *Foxf1* is not expressed in lymphatic endothelial cells. *Foxf1* expression is log normalized. Data presented as mean ± SD. *p < 0.05, **p < 0.01, ***p < 0.001. For two group comparisons, T-test (two-tailed) analyses were performed. For more than two group, statistical significance was determined by one-way ANOVA followed by Dunnett’s test. Source data are provided as a Source Data file.

We next examined expression of FOXF1 in endothelial cells in a mouse model of pulmonary fibrosis. Three weekly intratracheal (IT) injections of bleomycin were used to induce lung fibrosis in wild type mice (Supplementary Fig. S4a). Bleomycin administration caused a time-dependent increase in collagen depositions as quantified by Sircol assay (Fig. 2a) and confirmed by H&E and Sirius red/Fast green staining (Supplementary Fig. S4b). Increased accumulation of inflammatory cells in bleomycin-treated lungs was shown using flow cytometry analysis for CD45^+^ cells (Supplementary Fig. S4c). Next, we FACS-sorted CD31^+^/CD45^−^ lung endothelial cells at different time points after bleomycin treatment and measured endothelial *Foxf1* expression by qRT-PCR. A decrease in endothelial *Foxf1* mRNA was observed as early as day 3 after the first bleomycin treatment (Fig. 2b). The percentage of FOXF1-positive ECs in lung tissue was also decreased as early as day 3 after the first bleomycin treatment as shown by co-localization of FOXF1 with endothelial-specific ERG transcription factor (Supplementary Fig. S4d). Immunostaining for FOXF1 and CD31 showed decreased FOXF1 protein expression in endothelial cells within fibrotic lesions identified as αSMA-positive areas (Fig. 2d, right panel). Consistent with human IPF lungs (Fig. 1b, c), the number of FOXF1^+^/CD31^+^ endothelial cells was decreased in murine fibrotic lungs (Fig. 2c).

Next, we performed a single cell RNA-sequencing of bleomycin-treated and control mouse lungs. *Foxf1*-expressing endothelial cells were visualized using UMAP (Fig. 2e–g). Consistent with human IPF lungs, bleomycin-treated mouse lungs had reduced numbers of *Foxf1*-positive endothelial cells and decreased expression of *Foxf1* mRNA in these cells (Fig. 2h). Mouse lung endothelial cells were further subdivided into arterial, venous, aCAP, gCAP and lymphatic sub-clusters (Supplementary Fig. S5 and Fig. 2e). Endothelial cells expressing *Foxf1* transcript were present in arterial, venous, aCAP and gCAP sub-clusters, but were completely absent in lymphatic cells (Fig. 2i). RNA in situ hybridization showed that both the percent of FOXF1-positive endothelial cells as well as the expression levels of FOXF1 transcripts were decreased in arterial, venous, aCAP and gCAP sub-clusters of bleomycin-treated lungs compared to control lungs (Supplementary Fig. S6). Altogether, FOXF1 expression is decreased in most EC types in human and mouse fibrotic lungs.

### Deletion of FOXF1 in endothelial cells accelerates pulmonary fibrosis

To determine the role of FOXF1 in endothelial cells during pulmonary fibrosis, we used mice in which the *Foxf1* gene was specifically deleted in endothelial cells using *Pdgfb-CreER* transgene, which does not target other cell types in the adult lung^18,25^. Since homozygous (*Pdgfb-CreER* /*Foxf1*^*fl/fl*^ mice or *endFoxf1*^*−/−*^) mice developed lung edema and respiratory insufficiency^18^, we used heterozygous *endFoxf1*^*+/−*^ mice for lung fibrosis studies (Supplementary Fig. S7a). Naïve heterozygous Tam-treated *endFoxf1*^*+/−*^ mice were phenotypically normal, had normal lung histology and alveolar capillary density as shown by CD31 staining (Supplementary Fig. S7b and^18^). Since the Pdgfb-Cre transgene contain GFP^18^, we used flow cytometry to demonstrate that the transgene targets only CD45^−^CD31^+^ (endothelial), but CD45^+^CD31^−^ (hematopoietic) or CD45^−^CD31^−^ (non-endothelial, non-hematopoietic) cell types in the lung (Supplementary Fig. S7c, d). The scRNA-seq analysis showed co-expression of endothelial *Foxf1* and *Pdgfb* mRNA transcripts in the lung EC (Supplementary Fig. S7f).

**Fig. 3:**
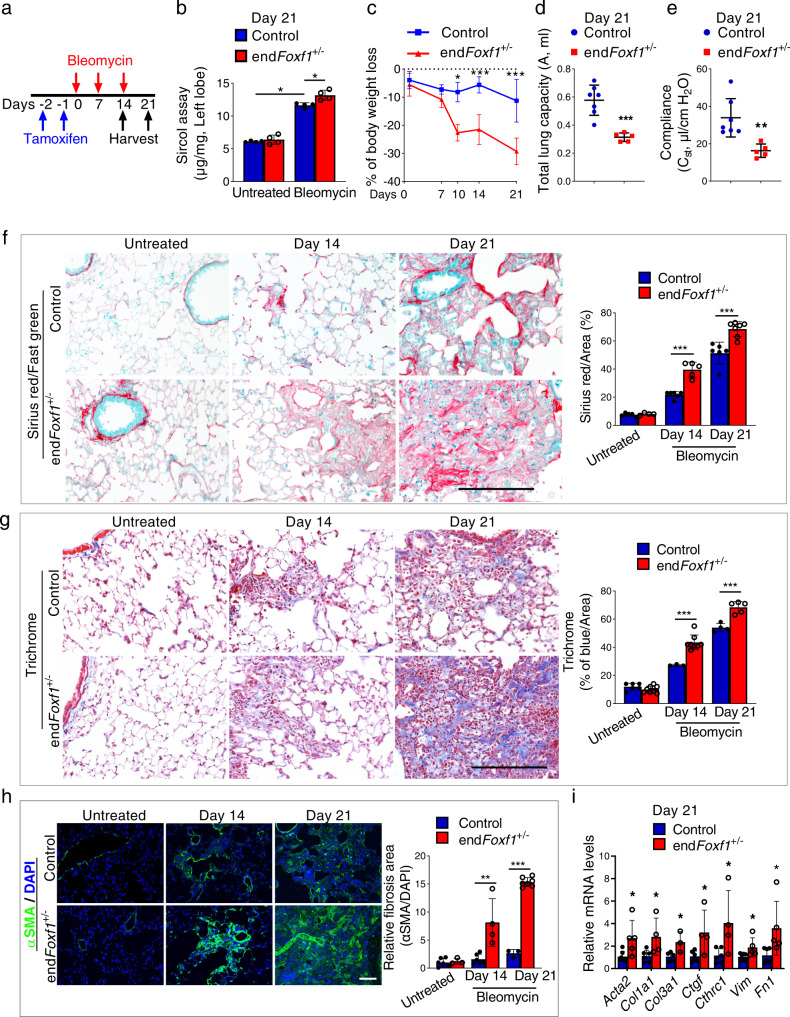
Deletion of FOXF1 in endothelial cells accelerates pulmonary fibrosis. **a** Schematic diagram of bleomycin administration to induce lung fibrosis and tamoxifen (TAM) administration to delete *Foxf1* in end*Foxf1*^+/−^ and control *Foxf1*^+/−^ mice. **b** Lung collagen was quantified at 21 days after bleomycin administration by Sircol assay (*n* = 4 mice per group). Data presented as mean ± SD. *p < 0.05. Mann–Whitney Two-tailed test were performed. **c** Increased body weight loss in end*Foxf1*^+/−^ mice is shown at different time points compared to control mice. *n* = 6 per group. **d**, **e** Decreased lung capacity and lung compliance in end*Foxf1*^+/−^ (*n* = 5) mice at day 21 after bleomycin administration shown using FlexiVent. Control, *n* = 7. ***p = 0.0004 (**d**). **p = 0.0031 (**e**). **f**–**h** Increased severity of lung fibrosis in end*Foxf1*^+/−^ mice is shown using (**f**) Sirius red/Fast green at Days 14 and 21 after bleomycin treatment (untreated control *n* = 5 and end*Foxf1*^+/−^ *n* = 4; bleomycin treated Day 14 control *n* = 6 and end*Foxf1*^+/−^ *n* = 5; Day 21 control *n* = 6 and end*Foxf1*^+/−^ *n* = 7) and (**g**) Trichrome staining (untreated control *n* = 6 and end*Foxf1*^+/−^ *n* = 8; Day 14 control *n* = 3 and end*Foxf1*^+/−^ *n* = 8; Day 21 control *n* = 4 and end*Foxf1*^+/−^ *n* = 5), as well as immunostaining for αSMA (green) (untreated control, *n* = 6; untreated end*Foxf1*^+/−^ *n* = 3; Day 14 control, *n* = 6; Day 14 end*Foxf1*^+/−^, *n* = 4; Day 21 control, *n* = 3; Day 21 end*Foxf1*^+/−^, *n* = 7) (**h**)^.^ Bar = 100 µm. Sirius Red binds to all types of collagens, whereas fast green stains non-collagenous proteins. **i** Endothelial deletion of *Foxf1* increases mRNA levels of collagen genes in total lung RNA as shown by qRT-PCR. Total lung mRNA was extracted from control and bleomycin-treated end*Foxf1*^+/−^mice on day 21. *Actb* mRNA was used for normalization. Control *Acta2*, *n* = 7; Control *Col1a1*, *n* = 6; Control *Col3a*, *n* = 5; Control *Ctgf*, *n* = 6; Control *Cthrc1*, *n* = 6; Control *Vim*, *n* = 7; Control *Fn1*, *n* = 6; end*Foxf1*^+/−^ *Acta2*, *n* = 5; end*Foxf1*^+/−^ *Col1a1*, *n* = 4; end*Foxf1*^+/−^ *Col3a*, *n* = 3; end*Foxf1*^+/−^ *Ctgf*, *n* = 4; end*Foxf1*^+/−^ *Cthrc1*, *n* = 4; end*Foxf1*^+/−^ *Vim*, *n* = 5; end*Foxf1*^+/−^ *Fn1*, *n* = 5. Data presented as mean ± SD. *p < 0.05, **p < 0.01, ***p < 0.001. T-test (two-tailed) analyses were performed. Source data are provided as a Source Data file.

Three weekly IT injections of bleomycin were used to induce lung fibrosis in Tam-treated *endFoxf1*^*+/−*^ and control *Foxf1*^*fl/fl*^ mice (Fig. 3a). Using FACS-sorted lung endothelial cells, we have shown that bleomycin treatment caused more profound decrease of *Foxf1* mRNA in *endFoxf1*^*+/−*^ endothelial cells compared to control endothelial cells (Supplementary Fig. S8a). Decreased *Foxf1* in endothelial cells was associated with more severe fibrosis in bleomycin-treated *endFoxf1*^*+/−*^ mice compared to bleomycin-treated control mice, as shown by quantifying collagen depositions in the lung tissue using Sircol assay (Fig. 3b). Compared to controls, bleomycin-treated *endFoxf1*^*+/−*^ mice had increased Ashcroft scores (Supplementary Fig. S8b, c), decreased body weights (Fig. 3c), reduced lung capacity and compliance (Fig. 3d, e), impaired lung mechanics (Supplementary Fig. S8d–j) and arterial oxygenation (Supplementary Fig. S8k). Lung fibrosis in *endFoxf1*^*+/−*^ mice developed faster and was more severe compared to control mice, as shown by Sirius red/fast green (Fig. 3f), Trichrome (Fig. 3g), and immunostaining for αSMA (Fig. 3h). Increased fibrotic depositions in bleomycin-treated *endFoxf1*^*+/−*^ lungs were also supported by increased *Acta2*, *Col1a1, Col3a1, Ctgf, Cthrc1, Vim* and *Fn1* mRNAs (Fig. 3i). Thus, FOXF1 deficiency in murine endothelial cells exacerbates bleomycin-induced pulmonary fibrosis.

### FOXF1-deficient endothelial cells increase myofibroblast activation

**Fig. 4:**
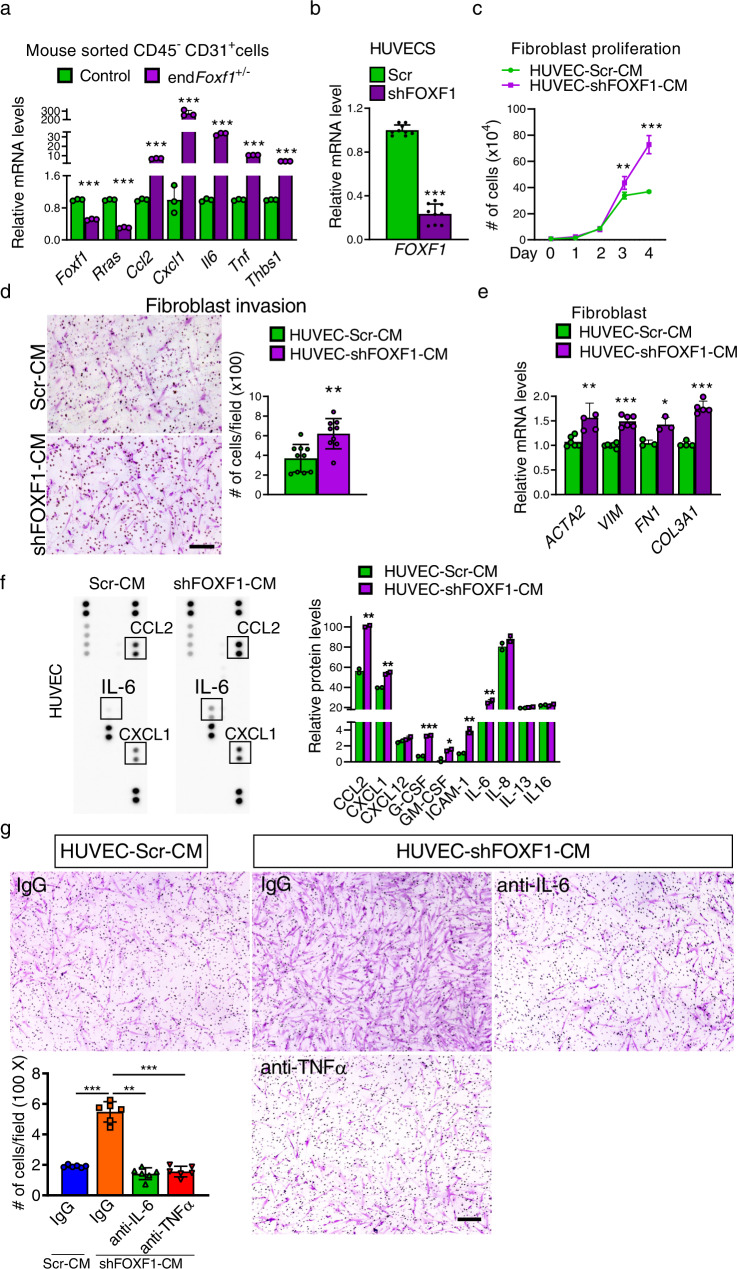
FOXF1-deficient endothelial cells increase myofibroblast activation. **a** qRT-PCR shows increased expression of fibrosis-associated genes in FACS-sorted endothelial cells of end*Foxf1*^+/−^ lungs at day 21 after bleomycin administration. *Actb* mRNA was used for normalization. *n* = 3 mice per group. **b** Efficient inhibition of *FOXF1* expression in sh*FOXF1*-transfected HUVEC cells is shown with qRT-PCR. *ACTB* mRNA was used for normalization. *n* = 9 samples per group. **c** CM from FOXF1-deficient HUVECs increases fibroblast proliferation. CCD-19Lu fibroblasts were cultured in the presence of CM from control or *FOXF1*-deficient HUVECs. *n* = 3 samples per group. Day 3, **p = 00290; Day 4 ***p < 0.0001 by two-way ANOVA test. **d** Conditioned media (CM) from FOXF1-deficient HUVECs increases invasion of cultured CCD-19Lu fibroblasts. Human CCD-19Lu fibroblasts were seeded on the insert of transwell chamber coated with matrigel in the presence of CM from scrambled control (Scr-CM, *n* = 10) or FOXF1-deficient (shFOXF1-CM, *n* = 9) HUVECs. Graph represents average numbers of invaded cells per field. Bar = 200 μm. **p = 0.0019. **e** CCD-19Lu fibroblasts cultured in CM from FOXF1-deficient HUVECs had increased expression of pro-fibrotic genes compared to fibroblasts cultured in CM from control HUVECs as shown by qRT-PCR. HUVEC-Scr-CM *ACTA2*, *n* = 6; HUVEC-Scr-CM *VIM*, *n* = 6; HUVEC-Scr-CM *FN1*, *n* = 3; HUVEC-Scr-CM *COL3A1*, *n* = 4; HUVEC-shFOXF1-CM *ACTA2*, *n* = 5; HUVEC-shFOXF1-CM *VIM*, *n* = 6; HUVEC-shFOXF1-CM *FN1*, *n* = 3; HUVEC-shFOXF1-CM *COL3A1*, *n* = 5. **f** CM from FOXF1-deficient HUVECs had increased levels of pro-inflammatory mediators as determined by Proteome Profiler Human Cytokine Array (*n* = 2). **g** Inhibition of IL-6 and TNFα using blocking antibodies attenuated CCD-19Lu fibroblast invasion in the presence of CM from FOXF1-deficient HUVECs. Bar = 200 μm. *n* = 6 samples per group. Data presented as mean ± SD, *p < 0.05, **p < 0.01, ***p < 0.001. CM conditioned medium. For two group comparisons, T-test (two-tailed) analyses were performed. Source data are provided as a Source Data file.

To identify molecular mechanisms by which FOXF1-deficient ECs promote lung fibrogenesis, we performed bulk RNA-seq using FACS-sorted endothelial cells from bleomycin-treated control and end*Foxf1*^+/−^ lungs (Supplementary Fig. S9). Gene Set Enrichment Analysis (GSEA) of RNA-seq data showed that the most enriched functional categories in end*Foxf1*^+/−^ endothelial cell were wound healing, extracellular matrix organization, cell migration, blood vessel remodeling, inflammation, myeloid leukocyte migration, and RHO GTPase signaling (Supplementary Fig. S9a–d). Consistent with inactivation of one *Foxf1* allele, *Foxf1* mRNA was decreased 2.1-fold in ECs of bleomycin-treated end*Foxf1*^+/−^ lungs compared to control lungs (Supplementary Fig. S9a), a finding confirmed by qRT-PCR of FACS-sorted ECs (Fig. 4a). FOXF1-deficiency in end*Foxf1*^+/−^ ECs was associated with increased expression of pro-fibrotic and pro-inflammatory genes, including *Il6*, *Tnfα*, *Ccl2*, *Cxcl1* and *Thbs1* (Supplementary Fig. S9a), findings validated by qRT-PCR (Fig. 4a).

Next, we determined the effect of FOXF1 deficiency on secretion of profibrotic and proinflammatory mediators by endothelial cells in vitro. Human endothelial cells, HUVEC, were infected with lentiviruses carrying either *FOXF1* shRNA or control shRNA. The shFOXF1-treated HUVECs exhibited decreased *FOXF1* mRNA as shown by qRT-PCR (Fig. 4b), demonstrating approximately similar efficiency of FOXF1 inhibition compared to haploinsufficient endothelial cells from *endFoxf1*^*+/−*^ mice (Fig. 4a). Conditioned media (CM) from control and FOXF1-deficient HUVECs were collected. Invasion and proliferation of human CCD-19Lu lung fibroblasts were measured in the presence of CM in vitro. CM from FOXF1-deficient HUVECs increased invasion and proliferation of lung fibroblasts compared to CM from control cells (Fig. 4c, d). CM from FOXF1-deficient HUVECs also increased expression of *ACT2*, *VIM*, *FN1* and *COL3A1* in human CCD-19Lu fibroblasts (Fig. 4e). To verify that the results are not limited to HUVECs, we also used human pulmonary arterial endothelial cells (HPAEC) and human pulmonary microvascular endothelial cells (HPMEC) to confirm that CM from FOXF1-deficient HPAEC and HPMEC cells increased invasion, proliferation as well as expression of pro-fibrotic genes in lung fibroblasts (Supplementary Figs. S10a–d and S11a–d). Thus, FOXF1-deficient endothelial cells induced the pro-fibrotic phenotype in lung fibroblasts, possibly, through secretion of soluble mediators.

To identify the differentially changed soluble mediators in the CM from FOXF1-deficient HUVECs compared to control HUVECs, we used the Proteome Profiler Human Cytokine Array. CCL2, CXCL1, G-CSF, GM-SCF, ICAM-1 and IL-6 proteins were increased in CM from FOXF1-deficient HUVECs, whereas CXCL12, IL-8, IL-13 and IL-16 were unchanged (Fig. 4f). Since TNFα and IL-6 increase activation of fibroblasts during lung fibrosis^26^, we used blocking antibodies to inhibit increased levels of IL-6 or TNFα in CM from FOXF1-deficient HUVECs (Fig. 4g). Inhibition of either IL-6 or TNFα significantly decreased migration of CCL-19LU fibroblasts into the bottom chamber of Transwells containing CM from FOXF1-deficient HUVECs (Fig. 4g). To verify that the results are not limited to HUVEC cells, we also used HPAEC cells and confirmed that inhibition of either IL-6 or TNFα significantly decreased migration of CCL-19LU fibroblasts into the bottom chamber of Transwells containing CM from FOXF1-deficient HPAEC (Supplementary Fig. S10e, f). Thus, FOXF1-deficient endothelial cells stimulate the migration of lung fibroblasts in vitro by secreting IL-6 and TNFα.

### FOXF1 deficiency in endothelial cells increases the number of macrophages in the fibrotic lungs

**Fig. 5:**
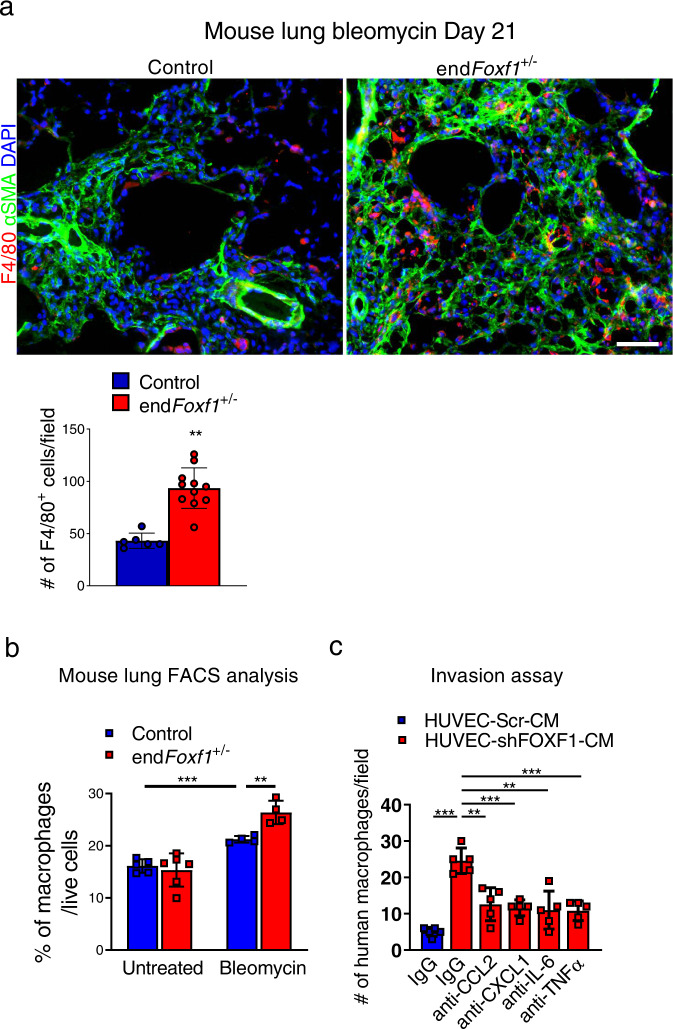
Inhibition of FOXF1 in EC increases the number of macrophages in the fibrotic lungs. **a** Increased number of macrophages in the lungs of end*Foxf1*^+/−^ (*n* = 11) mice compared to control (*n* = 6) mice at day 21 after bleomycin administration is shown using immunostaining for F4/80 (red) and αSMA (green) (control, *n* = 6 mice). Nuclei are counterstained with DAPI (blue). Bar = 100 μm. Average numbers of F4/80-positive cells were quantified using 10 random microscope fields per lung and presented as mean ± SD. **p = 0.0013. **b** Flow cytometry analysis shows increased percentage of macrophages in the lungs of bleomycin-treated end*Foxf1*^+/−^ mice compared to control mice. Macrophages were identified as CD45^+^ CD64^+^ CD11c^low/+^ (Untreated Control, *n* = 5; untreated end*Foxf1*^+/−^, *n* = 6; bleomycin-treated Control and end*Foxf1*^+/−^, *n* = 4). Data presented as mean ± SD. **c** Inhibition of CCL2, CXCL1, IL-6 and TNFα in CM from FOXF1-dificient HUVECs (shFOXF1-CM) using blocking antibodies attenuated microphage invasion in transwell assay. Human macrophages were incubated in the presence of CM from scrambled control (Scr-CM) or shFOXF1-transfected (sh*FOXF1*-CM) HUVECs. Invaded cells were counted in 5 random microscope fields and presented as mean ± SD. *n* = 5 samples per group. *p < 0.05, **p < 0.01, ***p < 0.001 by Student’s *T* test (two-tailed). CM condition medium. Source data are provided as a Source Data file.

Since numerous cytokines from the protein array, including IL-6, TNFα, CCL2 and CXCL1, regulate recruitment of macrophages during lung injury^26^, we examined the number of macrophages in Foxf1-deficient lungs. At day 21 after bleomycin injury, accumulation of macrophages in fibrotic lesions of bleomycin-treated *endFoxf1*^*+/−*^ lungs was increased compared to control lungs as shown by immunostaining for F4/80 (Fig. 5a) and Mac3 (Supplementary Fig. S12a). We also used flow cytometry analysis to demonstrate that the number of macrophages (CD45^+^ CD11c^low/+^ CD64^+^) was increased in bleomycin-injured *endFoxf1*^*+/−*^ lungs compared to control bleomycin-injured lungs (Fig. 5b and Supplementary Fig. S12b–c). Of note, no changes in the number of inflammatory cells, including macrophages, were reported in uninjured *endFoxf1*^*+/−*^ and control lungs^18^. We next determined whether increased secretion of pro-inflammatory mediators by FOXF1-deficient endothelial cells is important for macrophage migration in vitro. CM from control or FOXF1-deficient HUVECs was added to the bottom chambers of Transwells, and the migration of human macrophages from the upper chambers towards CM was assessed in the presence of blocking antibodies specific to CCL2, CXCL1, IL-6 or TNFα (Fig. 5c). Inhibition of these pro-inflammatory cytokines decreased migration of macrophages towards CM from FOXF1-deficient HUVECs compared to CM from control HUVECs (Fig. 5c and Supplementary Fig. S12d). Thus, FOXF1-deficient endothelial cells stimulate migration of macrophages by secreting multiple proinflammatory mediators.

### R-Ras is a direct transcriptional target of FOXF1

**Fig. 6:**
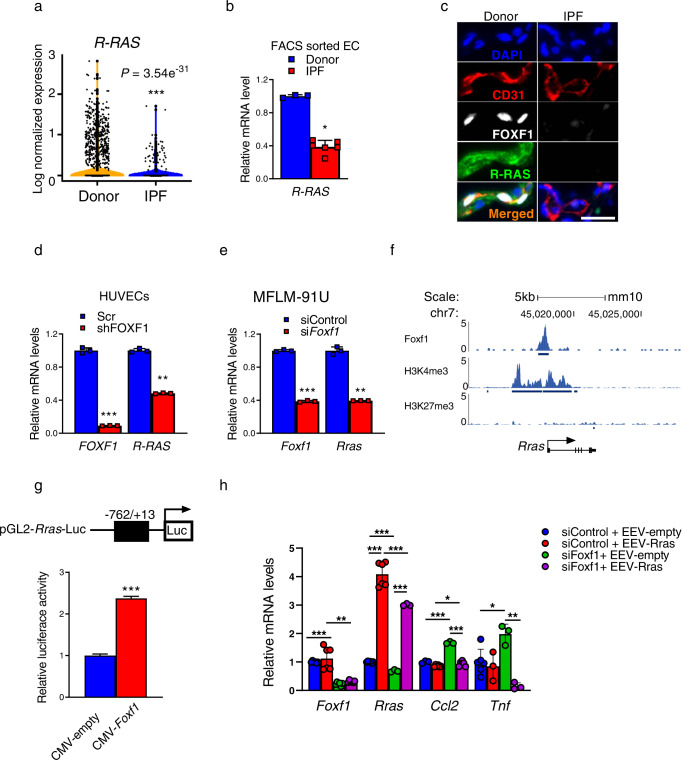
*R-Ras* is a direct transcriptional target of FOXF1. **a** Violin plots show decreased *R-RAS* mRNA in EC of IPF (*n* = 4 lungs) compared to donor (*n* = 8 lungs), GSE 122960 datasets. *R-RAS* expression was log normalized. **b** *R-RAS* mRNA was decreased in FACS-sorted ECs from IPF lungs (*n* = 6) compared to donor lungs (*n* = 3) as shown by qRT-PCR. Data presented as mean ± SD, *p = 0.238, Mann–Whitney Two-tailed test. **c** Immunostaining for R-RAS, FOXF1 and CD31 shows co-localization of FOXF1 and R-RAS in ECs of donor lungs (*n* = 5). Neither R-RAS nor FOXF1 are detected in ECs within IPF fibrotic lesions (*n* = 5). Nuclei are counterstained with DAPI. Bar = 20 μm. **d** qRT-PCR shows that shRNA-mediated knockdown of *FOXF1* (sh*FOXF1*) in HUVECs decreased *R-RAS* mRNA compared to control (Scr). *ACTB* mRNA was used for normalization (*n* = 3), **p < 0.01 and ***p < 0.001 by Student’s T test (two-tailed). **e** qRT-PCR shows that siRNA-mediated knockdown of *Foxf1* in mouse MFLM-91U ECs decreased *Rras* mRNA. *Actb* mRNA was used for normalization (*n* = 3). **f** ChIP-seq shows direct binding of FOXF1 protein to *Rras* promoter region in MFLM-91U cells. Binding of FOXF1 to *Rras* promoter is associated with H3K4me3 marks but not H3K27me3 marks. **g** Schematic drawing of the pGL2-Rras-Luc construct with the −762/+13 bp *Rras* promoter region containing the FOXF1-binding site (top panel). In co-transfection experiments, CMV-FOXF1 expression vector increased transcriptional activity of the −762/+13 *Rras* promoter region compared to CMV-empty vector (bottom panel), *n* = 3, ***p < 0.001. **h** Overexpression of R-Ras decreased *Ccl2* and *TNFα* mRNAs in mock-transfected cells and prevented upregulation of *Ccl2* and *TNFα* in cells transfected with Foxf1-specific siRNA. MFLM-91U cells were transfected with non-targeting siRNA (siControl) or si*Foxf1*, and EEV-empty vector or EEV-*Rras*. *Actb* mRNA was used for normalization (siControl+EEV-empty *Foxf1*, *n* = 6; siControl+EEV-empty *Rras*, *n* = 6; siControl+EEV-empty *Ccl2*, *n* = 3; siControl+EEV-empty *Tnf*, *n* = 6; siControl+EEV-Rras *Foxf1*, *n* = 6; siControl+EEV-Rras *Rras*, *n* = 6; siControl+EEV-Rras *Ccl2*, *n* = 6; siControl+EEV-Rras *Tnf*, *n* = 3; siFoxf1+EEV-empty *Foxf1*, *n* = 6; siFoxf1+EEV-empty *Rras*, *n* = 3; siFoxf1+EEV-empty *Ccl2*, *n* = 3; siFoxf1+EEV-empty *Tnf*, *n* = 3; siFoxf1+EEV-Rras *Foxf1*, *n* = 6; siFoxf1l+EEV-Rras *Rras*, *n* = 3; siFoxf1l+EEV-Rras *Ccl2*, *n* = 6; siFoxf1+EEV-Rras *Tnf*, *n* = 3), *p < 0.05, **p < 0.01, ***p < 0.001 by Student’s *t* test (two tailed). Source data are provided as a Source Data file.

FACS-sorted endothelial cells from bleomycin-treated end*Foxf1*^+/−^ lungs exhibited decreased expression of *Rras* (Fig. 4a), a critical mediator of endothelial barrier function and vascular repair after injury^27,28^. Consistent with decreased expression of *Rras* in endothelial cells, bleomycin-treated end*Foxf1*^+/−^ lungs displayed increased endothelial permeability as determined by Evans blue dye (Supplementary Fig. S13a). Since increased endothelial permeability contributes to lung fibrosis^29^ and end*Foxf1*^+/−^ mice had exacerbated lung fibrosis after bleomycin injury (Fig. 3), we examined whether FOXF1 regulates R-RAS in pulmonary endothelial cells. Based on scRNA-seq data analysis, *R-RAS* mRNA was decreased in human IPF endothelial cells compared to donor endothelial cells (Fig. 6a). We also FACS-sorted endothelial cells from IPF lungs and used qRT-PCR to demonstrate that *R-RAS* mRNA was decreased in IPF endothelial cells, coinciding with decreased expression of *FOXF1* mRNA (Fig. 6b). Immunostaining for R-RAS, FOXF1 and CD31 showed that FOXF1 co-localized with R-RAS in endothelial cells of donor lungs, indicating that both proteins are co-expressed in normal ECs (Fig. 6c). In contrast, neither R-RAS nor FOXF1 were detected in endothelial cells within fibrotic lesions of IPF lungs (Fig. 6c). In vitro shRNA-mediated knockdown of *FOXF1* in HUVECs decreased expression of *R-RAS* (Fig. 6d). These findings were confirmed in HPAEC and HPMEC cells (Supplementary Fig. S14a, b). In agreement with human data, shRNA-mediated knockdown of *Foxf1* in mouse MFLM 91U endothelial cells also decreased expression of *Rras* (Fig. 6e). Next, we used publicly available ChIP-seq dataset^25^ to show that FOXF1 protein directly bound to the *Rras* promoter region in endothelial cells (Fig. 6f). The FOXF1-binding region in *Rras* promoter had H3K4me3 but not H3K27me3 marks (Fig. 6f), suggesting that FOXF1 transcriptionally activates *Rras* gene promoter. To test this hypothesis, the −762/+13 bp *Rras* promoter region, containing the FOXF1-binding site identified by ChIP-seq (Fig. 6f), was cloned into the pGL2 luciferase reporter plasmid (Fig. 6g, upper schematic diagram). In co-transfection experiments, CMV-Foxf1 expression vector increased transcriptional activity of the −762/+13 *Rras* promoter region compared to CMV-empty vector (Fig. 6g). Thus, *Rras* is a direct transcriptional target of FOXF1 in endothelial cells.

Next, we examined whether R-RAS is involved in FOXF1-mediated regulation of CCL2, CXCL1, IL-6 and TNFα. We over-expressed R-Ras in FOXF1-deficient MFLM 91U endothelial cells in vitro (Fig. 6h and Supplementary Fig. S14a, b). Overexpression of R-Ras decreased *Ccl2* and *TNFα* mRNAs in mock-transfected cells and prevented upregulation of *Ccl2* and *TNFα* in cells transfected with Foxf1-specific siRNA (Fig. 6h). Overexpression of R-Ras did not prevent upregulation of *Cxcl1* and *IL-6* in FOXF1-deficient endothelial cells (Supplementary Fig. S14c). Altogether, our data suggest that FOXF1 in lung endothelium stimulates transcription of *Rras*, which inhibits expression of CCL2 and TNFα. FOXF1 inhibits CXCL1 and IL-6 independently of R-Ras.

### Transgenic overexpression of FOXF1 in endothelial cells decreases lung fibrosis after bleomycin-induced injury

**Fig. 7:**
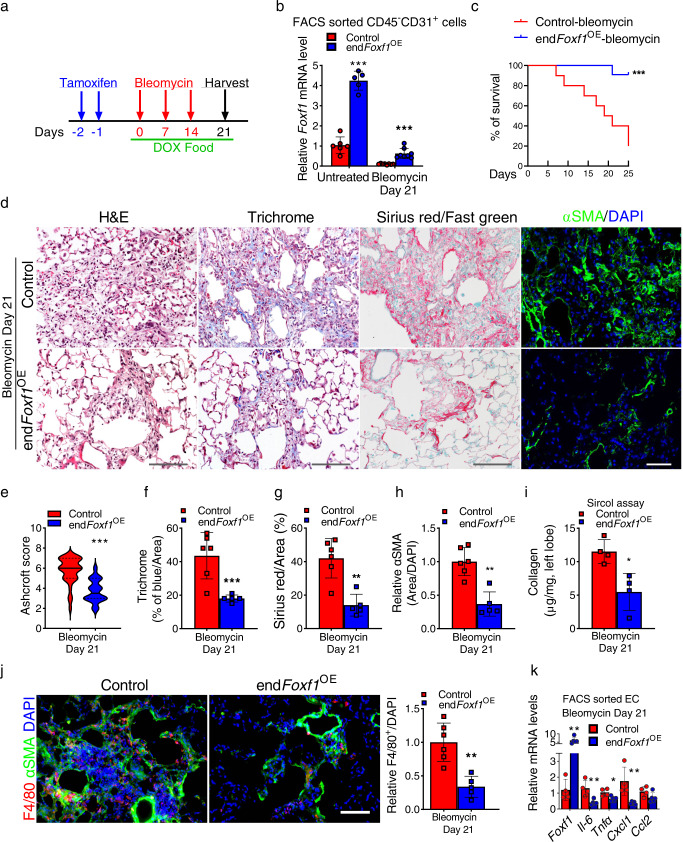
Endothelial-specific overexpression of FOXF1 before bleomycin injury decreases pulmonary fibrosis and improves survival of mice. **a** Schematic diagram shows bleomycin, tamoxifen (TAM) and doxycycline (DOX) treatments in end*Foxf1*^OE^ mice. **b** qRT-PCR shows that *Foxf1* mRNA is increased in lung ECs isolated from untreated and bleomycin-treated *endFoxf1*^*OE*^ mice compared to control mice. *Actb* mRNA was used for normalization (Untreated Control, *n* = 6; Untreated end*Foxf1*^OE^, *n* = 5; Bleomycin Control and end*Foxf1*^OE^, *n* = 8 mice per group), ***p < 0.001. **c** Endothelial-specific overexpression of FOXF1 increases mouse survival after bleomycin injury (*n* = 10), **p = 0.001, Log-rank (Mantel–Cox) test. **d** Decreased fibrosis in lungs of bleomycin-treated *endFoxf1*^*OE*^ mice is shown by H&E, Trichrome, Sirius red/fast green staining and immunostaining for αSMA (green). Mouse end*Foxf1*^OE^ (*n* = 5) and control lungs (*n* = 5) were harvested at day 21 after bleomycin treatment. Bar = 100 μm. **e**–**h** Assessment of fibrotic lesions in *endFoxf1*^*OE*^ (*n* = 5) lungs is presented as Ashcroft score ***p < 0.0001 (**e**), the percent of Trichrome-positive **p = 0.0058 (**f**) Sirius red-positive. **p = 0.0011 (**g**), and αSMA-positive areas in the lungs (Control, *n* = 6), **p = 0.0013. Areas positive for collagen depositions were quantified in 10 random fields per lung using Nikon’s NIS-Elements AR software (**h**). **i** Lung collagen depositions were quantified by Sircol collagen assay. Left lung lobes from *endFoxf1*^*OE*^ (*n* = 4) and control mice (*n* = 4) were used. *p = 0.0286. *t* test (two tailed). **j** Decreased number of macrophages in the lungs of bleomycin-treated end*Foxf1*^OE^ mice (*n* = 5) is shown using immunofluorescent staining with anti-F4/80 antibodies (red) and anti-αSMA antibodies (green) at day 21 after bleomycin administration (Control, *n* = 6). Nuclei are counterstained with DAPI (blue). Bar = 100 μm. Average numbers of F4/80-positive cells were quantified using 10 random microscope fields per lung and presented as mean ± SD. **p = 0.0013. *t* test (two tailed). **k** Endothelial overexpression of *Foxf1* decreases *mRNA* levels of pro-inflammatory genes in FACS sorted EC as shown by qRT-PCR. mRNA was extracted from control and bleomycin-treated end*Foxf1*^OE^ mice on day 21. *Actb* mRNA was used for normalization. Control, *n* = 4; end*Foxf1*^OE^, *n* = 5. Data presented as mean ± SD. *p < 0.05, **p < 0.01, ***p < 0.001 by Student’s *t* test (two tailed). Source data are provided as a Source Data file.

Since conditional deletion of FOXF1 in endothelial cells increased pulmonary fibrosis after chronic bleomycin injury (Fig. 3), we examined whether overexpression of FOXF1 in endothelial cells was sufficient to inhibit lung fibrosis. To test this hypothesis, we generated an inducible, EC-specific FOXF1 overexpression mouse model (*Pdgfb-CreER*^*tg/+*^*;* LSL-rtTA^tg/+^; TetO-*Foxf1*^*tg/+*^ mice*;* abbreviated as end*Foxf1*^OE^) (Supplementary Fig. S15a). Upon tamoxifen administration, Cre-mediated recombination of LoxP-floxed stop codon (LSL) results in rtTA expression in endothelial cells. In the presence of doxycycline, rtTA binds to and activates the TetO_7_-CMV promoter driving expression of the mouse *Foxf1* transgene (Supplementary Fig. S15a). Thus, combined administration of tamoxifen (Tam) and doxycycline (Dox) causes increased expression of FOXF1 in endothelial cells of end*Foxf1*^OE^ mice (Fig. 7a). FACS-sorted endothelial cells from Tam- and Dox-treated end*Foxf1*^OE^ lungs had a 4-fold increase in *Foxf1* mRNA compared to controls (Fig. 7b). Without bleomycin injury, the Tam- and Dox-treated end*Foxf1*^OE^ mice had normal lung architecture and capillary density as shown by H&E staining and immunostaining for CD31 (PECAM-1) (Supplementary Fig. S15b). To induce pulmonary fibrosis, Tam- and Dox-treated end*Foxf1*^OE^ and single transgenic (control) mice were given three weekly IT injections of bleomycin (Fig. 7a). While bleomycin treatment decreased *Foxf1* mRNA in FACS-sorted endothelial cells from both groups of mice, *Foxf1* expression was higher in end*Foxf1*^OE^ ECs compared to control ECs (Fig. 7b). Transgenic overexpression of *Foxf1* increased survival of mice after bleomycin injury with 95% of end*Foxf1*^*OE*^ mice surviving the injury, but only 20% of control single-transgenic littermates being alive 25 days after the last bleomycin treatment (Fig. 7c). Fibrotic lung remodeling was decreased in bleomycin-treated end*Foxf1*^*OE*^ mice compared to controls as demonstrated by Ashcroft score, Trichrome staining, Sirius red/Fast green staining and immunostaining for αSMA (Fig. 7d–h). Biochemical quantification of lung collagen amounts using Sircol assay was consistent with decreased fibrosis in end*Foxf1*^*OE*^ lungs (Fig. 7i). Moreover, endothelial over-expression of *Foxf1* decreased recruitment of macrophages into bleomycin-treated lungs and decreased expression of pro-inflammatory genes in total lung RNA (Fig. 7j, k). Thus, overexpression of FOXF1 in endothelial cells prior to bleomycin injury attenuates pulmonary fibrosis and improves survival after bleomycin-induced lung injury.

**Fig. 8:**
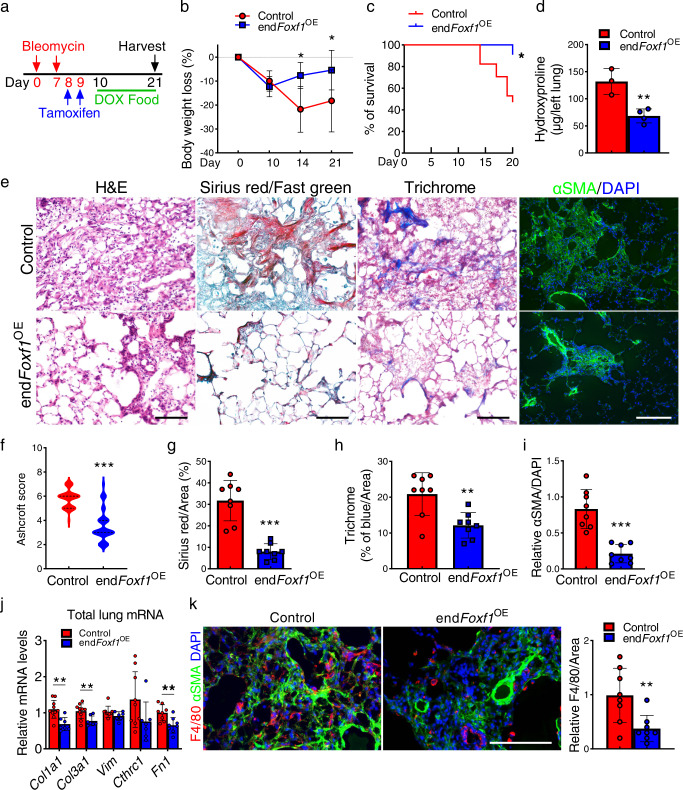
Endothelial-specific overexpression of FOXF1 during fibrotic stage decreases lung fibrosis and increases survival of mice after bleomycin-induced injury. **a** Schematic diagram shows bleomycin, tamoxifen (TAM) and doxycycline (DOX) treatment in end*Foxf1*^OE^ mice. **b** Endothelial-specific overexpression of FOXF1 decreases bodyweight loss in bleomycin-treated end*Foxf1*^OE^ mice (Control, *n* = 12; end*Foxf1*^OE^, *n* = 12 for days 0 and 21, *n* = 4 for day 10, *n* = 6 for day 14), *p < 0.05, two-way ANOVA with the Geisser–Greenhouse correction. **c** Endothelial-specific overexpression of FOXF1 increases mouse survival after bleomycin injury (Control, *n* = 11; end*Foxf1*^OE^, *n* = 17), *p < 0.05, Log-rank (Mantel–Cox) test. **d** Decreased collagen depositions in bleomycin-treated *endFoxf1*^*OE*^ lungs is shown by Hydroxyproline assay. Left lung lobes from *endFoxf1*^*OE*^ (*n* = 4) and control (*n* = 3) mice were used, **p = 0.0061, t test (two tailed). **e** Decreased fibrosis in bleomycin-treated *endFoxf1*^*OE*^ lungs is shown by H&E, Sirius red/fast green, Trichrome, and immunostaining for αSMA. Lungs were harvested at day 21 after bleomycin treatment (*n* = 8 per group). Bar = 100 µm. **f**–**i** Ashcroft score of *endFoxf1*^*OE*^ lungs (***p < 0.0001) is consistent with the percent of Sirius red-positive (***p < 0.0001), Trichrome-positive (**p = 0.0031) and αSMA-positive areas (***p < 0.0001). Areas positive for collagen depositions were quantified in 10 random fields per lung using Nikon NIE CIC Analysis Elements software. *n* = 8 mice per group. **j** Endothelial overexpression of *Foxf1* at day 10 after bleomycin administration decreases *mRNA* levels of pro-fibrotic genes in total lung RNA as shown by qRT-PCR. Total lung mRNA was extracted from control and bleomycin-treated end*Foxf1*^OE^ mice on day 21. *Actb* mRNA was used for normalization (Control *Col1a1* and *Col3a1*, *n* = 10; Control *Vim*, *n* = 9; Control *Cthrc1*, *n* = 9; Control *Fn1*, *n* = 8; end*Foxf1*^OE^ *Col1a1*, *Col3a1*, *Vim* and *Cthrc1*, *n* = 7; end*Foxf1*^OE^ *Fn1*, *n* = 8). **k** Decreased number of macrophages in the lungs of bleomycin-treated end*Foxf1*^OE^ mice is shown using immunofluorescent staining with anti-F4/80 antibodies (red) and anti-αSMA antibodies (green) at day 21 after bleomycin administration, ***p < 0.0001. Nuclei are counterstained with DAPI (blue). *n* = 8 mice per group. Data presented as mean ± SD, *p < 0.05, **p < 0.01, ***p < 0.001, Student’s *t* test (two tailed). Source data are provided as a Source Data file.

Next, we assessed whether increasing endothelial FOXF1 in already established lung fibrosis will improve the outcomes. Overexpression of FOXF1 in endothelial cells at day 10 after bleomycin injury decreased body weight loss, improved mice survival, and decreased collagen depositions in *endFoxf1*^*OE*^ mice compared to bleomycin-injured control mice (Fig. 8a–i). Based on Flexivent measurements of lung mechanics, many functional parameters in *endFoxf1*^*OE*^ mice were improved, including increased lung compliance, total lung capacity and decreased tissue resistance (Supplementary Fig. S16a–h). Finally, overexpression of FOXF1 in endothelial cells at day 10 after bleomycin injury, decreased expression of pro-fibrotic genes in total lung RNA and inhibited recruitment of macrophages to the lung tissue (Fig. 8j, k). Altogether, overexpression of FOXF1 in endothelial cells either prior to or after bleomycin injury attenuates pulmonary fibrosis and improves mice survival after the injury.

### Nanoparticle delivery of non-integrating *Foxf1* expression-vector into the lung endothelium attenuates pulmonary fibrosis

**Fig. 9:**
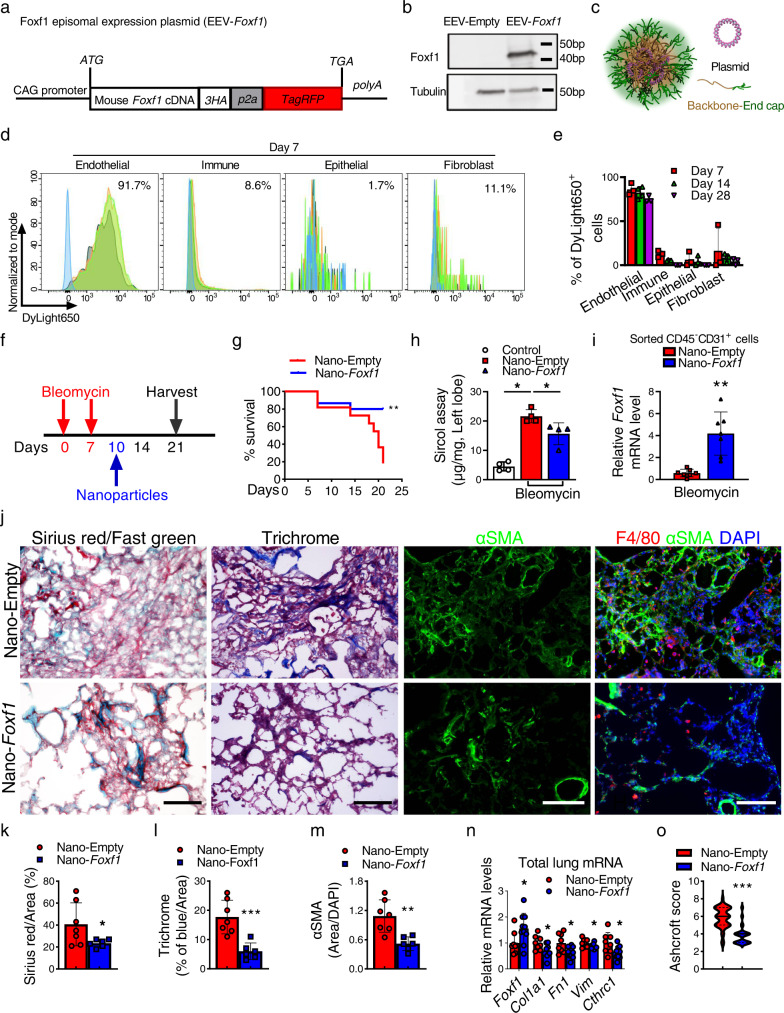
Nanoparticle delivery of non-integrating *Foxf1* expression vector into the lung endothelium attenuates pulmonary fibrosis. **a** Diagram of the FOXF1 episomal plasmid (EEV-*Foxf1*). **b** Western blot shows increased FOXF1 protein after transfection of EEV-*Foxf1* plasmid into FOXF1-negative HEK-293T cells. **c** Schematic of PBAE nanoparticles loaded with plasmid DNA. All elements of images were created using Autodesk 3ds Max 2020 (Autodesk, version 2020). **d** FACS analysis of mouse lungs show the presence of labeled nanoparticles in endothelial cells but not in other cell types at day 7 after I.V. administration (*n* = 3). **e** FACS analysis shows nanoparticles in lung ECs at different time-points after bleomycin injury (*n* = 5 mice per group). **f** Diagram shows nanoparticle I.V. delivery to mice at day 10 after bleomycin administration. **g** Kaplan–Meier analysis shows increased survival of bleomycin-injured mice after nanoparticle delivery of EEV-*Foxf1* plasmid (Nano-*Foxf1*, *n* = 15) compared to EEV-Empty (Nano-Empty, *n* = 11), **p < 0.005, Log-rank (Mantel–Cox) test. **h** Sircol assay shows decreased collagen depositions in Nano-*Foxf1*-treated lungs (*n* = 4 mice per group), *p < 0.05, ***p < 0.001, Mann–Whitney Two-tailed test. **i** *Foxf1* mRNA is increased in FACS**-**sorted lung ECs from Nano-*Foxf1* treated mice (*n* = 7), *p < 0.05, **p < 0.005, Student’s *t* test (two tailed). **j** Decreased lung fibrosis in Nano-*Foxf1*-treated mice is shown with Sirius red/fast green, Masson’s trichrome, and immunostaining for αSMA. Decreased number of macrophages in Nano-*Foxf1*-treated lungs is shown by immunostaining for F4/80 and αSMA at day 21 after bleomycin administration (*n* = 6 mice per group). Bar = 100 μm. **k**–**n** Quantification of fibrotic lesions in mouse lungs is shown as the percent of Sirius red-positive (*k*), Trichrome-positive (*l*) and αSMA (*m*) areas. Areas positive for collagen depositions were quantified in 10 random microscope fields per lung (Nano-Empty, *n* = 7; Nano-*Foxf1*, *n* = 6). **n** Nanoparticle delivery of *Foxf1* at day 10 after bleomycin administration decreases *mRNA* levels of pro-fibrotic genes in total lung RNA as shown by qRT-PCR. (Nano-Empty control, *n* = 8, and Nano-*Foxf1-*treated, *n* = 10, on day 21 after bleomycin administration*. Actb* mRNA was used for normalization. **o** Decreased Ashcroft score in *nano-Foxf1* treated lungs (*n* = 10) compared to control nano-Empty (*n* = 8). Data presented as mean ± SD. *p < 0.05, **p < 0.01, ***p < 0.001 by Student’s *t* test (two tailed). Source data are provided as a Source Data file.

Since genetic overexpression of *Foxf1* in endothelial cells attenuated pulmonary fibrosis, we next tested whether nanoparticle delivery of *Foxf1* cDNA into endothelial cells can be a therapeutic approach to inhibit lung fibrosis. Mouse *Foxf1* cDNA was cloned into non-integrating self-replicating episomal EEV vector to generate EEV-*Foxf1* construct (Fig. 9a). Transfection of EEV-*Foxf1* plasmid into Foxf1-negative HEK-293T cells increased the FOXF1 protein levels in vitro as shown by Western blot (Fig. 9b). To deliver EEV-*Foxf1* plasmid to endothelial cells in vivo, we utilized the poly β-amino esters (PBAE) polymer^30,31^, which formed stable nanoparticles in complex with plasmid DNA (Fig. 9c and Supplementary Fig. S17a, b). The hydrodynamic average diameter of the PBAE nanoparticles loaded with EEV-Foxf1 plasmid DNA (Nano-Foxf1) was 146.27 ± 9.54 nm (Supplementary Fig. S17c), whereas the average surface charge of the Nano-Foxf1 was 28.3 ± 1.71 mV (Supplementary Fig. S17d). In vitro treatment of HEK-293T cells with nanoparticles containing EEV-Foxf1 plasmid resulted in the efficient expression of red fluorescent protein (RFP) in vast majority of cells (Supplementary Fig. S17e). Next, fluorescently-labeled PBAE nanoparticles with either EEV-Foxf1 or EEV-Empty (control) plasmids were delivered to bleomycin-treated mice (Fig. 9d, e and Supplementary Fig. S17f, g) via tail vein on the same day as bleomycin administration. Using FACS analysis of enzymatically digested lung tissue, nanoparticles were detected in ~92% of lung endothelial cells (CD31^+^/CD45^−^) (Fig. 9e). Nanoparticles-mediated targeting of epithelial (CD326^+^/CD45^−^/CD31^−^), immune (CD45^+^/CD31^−^) and mesenchymal (CD31^−^/CD45^−^/CD326^−^) cells was ineffective (Fig. 9d, e). Nanoparticles were still detected in ~50–70% of pulmonary endothelial cells at day 28 after single nanoparticle delivery (Fig. 9e and Supplementary Fig. S17g). Administration of EEV-*Foxf1*-nanoparticles increased *Foxf1* mRNA in FACS-sorted lung endothelial cells as demonstrated by qRT-PCR (Fig. 9f). Treatment with nanoparticles containing pEEV-*Foxf1* vector on the same day as bleomycin injury significantly increased survival of mice as shown by Kaplan–Meier curve (Supplementary Fig. S18a) and decreased collagen depositions in the lung tissue as shown by H&E and Sirius red staining (Supplementary Fig. S18b).

Next, we assessed the therapeutic efficacy of EEV-*Foxf1*-nanoparticles after lung injury. Mice were treated with EEV-*Foxf1*-nanoparticles at day 10 after bleomycin administration (Fig. 9f) when the fibrosis lung remodeling is already present (Fig. 2a). Nanoparticle delivery of Foxf1 into endothelial cells improved mice survival (Fig. 9g), prevented the loss of body weight after bleomycin injury (Supplementary Fig. S19a), and decreased lung collagen depositions quantified by Sircol assay (Fig. 9h). Decreased collagen amounts in EEV-Foxf1-treated lungs coincided with increased *Foxf1* mRNA in FACS-sorted lung endothelial cells (Fig. 9i). Decreased fibrotic remodeling in EEV-Foxf1-treated lungs was detected and quantified based on Sirius red/Fast green staining, Trichrome staining, and immunostaining for αSMA (Fig. 9j–m). In addition, nanoparticle delivery of Foxf1 into endothelial cells at day 10 after bleomycin injury, reduced macrophage infiltration into fibrotic regions (Fig. 9j, right panels and Supplementary Fig. S19l), decreased expression of pro-fibrotic genes in total lung RNA (Fig. 9n), decreased Ashcroft score (Fig. 9o), improved lung compliance and total lung capacity in EEV-Foxf1-treated mice (Supplementary Fig. S19b–j) and arterial oxygenation (Supplementary Fig. S19k). Altogether, nanoparticle delivery of *Foxf1* cDNA into endothelial cells on the day of bleomycin injury or during fibrotic stage after the injury inhibits pulmonary fibrosis and improves mice survival.

## Discussion

Significant changes in gene expression among epithelial and mesenchymal cellular populations of IPF lungs have been recently identified by scRNA-seq^7,19,32^. Carraro et al. identified unique subclusters of lung epithelial cells that were specific to IPF lungs^32^. Likewise, other studies used scRNA-seq to examine gene expression signatures in distinct epithelial and mesenchymal cell types during pulmonary fibrogenesis^7^. Novel population of profibrotic alveolar macrophages was identified exclusively in patients with fibrosis^19^. Focusing on fibrosis-associated changes in pulmonary endothelial cells, we found in this study that one of the most downregulated transcription factors in human IPF and in the mouse bleomycin-treated lungs is FOXF1. The FOXF1 is highly enriched in lung endothelial cells compared to endothelial cells of other organs including liver, pancreas, brain, heart, and kidney^21,33^. FOXF1 is required for embryonic development of pulmonary vasculature by activating VEGF, BMP9 and TIE-2 signaling pathways^12,16,17^. FOXF1 stimulates lung repair and regeneration by regulating genes critical for extracellular matrix remodeling, inflammation, and endothelial barrier function^18,25,34–36^. FOXF1 is identified as an anti-fibrotic factor that regulates key myofibroblast functions by preventing CDH2-CDH11 cadherin switching and by reducing lung inflammation during pulmonary fibrosis^37^.

An important contribution of the current studies is that FOXF1 transcriptionally regulates *Rras* gene expression as evidenced by direct binding of FOXF1 to the *Rras* promoter region, leading to transcriptional activation of the *Rras* promoter by CMV-Foxf1 expression plasmid. The small guanosine triphosphate hydrolase (GTPase) Ras-related protein (R-Ras) regulates a lot of different cellular processes, including cell adhesion to the extracellular matrix via integrin activation^38^, endothelial cell adhesion via VE-cadherin stabilization^27^, cell survival via PI3K/AKT pathway^39^ and angiogenesis via inhibition of the VEGF receptor 2 (VEGFR2) internalization^40,41^. Published studies demonstrated that R-Ras is important for blood vessel stabilization by stimulating signaling between endothelial cells and pericytes^27,42^. Deletion of *Rras* gene in mice resulted in increased vascular permeability in various models of angiogenesis^27,43^. These studies are consistent with the phenotype of the mice with homozygous endothelial *Foxf1* deletion that succumbed from lung hemorrhage and edema within a month after *Foxf1* deletion^18^. FOXF1 maintains endothelial barrier function by stimulating expression of VEcadherin and S1PR1^18^. Therefore, increased endothelial permeability can contribute to aberrant inflammation and increased fibrosis in endFoxf1+/− lungs. Since R-RAS plays an important role in blood vessel regeneration, maturation, and stability, it is important to identify molecular mechanisms regulating R-Ras signaling during lung fibrosis. We report here that overexpression of R-RAS prevents upregulation of CCL2 and TNFα in FOXF1-deficient endothelial cells, suggesting that FOXF1 inhibits CCL2 and TNFα via R-RAS. Even though, CCL2 and TNFα are produced by multiple cell types, the use of neutralizing CCL2 or TNFα antibodies in co-culture of endothelial cells and fibroblasts decreased activation of fibroblasts, indicating the importance of angiocrine functions of EC. Since both CCL2 and TNFα promote macrophage migration to the lung tissue^44^, decreased expression of R-RAS can contribute to aberrant accumulation of macrophages in bleomycin-treated *endFoxf1*^*+/*−^ lungs.

Our studies support the notion that increasing expression of FOXF1 in endothelial cells through nanoparticle delivery of *Foxf1* cDNA can be considered to alleviate pulmonary fibrosis. The major drawbacks of the conventional therapeutics for respiratory disorders include the insufficient drug concentrations at pathological lesions, lack of cell-specific targeting, and various bio-barriers in the conducting airways and alveoli^45^. To address these critical issues, various nanoparticle delivery systems have been developed to serve as carriers of specific drugs, DNA expression vectors, and RNAs^46^. Although intratracheal administration is an attractive method of DNA/RNA delivery to the lung tissue, specific targeting of endothelial cells from the air surface is ineffective since it requires crossing the epithelial barrier without unloading the therapeutic cargo. We found here that pulmonary endothelial cells can be efficiently and specifically deliver non-integrating Foxf1 plasmid by PBAE nanoparticles through blood circulation. The size and cationic charge of our PBAE nanoparticles are similar to the recently reported cationic polyplexes that contained PEI, fatty acids, cholesterol and PEG (PEI/PEG)^47^, and demonstrated an efficient targeting of pulmonary endothelial cells after intravenous administration to adult mice. Since the recent successful nanoparticle delivery of different cargos have been effective in stimulating lung repair after neonatal hyperoxic injury^48^ and in inhibiting fibrotic lung remodeling in mouse model of ACD/MPV^49^, nanoparticle gene therapy has a promise to restore endothelial function in fibrotic lung diseases. However, it is unclear at the moment whether increasing FOXF1 levels through nanoparticle gene therapy or recently discovered FOXF1-activating small molecule compound^50^ will have beneficial effect in human IPF. The efficacy of FOXF1 targeting in IPF can only be determined in clinical trials.

In summary, FOXF1 functions in EC to maintain R-Ras signaling and prevent secretion of pro-inflammatory mediators from EC, leading to decreased activation of lung fibroblasts. Our studies suggest the FOXF1-activating therapies can be considered to improve the long-term outcomes in IPF patients.

## Methods

### Ethical statement

The Institutional Review Board of the Cincinnati Children’s Hospital Medical Center (Federalwide Assurance #00002988) approved all the studies with human tissue samples (IRB protocol #2017-4321). Human lung tissue specimens were obtained from tissue repository at University of Cincinnati Medical Center that provides de-identified human biospecimen procurement and banking services in support of basic, translational, and clinical research. Total number of ten lung tissue samples were received, including six males and four females. All patients signed informed consent prior to tissues collection. All animal studies were approved by Cincinnati Children’s Research Foundation Institutional Animal Care and Use Committee and covered under our animal protocol (IACUC2016-0070). The Cincinnati Children’s Research Foundation Institutional Animal Care and Use Committee is an AAALAC and NIH accredited institution (NIH Insurance #8310801). All mice were kept under SPF (specific-pathogen free) conditions in 12/12 light/dark cycle, 18–23 °C and 40–60% humidity. Both males and females were used for studies.

### Transgenic mice and bleomycin-induced fibrosis model

Generation of endothelial-cell specific Foxf1 heterozygous mice: *Foxf1*^fl/fl^ mice^16^ were crossed with *Pdgfb-CreER*^*tg/−*^ mice^51^ to generate *Pdgfb-CreER/Foxf1*^*fl/+*^ (abbreviated as end*Foxf1*^*+/−*^) mice in C57BL6 genetic background. To delete 1 allele of FOXF1 from endothelial cells, tamoxifen (3 mg; Sigma) was given as oral gavage on 2 consecutive days to 6–8 weeks old mice. Generation of *TetO7-HA-mFoxf1*^*tg/+*^ mice: The 5’- HA-tagged coding region of mouse *Foxf1* gene in a Shuttle vector^52,53^ was subcloned into a TetO7-CMV vector. The 2 kb DNA fragment containing TetO7-CMV-HA-Foxf1 was microinjected into the pronucleus of fertilized mouse eggs. Transgenic mice were identified by PCR with primers flanking HA tag and exon 2 of *Foxf1* gene (Supplementary Table 1). Generation of endothelial-cell specific FOXF1 overexpression mice: *TetO7-HA-*m*Foxf1*^tg/+^ mice were crossed with *Pdgfb-CreER*^*tg/−*^^51^ mice and *Rosa26-*LSL-rtTA^tg/+^ to generate *Pdgfb-CreER/Rosa26-*LSL-rtTA/*TetO7-HA*-m*Foxf1* mice in C57BL6/129sv/FVB hybrid genetic background. To induce FOXF1 overexpression in endothelial cells, tamoxifen (3 mg; Sigma) was given as oral gavage on 2 consecutive days to 8–9 weeks old mice, followed by maintenance of mice on doxycycline chow for the duration of the experiment. To induce pulmonary fibrosis, mice were administered 2 or 2.5 U/kg of bleomycin sulfate (EMD Biosciences) intratracheally (I.T.) once a week for 3 weeks. The control group of mice (Uninjured) were administered PBS I.T. the same way as bleomycin.

### 10× Genomics single cell RNA-Seq data analysis

For CCHMC data, we have used donor (2 males and 1 female) and IPF (2 males) lung tissue samples. For NW data, we have re-analyzed the deposited scRNAseq data of lung tissue samples previously published by Reyfman et al.^19^ (Northwestern University (NW) data), focusing on individuals with IPF (3 males and 1 female) and donors without IPF (7 females and 1 male) and excluding samples from individuals with SSc-ILD, myositis and HP. Description of total number of samples was provided in Supplementary Table S4. For human lung tissue procurement, sample processing, and single-cell sequencing methods, we have used previously described methods^19^. Briefly, explanted lungs were acquired from donors with end-stage IPF lung disease undergoing transplant or from rejected control donor lungs. The explanted lungs were sliced and washed with cold sterile PBS. After visible vessels and airway structures were removed, lung tissues were mechanically minced, enzymatically digested, using an enzymatic cocktail containing 60 ml dispase (5 U/ml, Stemcell Technologies), 900 µl liberase (1 g/l, Sigma Aldrich), 0.03 g DNase (Sigma Aldrich) at 37 °C for 45 min. GEM generation and barcoding, and cDNA library preparation were completed by the CCHMC Gene Expression Core using the Chromium Single Cell 3’ Reagent Kit (10X Genomics version 2.0). Sequencing was performed on NovaSeq 6000 at a depth of 300–450 million reads. Read alignment and gene-expression quantification of data were performed using the CellRanger pipeline (10X Genomics version 2.1.1). Human scRNA-seq data was aligned to HG19 and mouse scRNA-seq data was aligned to mouse genome (mm10). Additional IPF scRNA-seq data was downloaded from GEO (https://www.ncbi.nlm.nih.gov/geo/; GSE122960). Cells with at least 500 expressed genes (UMI > 0) and less than 10% of UMIs mapping to mitochondrial genes were included for downstream analysis. Genes expressed in at least two cells in each dataset were included. Datasets were integrated with Harmony^22^ and downstream analyses were performed in R (version 3.6.1) using custom scripts, SINCERA^54^, and Seurat (version 3)^55^. The expression of a gene in a cell was measured by its UMI counts in the cell normalized by the total number of UMIs in the cell. Principal-component analysis was performed for dimension reduction. Reduced dimensions were used for cell cluster identification using the Jaccard-Louvain clustering algorithm^56^. Clusters were mapped to cell types based on the expression of known marker genes. Cluster-specific differentially expressed genes were identified using a binomial based differential expression test implemented in the SINCERA pipeline^54^. Genes with *p* value <0.05 and effect size >2 expressed in >20% of the cells in each cluster were considered significant. Transcription factors were mapped using “Signature comparison” in LGEA tool-box^57^ (https://research.cchmc.org/pbge/lunggens/tools/sigcomp.html/) and functional enrichment analyses were performed using Toppfun in ToppGene suite (https://toppgene.cchmc.org/).

### Immunostaining and collagen content

Lung paraffin sections were used for H&E staining, and paraffin and frozen sections were used for immunofluorescence as previously described^18^. Lung tissue sections were from both female and male patients. For mouse experiments both males and females were included in equal numbers. The list of antibodies used for immunostaining is included in Supplementary Table S2. For immunofluorescence imaging, secondary antibodies conjugated with Alexa Fluor 488, Alexa Fluor 594, or Alexa Fluor 647 (Invitrogen/Molecular Probes) were used as described^18,58^. Cell nuclei were counterstained with DAPI. Images were obtained using a Nikon NIE upright CIC widefield microscope. Quantification of lung collagen content was performed using the Sircol collagen assay (Biocolor, Carrick Fergus, UK), Hydroxyproline assay (Jiancheng Bioengineering Institute, Nanjing, China), Sirius Red/Fast green (Chondrex, Inc. Redmond, WA, USA) and Masson’s trichome assay (Poly Scientific. Bay Shore, NY, USA) following the manufacturer’s protocols. Quantification of collagen from Sirius red and trichome staining was done using the Nikon NIE CIC Analysis Elements Workstation (NIS-Elements AR, advanced research,ver.5).

### Cell lines, qRT-PCR, and western blot

Human endothelial HUVEC cells (Lonza, #C2519A) were cultured in EGM2/EBM2 (Lonza) growth medium, human Pulmonary Microvascular Endothelial Cells (HPMEC, ##CC2527) and human Pulmonary Artery Endothelial Cells (HPAEC, #CC2530) were cultured in ECM Medium (ScienCell). HUVEC, HPAEC and HPMEC cells were from pooled donors. Mouse endothelial MFLM-91U cells (Seven Hills, #AMFLM-91U) were cultured in DMEM (Gibco), human fibroblasts CCD-19Lu (ATCC, #CCL-210) were cultured in EMEM (ATCC), human macrophages (Lonza, #4W-700) were cultured in X-VIVO^15,59^ (Lonza). Human CCD-19Lu fibroblast cell line (ATCC) was isolated from a 20-year-old normal female lung. Mouse MFLM-91U Cell Line (Seven Hills) was isolated from murine fetal lung mesenchyme without specifying the sex. A cocktail of siRNAs was used to knockdown *Foxf1* (Horizon, Cat# M-043272-00-0010) in MFLM-91U cells using Dharmafect transfection reagent 1 according to the manufacturer’s protocol (Dharmacon). A cocktail of non-targeting siRNAs was used as control (Horizon, Cat# D-001810-01-20). Cells were collected for RNA and protein extraction 48 h post transfection. In vitro rescue experiments were performed by dual transfection of si*Foxf1* and CMV-*Rras* plasmid (Addgene plasmid # 102864) into MFLM-91U cells using Dharmafect Duo transfection reagent (Dharmacon). Empty plasmid and non-targeting siRNA were used as control. qRT-PCR was carried out using Taqman probes listed in Supplementary Table S1. Western blots were performed as previously described^60^ with antibodies against FOXF1 (1:500, R&D), α-Tubulin (sc-8035, 1:5000, Santa Cruz). For stable knockdown of FOXF1, the HUVEC, HPMEC and HPAEC were transduced with TRC Lentiviral Human FOXF1 shRNA (clone ID: TRCN0000013953, Horizon). GIPZ non-silencing lentiviral shRNA (Cat #: RHS4346, Horizon) was used as control.

### RNAscope in situ hybridization assay

Assay was performed according to a protocol developed by Advanced Cell Diagnostics (ACD)^61^, using in situ probes designed by ACD, the RNAscope Multiplex Fluorescent Reagent Kit (v.2) and Opal dyes (Akoya Biosciences, 1:500 dilution for Opal 570 and 690 dyes, 1:1000 dilution for Opal 520 dyes). Nuclei were counterstain with DAPI, and tissue sections were mounted in Prolong Gold antifade reagent (Invitrogen). Proprietary (ACD) probes used were: Human, Hs-EDNRB(528301), Hs-EDN1 (459381), CLDN5-C2(517141-C2), Hs-FOXF1(505741-C3), Hs-CA4(438561), Hs-GJA5(471431), Hs-CPE(45410); Mouse, Mm-Foxf1(473051), Mm-Ednrb-C2(473801-C2), Mm-Aplnr-C2(436171-C2), Mm-Cldn5-C3(491611-C3), Mm-Car4-C2(468421-C2), Mm-Gja5-C2(518041-C2),Mm-Ptgds-C2(492781-C2). Tissue slides were photographed with a wide-field Nikon i90 or Nikon confocal microscope and quantified using the Nikon’s NIS-Elements AR (Advanced Research, ver. 5) software.

### Cloning of the mouse *Rras* promoter region and luciferase assay

The mouse *Rras* promoter corresponding to the −762bp to 13bp region, was PCR amplified from C57/B6 gDNA using the following primers (5’ to 3’): Fwd. GTCTGAGCTATCAACCGCATCCTTC; Rev. TCATGTCGCCACCGCTGCTG. The promoter region was cloned into the Acc651 and XhoI sites of the pGL2 luciferase reporter plasmid (Promega). The dual-luciferase reporter assay was performed on Hek293T cells, co-transfected with the luciferase reporter and with a CMV-empty or CMV-Rras overexpression plasmid.

### Flow cytometry

Flow cytometry experiments were conducted as previously described^34^ using antibodies listed in Supplementary Table S3. Stained cells were analyzed using FACSCanto II or LSR II (BD Biosciences). Stained cells were sorted using cell sorting (five-laser FACSAria II; BD Biosciences). Specific cell subsets were identified using flow cytometery markers recommended by American Thoracic Society:^62^ macrophages, CD45^+^ CD11c^low/+^ CD64^+^, neutrophils, CD45^+^ SSC^hi^ Ly6G^+^; monocytes, CD45^+^ CD11b^+^ CD64^+^^63–65^. Data analyses were performed using FlowJo Software version 10.8.0. and FACSDiva 9.0.

### Transwell invasion assays

Invasion of human or mouse cells were performed as described previously^37,66^. Fibroblasts or macrophages were seeded on permeable transwell inserts with 8 mm pores or BioCoat Matrigel Invasion Chamber (BD), and cell invasion was measured in the presence of 10% FBS complete medium. At 48 h, the polycarbonate filters with the invaded cells were stained with crystal violet and counted in ten randomly selected fields.

### Proteome profiler human cytokine array

The Proteome Profiler Human Cytokine Array kit (R&D Systems) was used. Conditioned medium was collected from scrambled (Scr) and shFOXF1 transfected HUVECs at 24 h after transfection.

### RNA-seq analysis

Endothelial cells (CD45^−^CD31^+^) were FACS-sorted from bleomycin-treated control *Foxf1*^*fl/fl*^ and end*Foxf1*^+/−^ mouse lungs 14 days after bleomycin treatment. RNA was extracted and sent for sequencing. Differentially expressed genes were identified using DESeq with fold change >2 and an adjusted *p* value (FDR) < 0.01. The *p* value was calculated using Z test (Pearson’s chi-square test) in the Agilent GeneSpring GX suite. For GSEA analysis, pre-ranked gene list generated through Biowardrobe software^67^ and analyzed using GSEA software available from Broad Institute^68^.

### Respiratory mechanics and arterial oxygenation

Mice were anesthetized on days 21 after first bleomycin administration. Measurements of the respiratory system mechanics were done using the flexiVent system (Scireq, Montreal, QC, Canada) as described^69^. Arterial oxygenation was measured using MouseOx + pulse oximeter (STARR Life Sciences) and analyzed using MouseOx Plus 1.6.X software. The MouseOx + photodiode sensor was placed on the shaved skin of the neck, and the measurements were taken for 10 min as described^49,69^. All data were analyzed using FlexiVent software (version 7.5).

### Nanoparticle generation and delivery of plasmid

The PBAEs were synthesized using two-step Aza-Michael addition synthesis. First, to generate the PBAE backbone, the Bisphenol A glycerolate diacrylate and 6-amino-1-hexanol (Sigma Aldrich) were dissolved in DMSO at a molar of 1.15 :1 for 23 hours at 90 °C. Next, the PBAE backbone was end-capped with PEI and modified using PDFO at 40 °C for 20 h. Before use, the PBAE polymer was lyophilized to remove the DMSO and dissolved in 25 mM HEPES buffer (pH = 7.4). To label the PBAE nanoparticle, the DyLight 650 NHS ester (ThermoFisher Scientific) was mixed with the nanoparticle at a mass ratio of 1: 100. *Foxf1* plasmid was generated by cloning DNA (3HA-RFP-Foxf) into the Enhanced Episomal Vectors (EEV) empty plasmid vectors (SBI). The PBAE polymer (300 µg) were used to encapsulate 40 µg of plasmid DNA (EEV-*Foxf1* or EEV-empty). The size distribution and the surface potential distribution were determined by Dynamic light scattering (DLS). Nanoparticles (250 µl) were delivered to mice via tail vein or eye vein on day 0 or day 7 after bleomycin administration.

### Statistics and reproducibility

Statistical significance differences in measured variables between experimental and control groups were assessed by Student’s *t* test (two-tailed), non-parametric Mann–Whitney U test or one-way ANOVA followed by Dunnett’s test. The two-way ANOVA test was used for two group comparison and multi-timepoint experiments. *P*-values <0.05 were considered significant. Values for all measurements were expressed as the mean ± standard deviation (SD). Statistical analysis was performed, and data were graphically displayed using GraphPad Prism vs 9.0 for Windows (GraphPad Software, Inc., San Diego, CA). All experiments in this study have been repeated independently with similar result more than 3 times.

### Reporting summary

Further information on research design is available in the Nature Portfolio Reporting Summary linked to this article.

## Supplementary information

Supplementary Information

Reporting Summary

## Data Availability

All the raw sequence data generated in this study is deposited in GEO database (accession number GSE213018). Single cell RNA sequencing data generated in this study from donor and IPF patient lungs (Supplementary Fig. S4) were deposited in GEO database (accession number GSE213017). Single cell RNA sequencing data generated in this study from bleomycin-treated and untreated adult mice lung were deposited in GEO database (accession number GSE213016). Single cell RNA sequencing data of Northwestern University (NW) datasets from donor and IPF lungs were retrieved from GEO database (GSE122960 https://www.ncbi.nlm.nih.gov/geo/). Bulk RNA sequencing data generated in this study from bleomycin-treated control and endFoxf1+/− mouse lung endothelial cells were also deposited in GEO database (GSM6578251 and GSM6578252). Source data are provided with this paper.

## Source data

Source Data
