# Supplementary material for: Lung endothelial cells regulate pulmonary fibrosis through FOXF1/R-Ras signaling
Source: Nat Commun. 2023 May 4;14:2560. doi: 10.1038/s41467-023-38177-2 (PMC10156846; doi:10.1038/s41467-023-38177-2)
Supplement: Supplementary file 1 — Supplementary Information [file 41467_2023_38177_MOESM1_ESM.pdf]

Bian et al., Supplemental Fig.S1

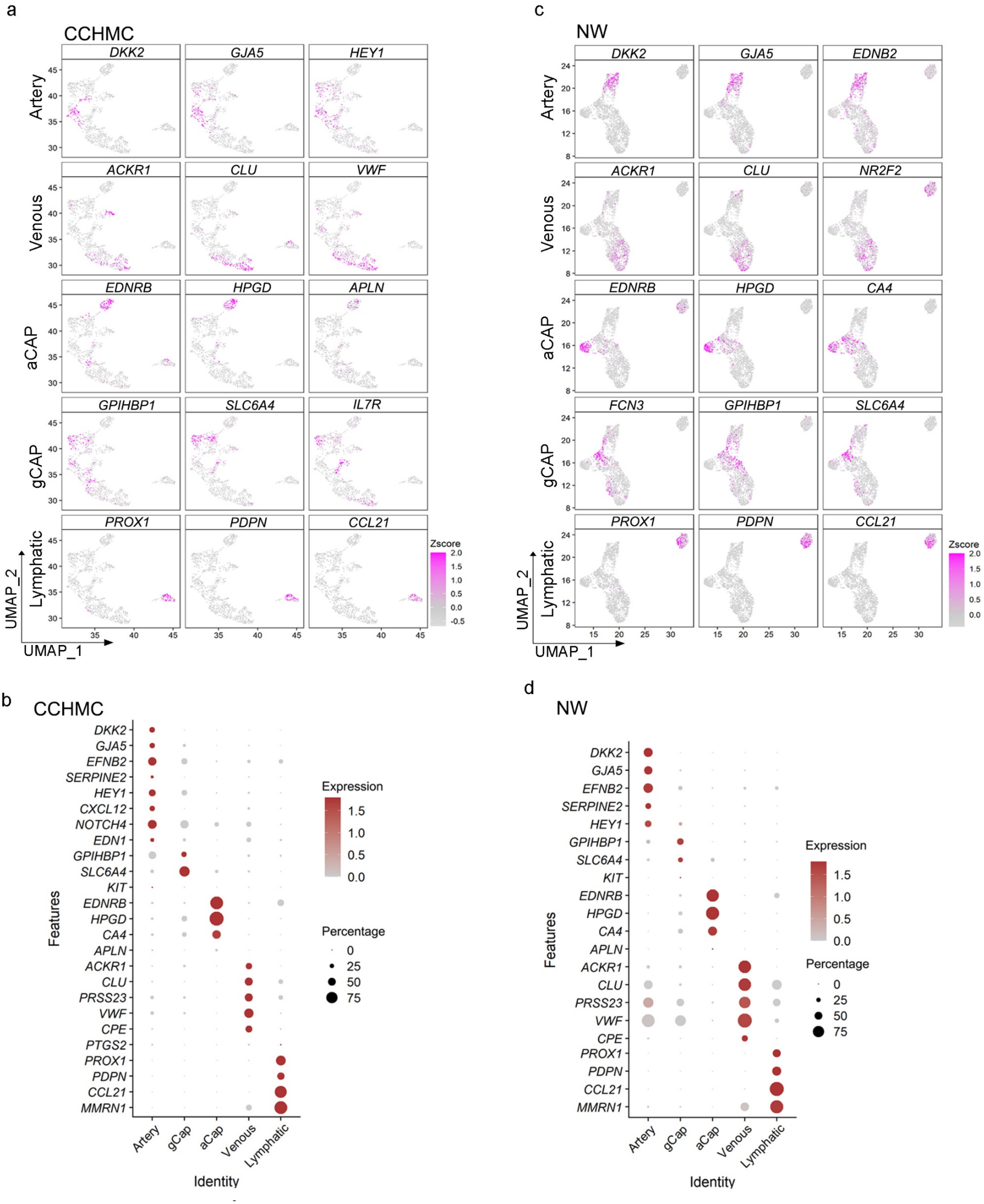

**Supplemental Figure S1. scRNA-seq analysis of human IPF and mouse fibrotic lungs. (a-b)** Sub-clustering of lung endothelial cells using scRNA-seq data from human lungs (CCHMC datasets) identifies five endothelial cells subclusters. Represented marker genes for endothelial subclusters are shown. **(c-d)** Sub-clustering of lung endothelial cells using downloaded scRNA-seq data (NW datasets, GSE 122960, PMID: 30554520). Represented marker genes for endothelial subclusters are shown.

Bian et al., Supplemental Fig.S2

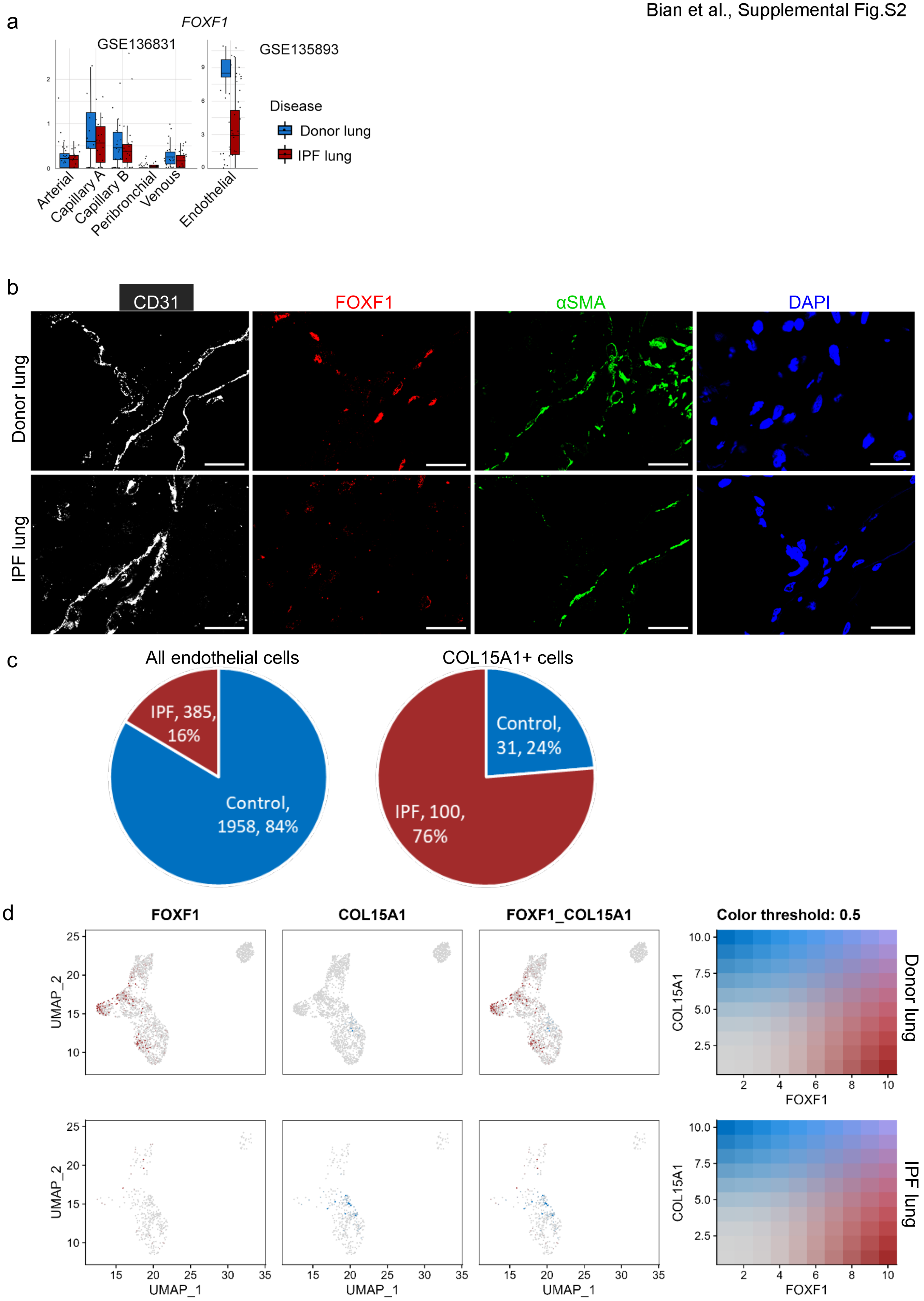

**Supplemental Figure S2. FOXF1 mRNA and protein levels are decreased in ECs of human IPF lung.** **(a)** *FOXF1* mRNA is decreased in endothelial cells of IPF lungs compared to endothelial cells of donor lungs. The boxplots showing the expression of *FOXF1* were generated using IPF Cell Atlas (http://www.ipfcellatlas.com/) using datasets from GSE136831[6] (left panel, control, n=29; IPF, n=32) and GSE135893[7] (right panel, control n=10; IPF, n=20). Boxplots representing expression distribution of FOXF1 in IPF vs control samples. The rectangle in the box plots represents 25%, 50%, and 75% quantiles with the black line inside representing the median. Each dot represents a single subject, and whiskers outside of the box represent 1.5 × interquartile range. **(b)** FOXF1 protein levels are decreased in ECs of human IPF lung. Lung sections from human IPF patients (n=5), normal lung donors (n=5) were stained with antibodies against FOXF1, CD31, αSMA and DAPI. Individual channels for each fluorescent antibody are showed. Bar = 20µm. **(c)** COL15A1+ endothelial cells are highly enriched in IPF endothelial cells. Left pie chart represents the proportion of all endothelial cells in IPF and control. The right pie chart represents the proportion of COL15A+ endothelial cells in IPF and Control. 76% of COL15A1+ cells are from IPF endothelial cells, which counts 16% of the integrated endothelial cells. Therefore, COL15A1+ cells are about 16.5-fold enriched in IPF endothelial cells. **(d)** Co-expression of *FOXF1* and *COL15A1* in IPF and Control ECs. Expression of *FOXF1* and *COL15A1* are showed using feature plots. Red color represents FOXF1+ cells and blue color represents COL15A1+ cells.

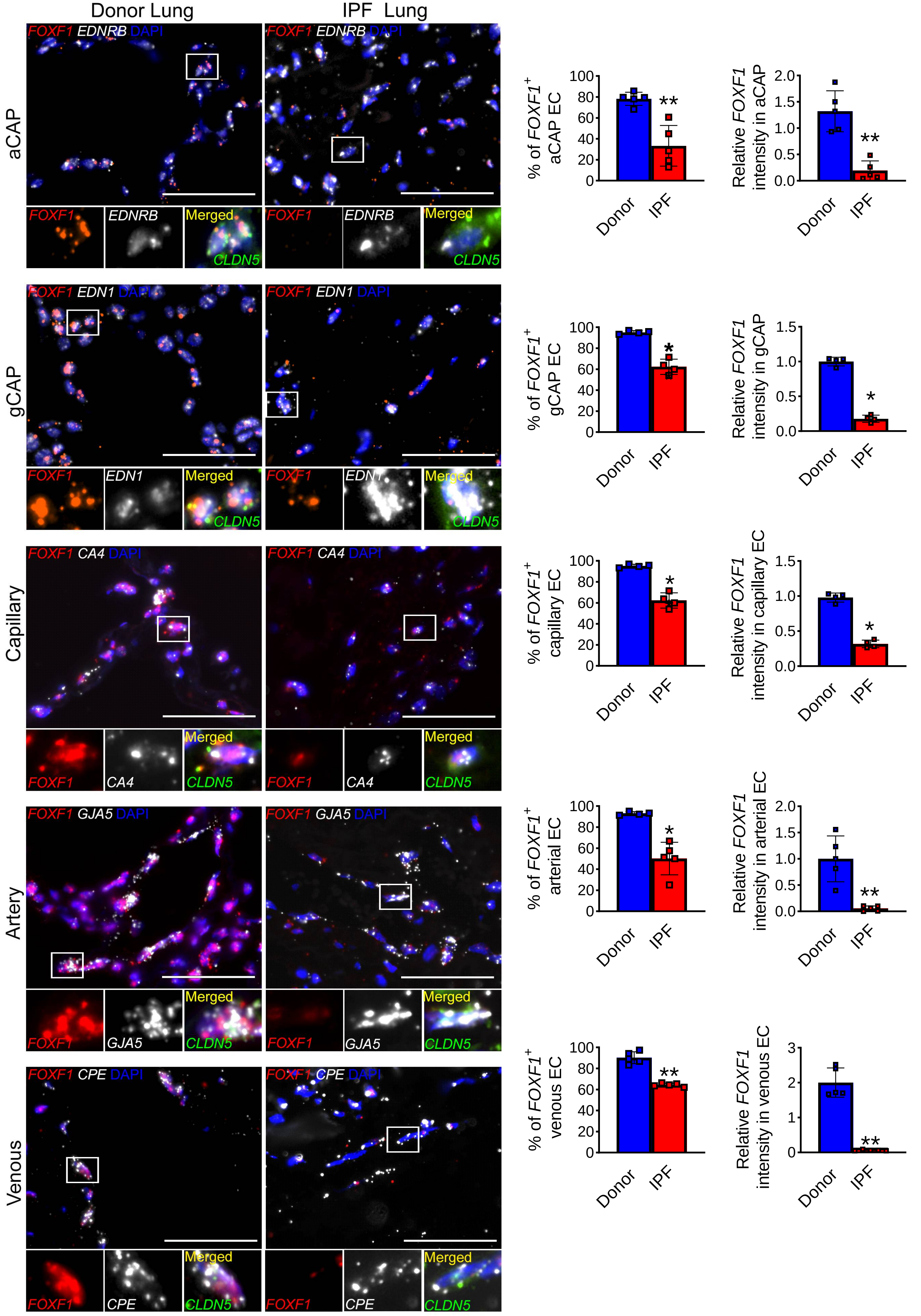
Donor Lung
IPF Lung
aCAP
gCAP
Capillary
Artery
Venous
FOXF1 EDNRB DAPI
FOXF1 EDN1 DAPI
FOXF1 CA4 DAPI
FOXF1 GJA5 DAPI
FOXF1 CPE DAPI
FOXF1
EDNRB
EDN1
CA4
GJA5
CPE
Merged
CLDN5
% of FOXF1+ aCAP EC
% of FOXF1+ gCAP EC
% of FOXF1+ capillary EC
% of FOXF1+ arterial EC
% of FOXF1+ venous EC
Relative FOXF1 intensity in aCAP
Relative FOXF1 intensity in gCAP
Relative FOXF1 intensity in capillary EC
Relative FOXF1 intensity in arterial EC
Relative FOXF1 intensity in venous EC
Donor
IPF
**
*

**Supplemental Figure S3.** ***FOXF1*** **mRNA is decreased in aCAP, gCAP, arterial, and venous endothelial cells in human IPF lungs.** RNA in situ hybridization using donor and IPF lung tissue section shows *FOXF1* transcripts (red) localized in aCAP, gCAP, arterial and venous endothelial cells of the donor lungs and decreased in the in aCAP, gCAP, arterial and venous endothelial cells of the IPF lungs compared to donor lungs. Bar = 50µm. Percentage of FOXF1+ cells within each type of lung endothelial cells was counted in 5-10 random fields per lung and presented as mean ± SD (Donor and IPF aCAP, n=5; Donor and IPF gCAP, n=4; Donor and IPF Capillary, n=4; %of FOXF1$^+$ arterial EC in Donor, n=4; % of FOXF1$^+$ arterial EC in IPF, n=5; Donor and IPF arterial EC FOXF1 intensity, n=5; Donor and IPF Venous EC, n=5). Relative FOXF1 intensity was quantified using Nikon NIE CIC Analysis Elements software. *$p < 0.05$, **$p < 0.01$ by Mann-Whitney two-tailed test. Source data are provided as a Source Data file.

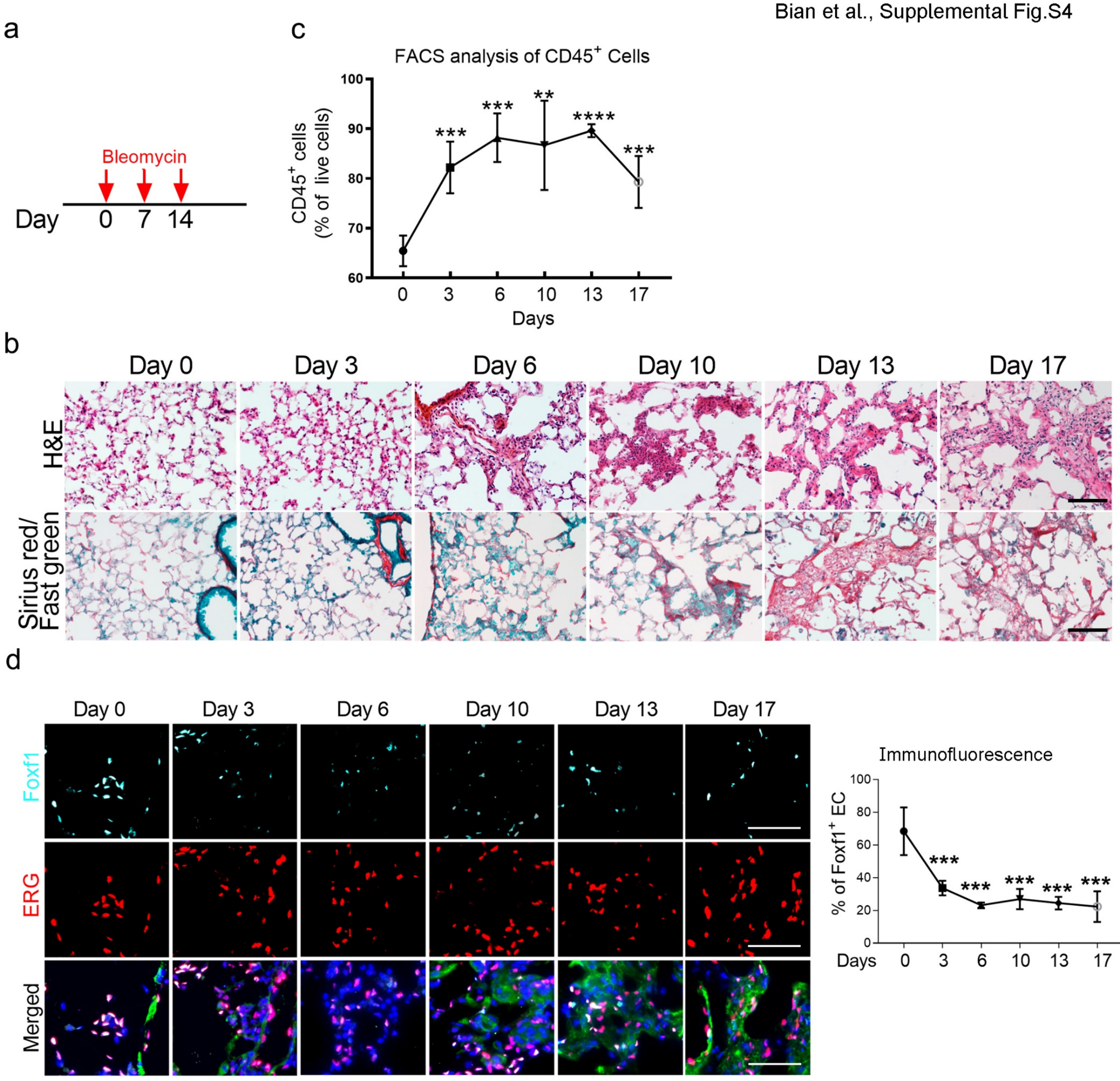
a
Bleomycin
Day 0 7 14
c
FACS analysis of CD45+ Cells
CD45+ cells
(% of live cells)
Days
b
Day 0
Day 3
Day 6
Day 10
Day 13
Day 17
H&E
Sirius red/
Fast green
d
Foxf1
ERG
Merged
Immunofluorescence
% of Foxf1+ EC
Days

**Supplemental Figure S4. Progression of pulmonary fibrosis in mouse lungs after multiple bleomycin administrations.** **(a)** Schematic diagram of multiple bleomycin IT administrations to induce chronic pulmonary fibrosis in mice. **(b)** Progression of lung fibrosis in mouse lungs is shown by H&E and Sirius red / fast green staining. Sirius red binds to all types of collagens, fast green stains non-collagenous proteins. Lungs from bleomycin-treated mice were harvested on days 3, 6, 10, 13 and 17. Bar = 100µm. **(c)** Increased accumulation of $CD45^+$ cells in the lungs of bleomycin-treated mice is shown using flow cytometry (Day 0, 3, 6, 10, 17 n=4; Day 13, n=7 mice per group). **(d)** Time-dependent decrease in the percentage of $FOXF1^+$ endothelial cells among total lung endothelial cells after chronic bleomycin treatment is shown using immunofluorescence for FOXF1 (light blue), endothelial cell marker ERG (red), aSMA (Green). DAPI (Blue) was used to counterstain cell nuclei. Bar = 50µm. $FOXF1^+$/$CD31^+$ double positive cells were counted in 5 random fields and presented as mean ± SD (Day 0, 6, n=3; Day 3, 17, n=4; Day10, n=7; Day 13, n=5 mice per group). ***$p<0.001$ by one-way ANOVA followed by Dunnett's test. Source data are provided as a Source Data file.

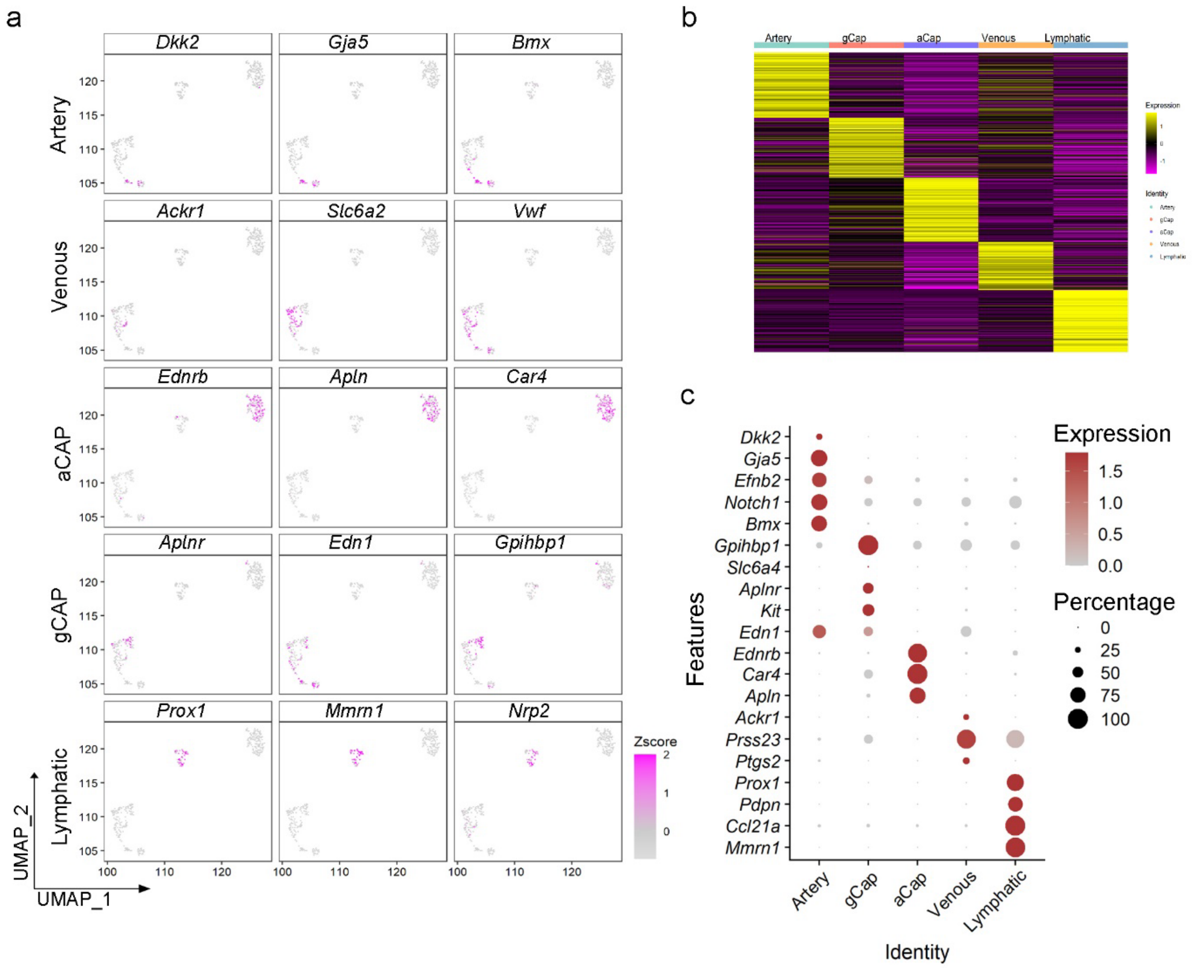


**Supplemental Figure S5. scRNA-seq analysis of mouse fibrotic lungs. (a)** Sub-clustering of lung endothelial cells using scRNA-seq data from mouse lungs identifies five endothelial cell subclusters. Represented marker genes for endothelial subcluster genes are shown. **(b)** Heatmap shows differentially expressed genes in the defined endothelial clusters. Gene expression was log normalized. **(c)** Dot-plot shows mRNA expression of genes used to subcluster lung endothelial cells.

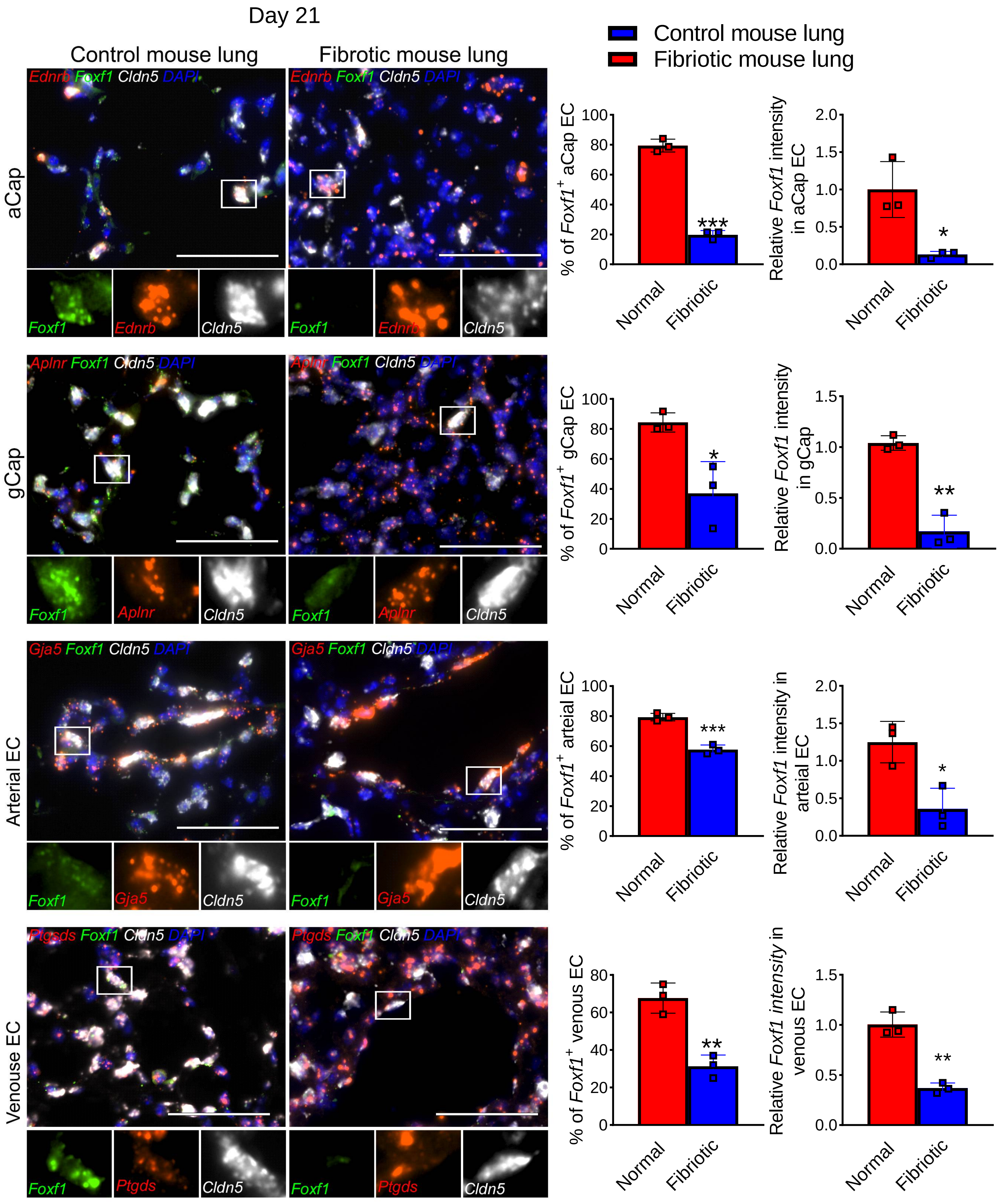
Day 21
Control mouse lung
Fibrotic mouse lung
Control mouse lung
Fibriotic mouse lung
aCap
gCap
Arterial EC
Venouse EC
Ednrb Foxf1 Cldn5 DAPI
Aplnr Foxf1 Cldn5 DAPI
Gja5 Foxf1 Cldn5 DAPI
Ptgsds Foxf1 Cldn5 DAPI
Ptgds Foxf1 Cldn5 DAPI
Foxf1
Ednrb
Aplnr
Gja5
Ptgds
Cldn5
% of Foxf1+ aCap EC
% of Foxf1+ gCap EC
% of Foxf1+ arteial EC
% of Foxf1+ venous EC
Relative Foxf1 intensity in aCap EC
Relative Foxf1 intensity in gCap
Relative Foxf1 intensity in arteial EC
Relative Foxf1 intensity in venous EC
Normal
Fibriotic
***
**
*

**Supplemental Figure S6. *Foxf1* is decreased in aCAP, gCAP, arterial, and venous endothelial cells in bleomycin-treated mouse lungs.** RNA in situ hybridization using mouse lung tissue sections at day 21 after first bleomycin injury shows *Foxf1* transcripts (green) localized in endothelial cells of the control mouse lungs and decreased in the in aCAP, gCAP, arterial and venous endothelial cells of the fibrotic mouse lungs. Bar = 50μm. Percentage of *Foxf1*+ cells within each type of lung endothelial cells was counted in 5 random fields per lung and presented as mean ± SD (n= 3 mice per group). Relative Foxf1 intensity was quantified using Nikon NIE CIC Analysis Elements software. ***p < 0.001 by Student's T-test (two tailed). Source data are provided as a Source Data file.

Bian et al., Supplemental Fig.S7

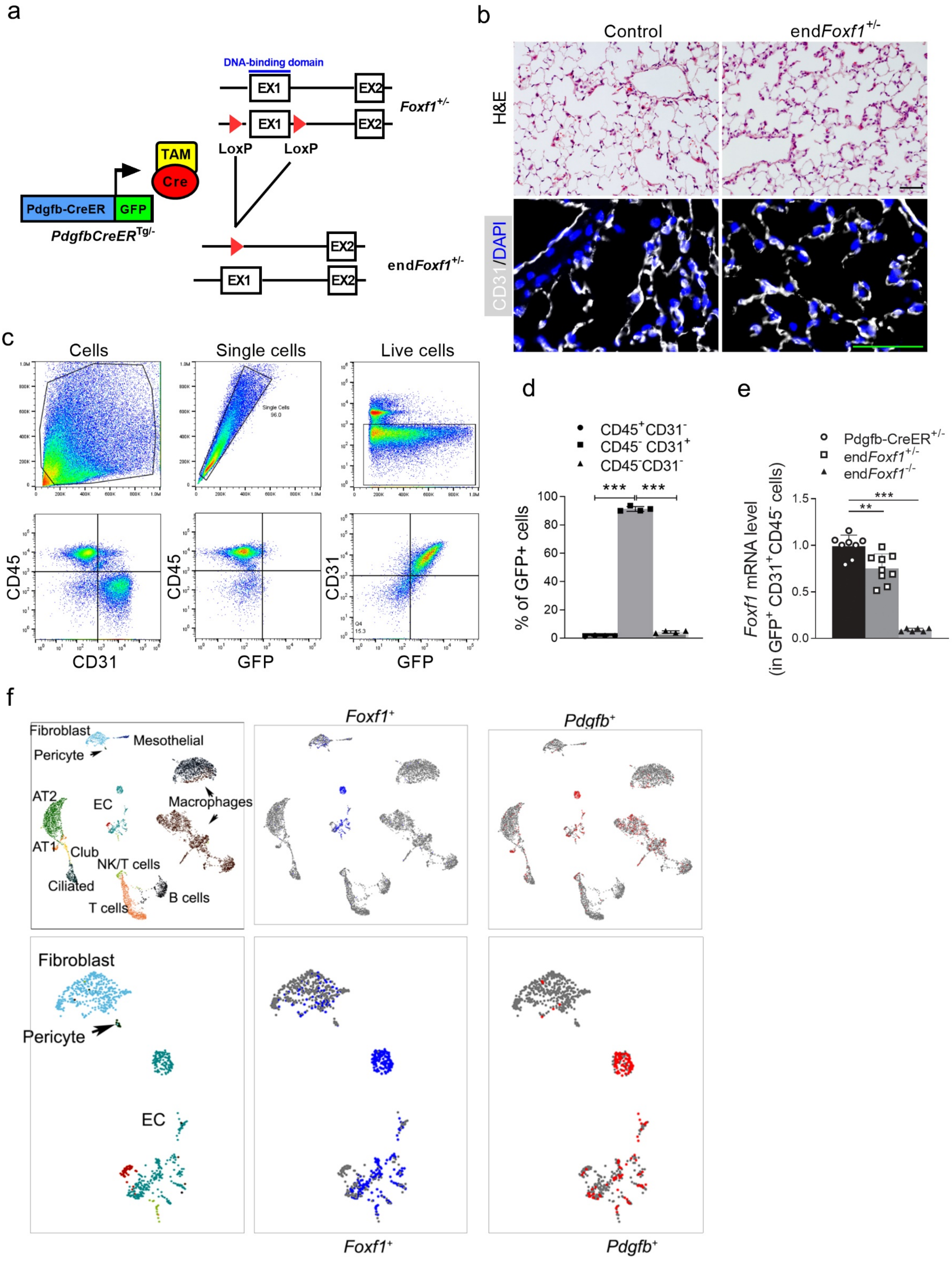

**Supplemental Figure S7. Endothelial-specific deletion of *Foxf1* in mice. (a)** Schematic of *Foxf1* deletion in *Pdgfb*-CreER;*Foxf1*$^{fl/+}$ (end*Foxf1*$^{+/-}$) transgenic mice. **(b)** *Pdgfb*-CreER;*Foxf1*$^{fl/+}$ (end*Foxf1*$^{+/-}$) transgenic mice demonstrate normal lung structure shown using H&E and immunostaining for CD31 (white). DAPI (blue) is used to visualize the nuclei. Bar = 50μm. **(c)** Gate setting used to FACS-sort CD45-GFP+ endothelial cells. **(d)** Percentage of GFP+ cells among immune cells (CD45+CD31-), endothelial cells (CD45 -CD31+) and CD45-CD31- cells in total live cells (n=4 mice per group). **(e)** *Foxf1 mRNA* is decreased in FACS-sorted GFP+ endothelial cells from end*Foxf1*$^{+/-}$ and end*Foxf1*$^{-/-}$ mouse lungs compared to control lungs in untreated mice. *Actb* mRNA was used for normalization. Pdgfb-CreER$^{+/-}$, n=8; end*Foxf1*$^{+/-}$, n=9; end*Foxf1*$^{-/-}$, n=7. Data presented as mean ± SD. **$p < 0.01$, ***$p < 0.001$ by Student's T test (two tailed). **(f)** scRNA-seq data were generated from pooled control (n=4) and bleomycin treated (n=6) mouse lungs at day 21 after bleomycin injury. Cells were visualized by uniform manifold approximation and projection (UMAP) and colored by subtypes. UMAP plots show *Foxf1* (blue) and *Pdgfb* (red) expression in the normal and fibrotic mouse lungs after Z-score normalization. Source data are provided as a Source Data file.

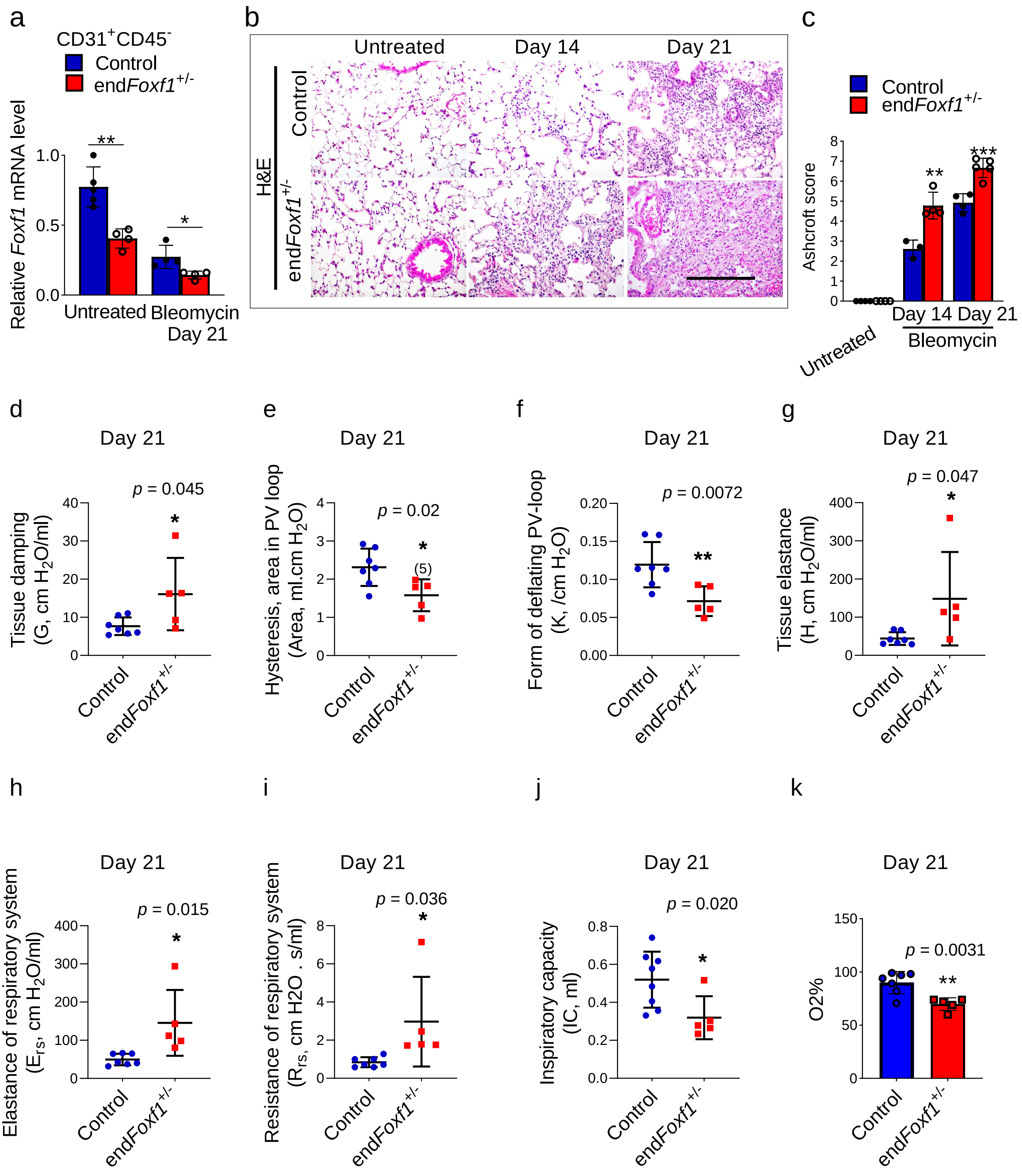
a
CD31+CD45-
Control
endFoxf1+/-
Relative Foxf1 mRNA level
Untreated
Bleomycin Day 21
b
Untreated
Day 14
Day 21
H&E
Control
endFoxf1+/-
c
Control
endFoxf1+/-
Ashcroft score
Untreated
Day 14
Day 21
Bleomycin
d
Day 21
p = 0.045
Tissue damping (G, cm H2O/ml)
Control
endFoxf1+/-
e
Day 21
p = 0.02
(5)
Hysteresis, area in PV loop (Area, ml.cm H2O)
f
Day 21
p = 0.0072
Form of deflating PV-loop (K, /cm H2O)
g
Day 21
p = 0.047
Tissue elastance (H, cm H2O/ml)
h
Day 21
p = 0.015
Elastance of respiratory system (Ers, cm H2O/ml)
i
Day 21
p = 0.036
Resistance of respiratory system (Rrs, cm H2O . s/ml)
j
Day 21
p = 0.020
Inspiratory capacity (IC, ml)
k
Day 21
p = 0.0031
O2%

**Supplemental Figure S8. Deletion of FOXF1 in endothelial cells increased severity of pulmonary fibrosis in mice.** Mice were treated with bleomycin on days 0, 7 and 14. **(a)** *Foxf1 mRNA* is decreased in FACS-sorted endothelial cells of end*Foxf1*$^{+/-}$ mouse lungs compared to control lungs in both untreated and bleomycin treated mice. *Actb* mRNA was used for normalization (Untreated Control, n=5; Untreated end*Foxf1*$^{+/-}$, n=4; **p=0.0022; Bleomycin Control, and end*Foxf1*$^{+/-}$, n=4; **p* = 0.0242). **(b)** H&E staining of control and endFoxf1$^{+/-}$ mouse lungs after bleomycin treatment. Bar = 50µm. **(c)** Increased Ashcroft score in fibrotic end*Foxf1*$^{+/-}$ mouse lungs compared to control fibrotic lungs at day 14 and day 21 after first bleomycin administration (Untreated Control and end*Foxf1*$^{+/-}$, n=4; Day 14 Control, n=3; Day 14 end*Foxf1*$^{+/-}$, n=4; ***p* = 0.0048; Day 21 Control, n=4; Day 14 end*Foxf1*$^{+/-}$, n=5; ****p* = 0.0009). **(d-j)** Lung functions were decreased in fibrotic end*Foxf1*$^{+/-}$ mouse lungs compared to control fibrotic lungs at day 21 after bleomycin administration. Lung functions were measured using FlexiVent system. (*d-i*), Control, n=7; end*Foxf1*$^{+/-}$, n=5. **p* = 0.045 (*d*), 0.02 (*e*), 0.0072 (*f*), 0.047 (*g*), 0.015 (*h*), 0.036(*i*). (*j*) Control, n=8; end*Foxf1*$^{+/-}$, n=5. **p* = 0.02. (k) Oxygen saturation decreased in endFoxf1$^{+/-}$ mice (Control, n=7; end*Foxf1*$^{+/-}$, n=5. ***p* = 0.0031 (*k*). Data presented as mean ± SD. **p* < 0.05, ***p* < 0.01, ****p* < 0.001 by Student's T test (two tailed). Source data are provided as a Source Data file.

Bian et al., Supplemental Fig. S9

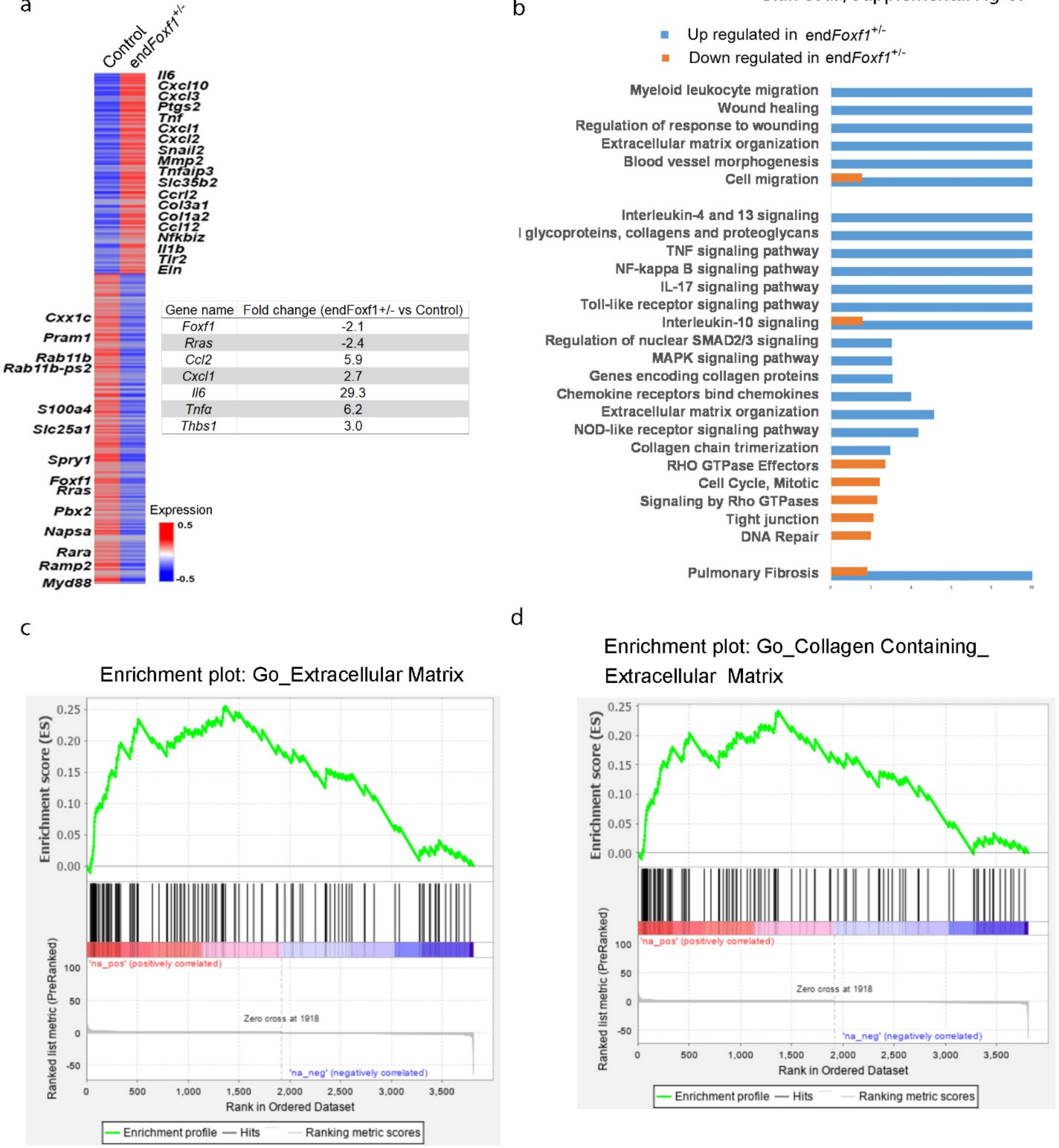


**Supplemental Figure S9. RNA-seq analysis using FACS-sorted endothelial cells from bleomycin-treated control and end*Foxf1*$^{+/-}$ lungs.** Endothelial cells ($CD45^{-}CD31^{+}$) from bleomycin-treated end*Foxf1*$^{+/-}$ and control mice were FACS-sorted on day 14 after first bleomycin

treatment. RNA was extracted and used for RNA-seq. **(a)** Heatmap shows top differentially expressed genes in endothelial cells from bleomycin-treated control and bleomycin-treated end*Foxf1*$^{+/-}$ mice. Fold changes of *Foxf1, Rras, Ccl2, Cxcl1 Il6, Tnfα* and *Thbs1* mRNAs were shows in the table. **(b)** Gene Set Enrichment Analysis (GSEA) shows the most enriched functional categories in endothelial cells. **(c-d)** “Extracellular matrix” and “Collagen containing extracellular matrix” were enriched in end*Foxf1*$^{+/-}$ endothelial cells by GSEA enrichment analysis.

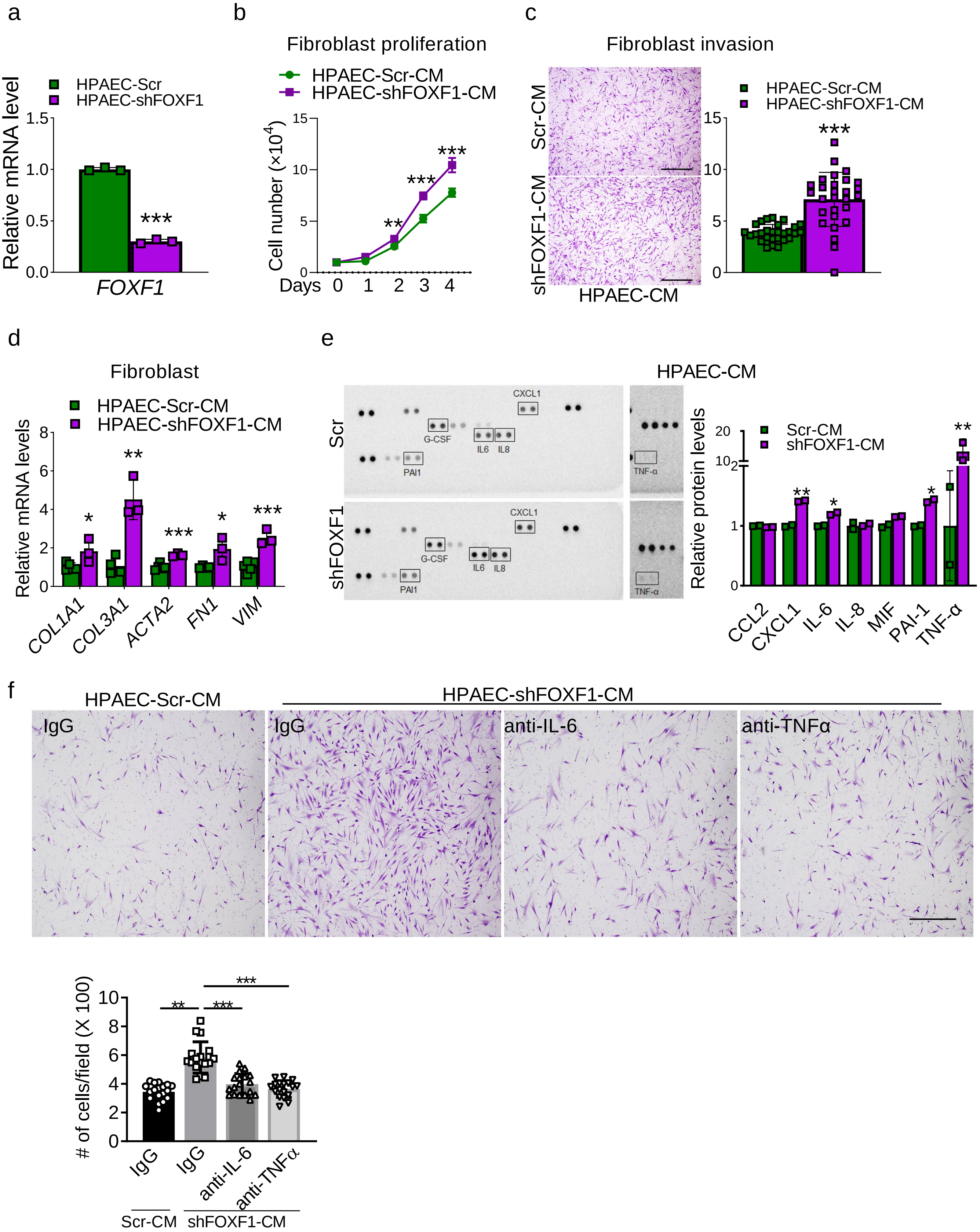
a
HPAEC-Scr
HPAEC-shFOXF1
Relative mRNA level
1.5
1.0
0.5
0.0
***
FOXF1
b
Fibroblast proliferation
HPAEC-Scr-CM
HPAEC-shFOXF1-CM
Cell number (×10⁴)
15
10
5
0
**
***
***
Days 0 1 2 3 4
c
Fibroblast invasion
Scr-CM
shFOXF1-CM
HPAEC-CM
HPAEC-Scr-CM
HPAEC-shFOXF1-CM
15
10
5
0
***
d
Fibroblast
HPAEC-Scr-CM
HPAEC-shFOXF1-CM
Relative mRNA levels
8
6
4
2
0
*
**
***
*
***
COL1A1
COL3A1
ACTA2
FN1
VIM
e
HPAEC-CM
Scr
shFOXF1
CXCL1
G-CSF
IL6
IL8
PAI1
TNF-α
Relative protein levels
20
10
2
1
0
Scr-CM
shFOXF1-CM
**
*
*
**
CCL2
CXCL1
IL-6
IL-8
MIF
PAI-1
TNF-α
f
HPAEC-Scr-CM
HPAEC-shFOXF1-CM
IgG
IgG
anti-IL-6
anti-TNFα
# of cells/field (X 100)
10
8
6
4
2
0
**
***
***
IgG
IgG
anti-IL-6
anti-TNFα
Scr-CM
shFOXF1-CM

**Supplemental Figure S10. FOXF1-deficient HPAEC endothelial cells increase myofibroblast activation.** **(a)** Efficient inhibition of *FOXF1* mRNA in shFOXF1-transfected HPAEC cells is shown with qRT-PCR. *ACTB* mRNA was used for normalization. (n=3 samples per group). **(b)** Conditioned media (CM) from FOXF1-deficient HPAEC increases fibroblast proliferation. CCD-19Lu fibroblasts were cultured in the presence of CM from control or FOXF1-deficient HPAEC. N=6 samples per group. **(c)** CM from FOXF1-deficient HPAEC increases invasion of cultured CCD-19Lu fibroblasts. Human CCD-19Lu fibroblasts were seeded on the insert of transwell chamber coated with matrigel in the presence of CM from scrambled control (Scr-CM) or FOXF1-deficient (shFOXF1-CM) HPAEC. Bar = 50μm. Graph represents average numbers of invaded cells per field (n=6 samples per group). **(d)** CCD-19Lu fibroblasts cultured in CM from FOXF1-deficient HPAEC had increased expression of pro-fibrotic genes compared to fibroblasts cultured in CM from control HPAEC as shown by qRT-PCR (n=6 samples per group). **(e)** CM from FOXF1-deficient HPAEC had increased levels of pro-inflammatory mediators as determined by Proteome Profiler Human Cytokine Array (n=2 samples pooled from 4 biological independent samples). **(f)** Inhibition of IL-6 and TNFα using blocking antibodies attenuated CCD-19Lu fibroblast invasion in the presence of CM from FOXF1-deficient HPAEC (n=6 samples per group). Bar = 50μm. Data presented as mean ± SD. *p<0.05, **p<0.01, ***p<0.001 by student's t test (two tailed). CM, conditioned medium. Source data are provided as a Source Data file.

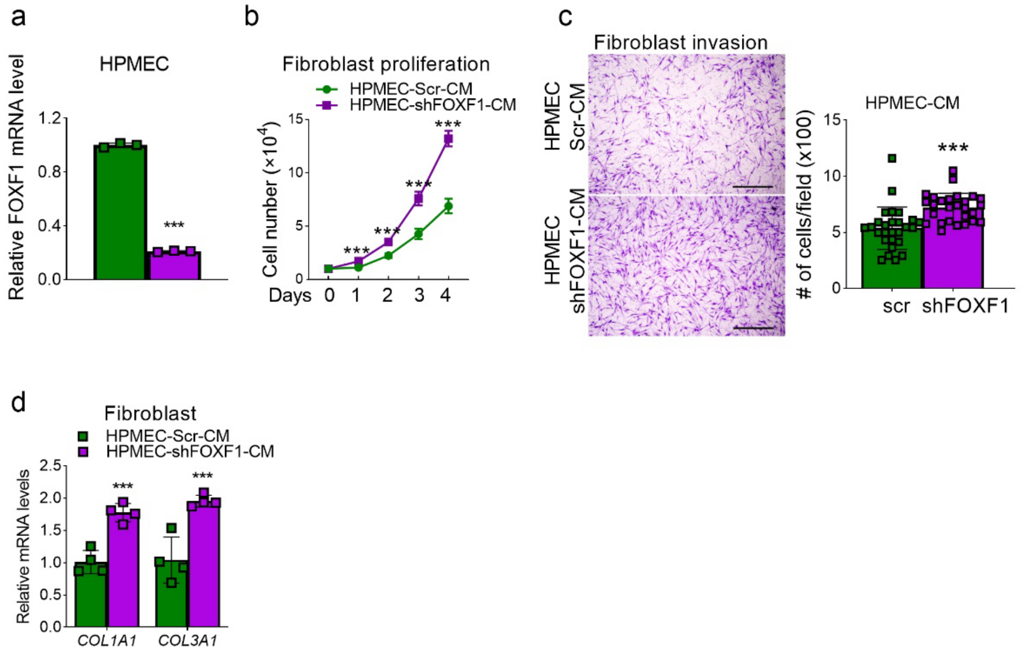


**Supplemental Figure S11. FOXF1-deficient HPMEC endothelial cells increase myofibroblast activation.** **(a)** Efficient inhibition of *FOXF1* mRNA in shFOXF1-transfected HPMEC cells is shown with qRT-PCR. *ACTB* mRNA was used for normalization (n=3 per group). **(b)** CM from FOXF1-deficient HPMEC increases fibroblast proliferation. CCD-19Lu fibroblasts were cultured in the presence of CM from control or FOXF1-deficient HPMEC. (n=6 per group). **(c)** Conditioned media (CM) from FOXF1-deficient HPMEC increases invasion of cultured CCD-19Lu fibroblasts. Human CCD-19Lu fibroblasts were seeded on the insert of transwell chamber coated with matrigel in the presence of CM from control (Scr-CM) or FOXF1-deficient (shFOXF1-CM) HPMEC. Bar = 50μm. Graph represents average numbers of invaded cells per field. N=6 samples per group. **(d)** CCD-19Lu fibroblasts cultured in CM from FOXF1-deficient HPMEC had increased expression of pro-fibrotic genes compared to fibroblasts cultured in CM from control HPMEC as shown by qRT-PCR (n=4 samples per group). Data presented as mean ± SD. *p<0.05, **p<0.01, ***p<0.001 by student's t test (two tailed). CM, conditioned medium. Source data are provided as a Source Data file.

Bian et al., Supplemental Fig.S12

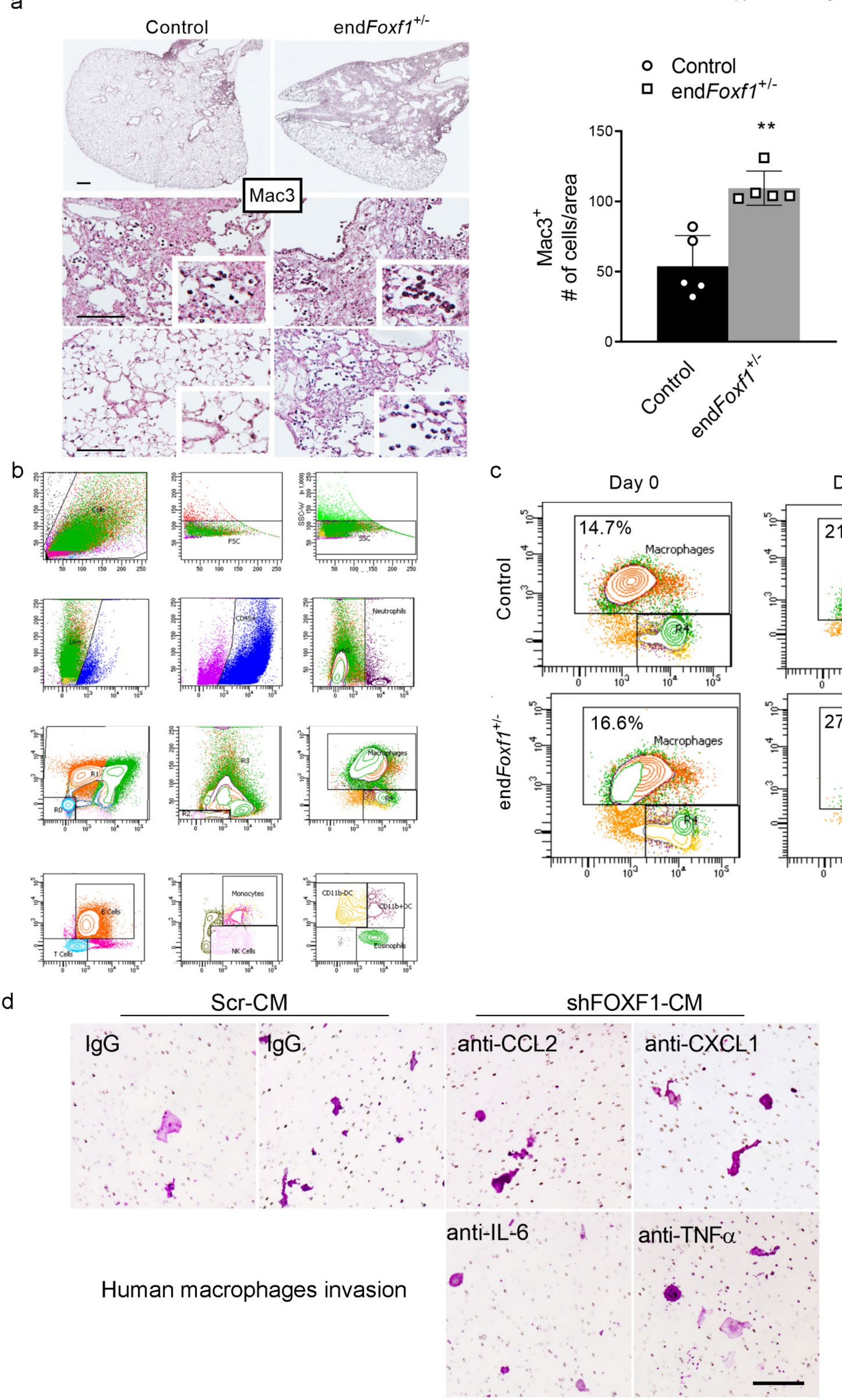

**Supplemental Figure S12. Deficiency of FOXF1 in EC increases the number of macrophages in the fibrotic lungs. (a)** Immunostaining shows the increased number of macrophages in bleomycin-treated end*Foxf1*$^{+/-}$ mice lungs. Control and end*Foxf1*$^{+/-}$ mouse lungs were stained with antibodies against Mac3 at day 21 after bleomycin administration. Mac3-positive macrophages were quantified in 10 random fields (n=5 mice per group). Bar = 500µm. Data presented as mean ± SEM. **p<0.01 by Student's T-test two tailed). Source data are provided as a Source Data file. **(b)** Gating strategy to quantify the immune cells using flow cytometry analysis. **(c)** Representative dot-plots of FACS analysis showed an increase in the percentage of macrophages in bleomycin-treated end*Foxf1*$^{+/-}$ mice lungs compared to control bleomycin-treated lungs. **(d)** Inhibition of CCL2, CXCL12, IL-6 and TNFα in CM from FOXF1-depleted HUVECs (sh*FOXF1*-CM) using blocking antibodies attenuated microphage invasion. Human primary macrophages were seeded on the insert of transwell chamber coated with matrigel and incubated in CM from scrambled control shRNA (Scr-CM) or sh*FOXF1*-transfected HUVEC cells. Bar = 200µm.

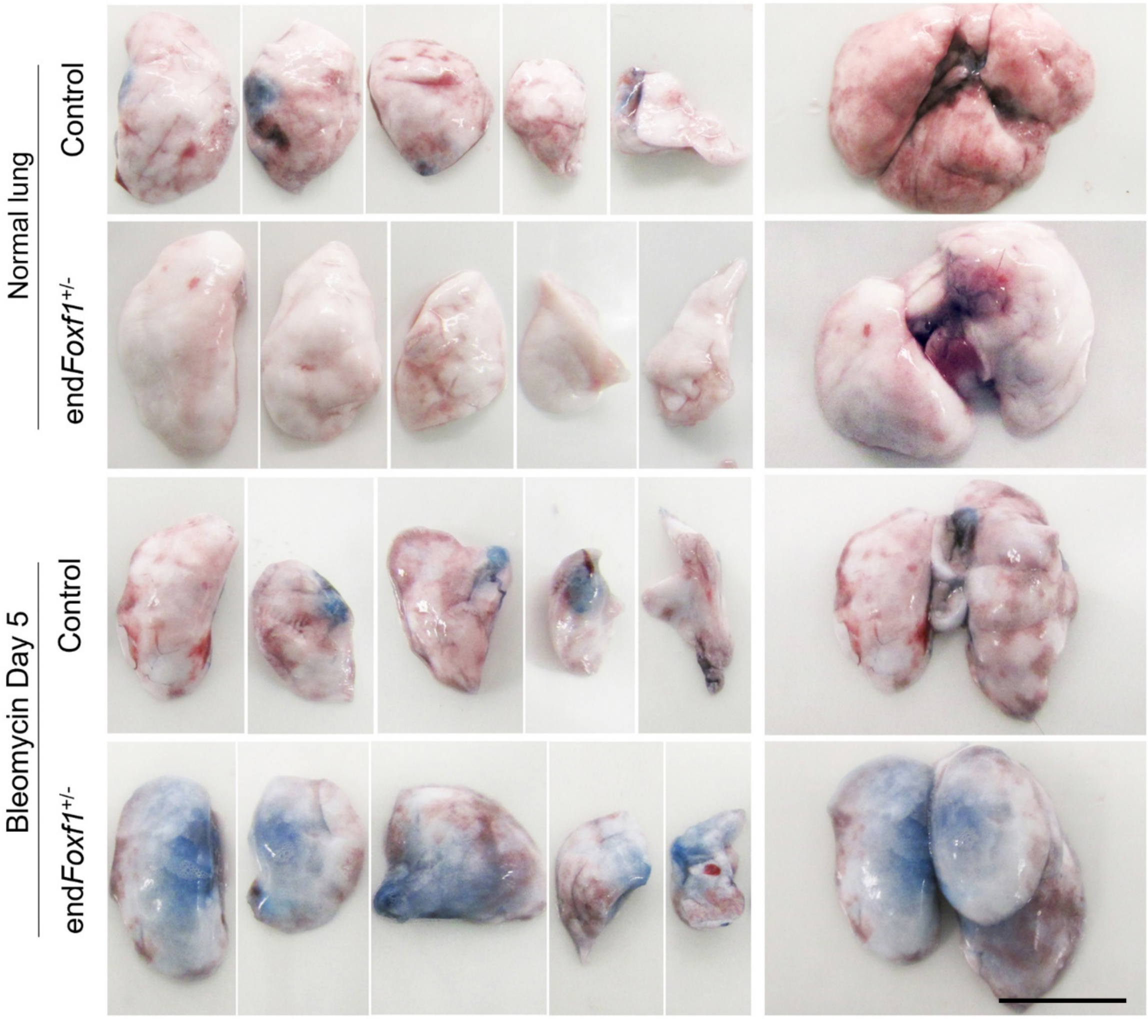


**Supplemental Figure S13. Deletion of Foxf1 in endothelial cells increased endothelial permeability in mice.** Bleomycin-treated end*Foxf1*$^{+/-}$ lungs displayed increased endothelial permeability compared to bleomycin-treated control lungs as determined by Evans blue dye. Mouse lungs were harvested on day 5 after bleomycin administration. Bar = 1cm. n=3 mice per group.

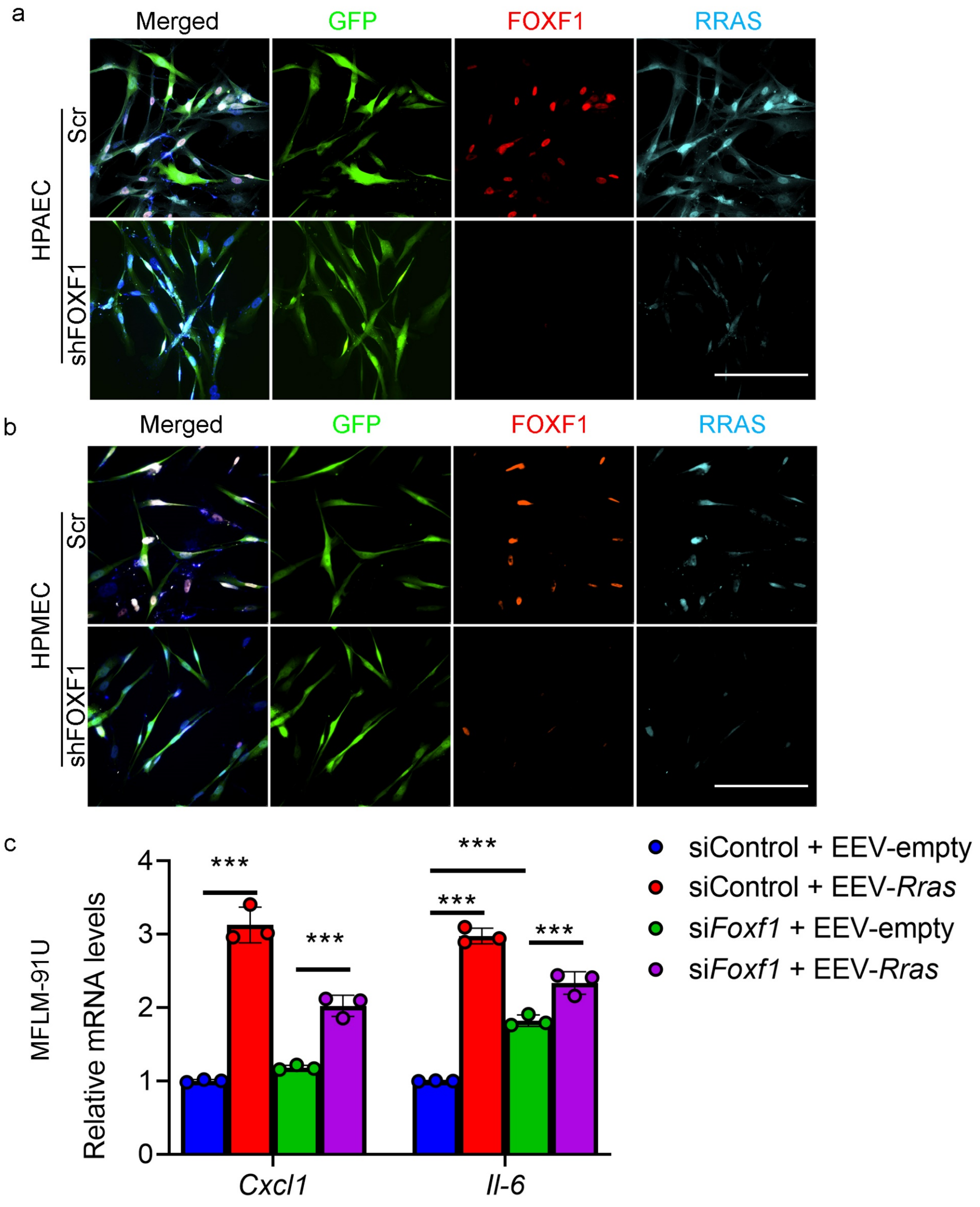
a
Merged
GFP
FOXF1
RRAS
HPAEC
Scr
shFOXF1
b
Merged
GFP
FOXF1
RRAS
HPMEC
Scr
shFOXF1
c
MFLM-91U
Relative mRNA levels
0
1
2
3
4
***
***
***
***
***
Cxcl1
Il-6
siControl + EEV-empty
siControl + EEV-Rras
siFoxf1 + EEV-empty
siFoxf1 + EEV-Rras

**Supplemental Figure S14. Over-expression of R-Ras in Foxf1-deficient endothelial cells in *vitro*. (a)** Co-localization of FOXF1 (red) and RRAS (light blue) in shRNA transduced HPAECs (GFP+ cells) and counterstained with DAPI (dark blue). HPAEC cells were transduced with TRC Lentiviral construct (GFP+ cells) to knockdown *FOXF1* (shFOXF1) or control non-targeting shRNA (Scr). Bar = 200μm. **(b)** Co-localization of FOXF1 (red) and RRAS (light blue) in shRNA transduced HPMECs (GFP+ cells) and counterstained with DAPI (dark blue). HPAEC cells were transduced with TRC Lentiviral construct (GFP+ cells) to knockdown *FOXF1* (shFOXF1) or control non-targeting shRNA (Scr). Bar = 200μm. **(c)** Overexpression of R-Ras has no effect on expression of *Cxcl1* and *IL-6* in FOXF1-deficient endothelial cells. Overexpression of R-Ras increased *Cxcl1* and *IL-6* mRNAs in mock-transfected cells as shown by qRT-PCR. MFLM-91U cells were co-transfected with non-targeting siRNA (siControl) or si*Foxf1*, and CMV-empty vector or CMV-*Rras*. *Actb* mRNA was used for normalization (n=3). Data presented as mean ± SEM. ***$p < 0.001$ by Student's t test (two tailed). Source data are provided as a Source Data file.

Bian et al., Supplemental Fig.S15

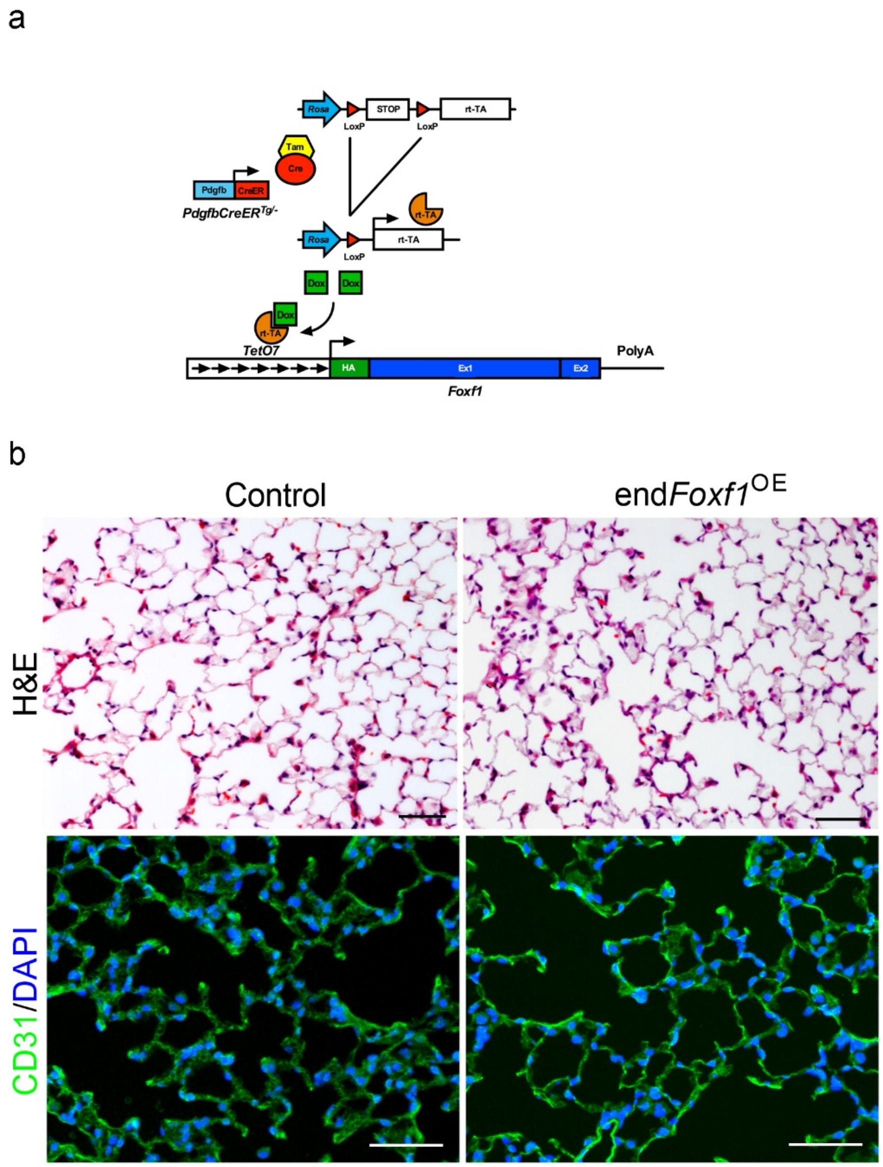


**Supplemental Figure S15. Over-expression of *Foxf1* transgene in endothelial cells does not change lung morphology in quiescent mouse lungs. (a)** Schematic diagram shows activation of *Foxf1* transgene in endothelial cells using *Pdgfb-CreER*$^{tg/+}$*;* LSL-rtTA$^{tg/+}$; TetO-*Foxf1*$^{tg/+}$ transgenic mice (*endFoxf1*$^{OE}$). Abbreviations: rt-TA, reverse tetracycline activator; TetO7, CMV promoter containing 7 tetracycline operator binding sites; Dox, doxycycline; Tam, tamoxifen; Ex, exon. **(b)** *endFoxf1*$^{OE}$ transgenic mice show normal lung structure by H&E and immunostaining for CD31 (green). DAPI (blue) is used to visualize nuclei. Bar = 50μm.

Supplemental fig.S16

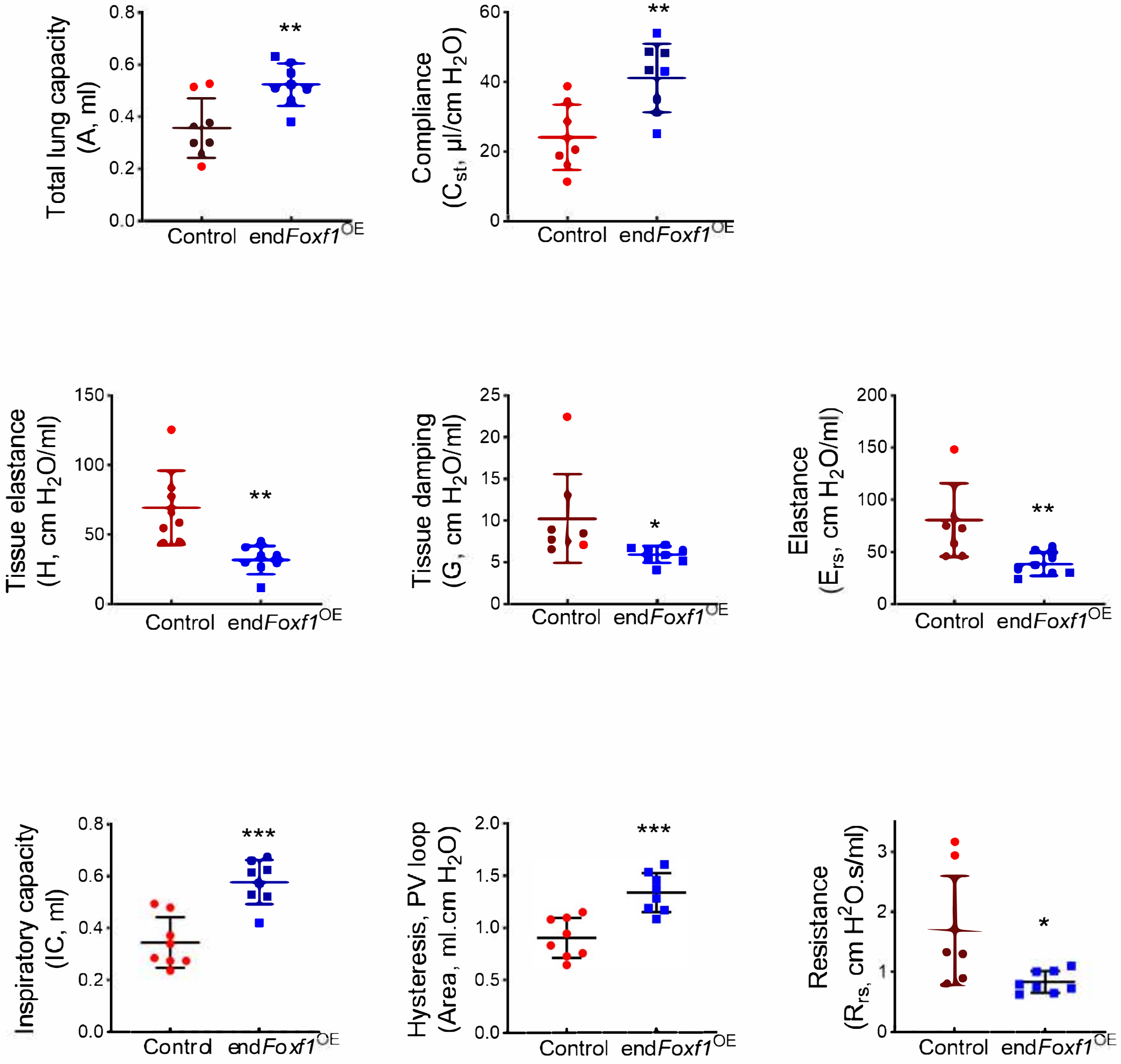

**Supplemental Figure S16. Over-expression of *Foxf1* in endothelial cells on day 10 after first bleomycin administration improves lung functions.** Mice were harvested on D21 after the first bleomycin administration. Lung functions were measured using FlexiVent system (n=8 per group). Data presented as mean ± SD. *p<0.05, **p<0.01, ***p<0.001 by student's t test (two tailed). Source data are provided as a Source Data file.

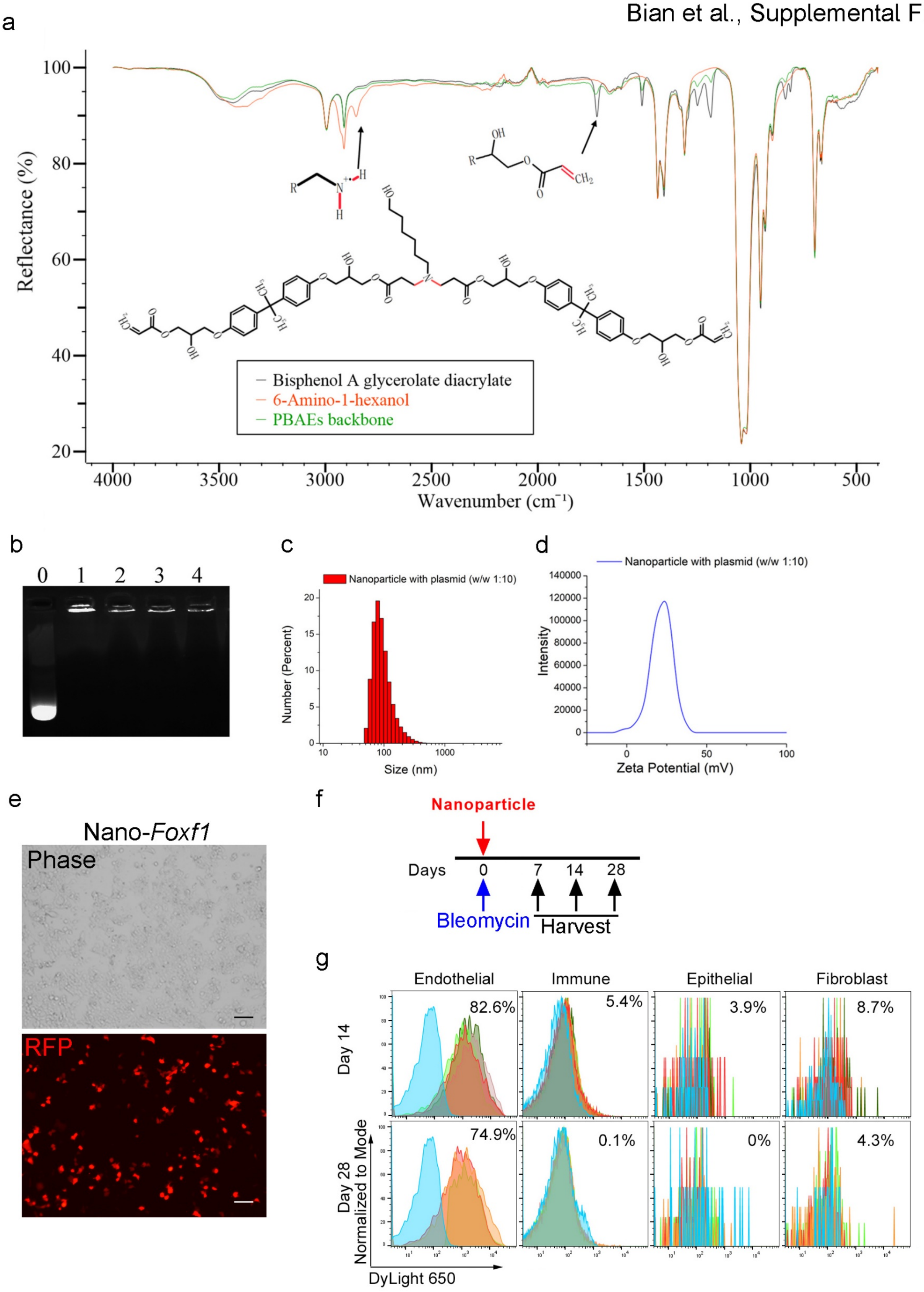
a
Reflectance (%)
Wavenumber (cm⁻¹)
Bisphenol A glycerolate diacrylate
6-Amino-1-hexanol
PBAEs backbone
b
0 1 2 3 4
c
Nanoparticle with plasmid (w/w 1:10)
Number (Percent)
Size (nm)
d
Nanoparticle with plasmid (w/w 1:10)
Intensity
Zeta Potential (mV)
e
Nano-Foxf1
Phase
RFP
f
Nanoparticle
Days
0 7 14 28
Bleomycin
Harvest
g
Endothelial
Immune
Epithelial
Fibroblast
Day 14
82.6%
5.4%
3.9%
8.7%
Day 28
74.9%
0.1%
0%
4.3%
Normalized to Mode
DyLight 650

**Supplemental Figure S17. Physical properties of PBAE nanoparticles containing DNA plasmid.** **(a)** Atr-FTIR spectrum of the Bisphenol A glycerolate diacrylate (black), 6-amino-1 hexanol (red) and the PBAEs backbone (green) shows the Michael addition between the amine and carbon-carbon double bonds. **(b)** Gel electrophoresis shows the binding of synthesized PBAE nanoparticles with plasmid DNA. Numbers above lanes refer to the mass ratio (w/w) of polymer/ plasmid. Full restriction is observed at a w/w of 1. **(c)** Dynamic light scattering shows the size distribution of the PBAE nanoparticles mixed with DNA at a mass ratio of 1:10. The average hydrodynamic diameter of PBAE nanoparticles is 147 nm. **(d)** The Zeta potential of PBAE nanoparticles mixed with DNA at a mass ratio of 1:10 is 28.3 mV. **(e)** Images of RFP fluorescence (red) show efficient expression of EEV-*Foxf1-RFP* plasmid in cultured cells treated with nanoparticles that were loaded with EEV-*Foxf1-RFP* (Nano-*Foxf1*). Bar = 100µm. **(f)** Schematic diagram of nanoparticle delivery to mice. Nanoparticles were delivered I.V. at the same day as bleomycin treatment. **(g)** Flow cytometry detected DyLight650-positive nanoparticles in lung endothelial cells on day 14 and 28 after nanoparticles administration.

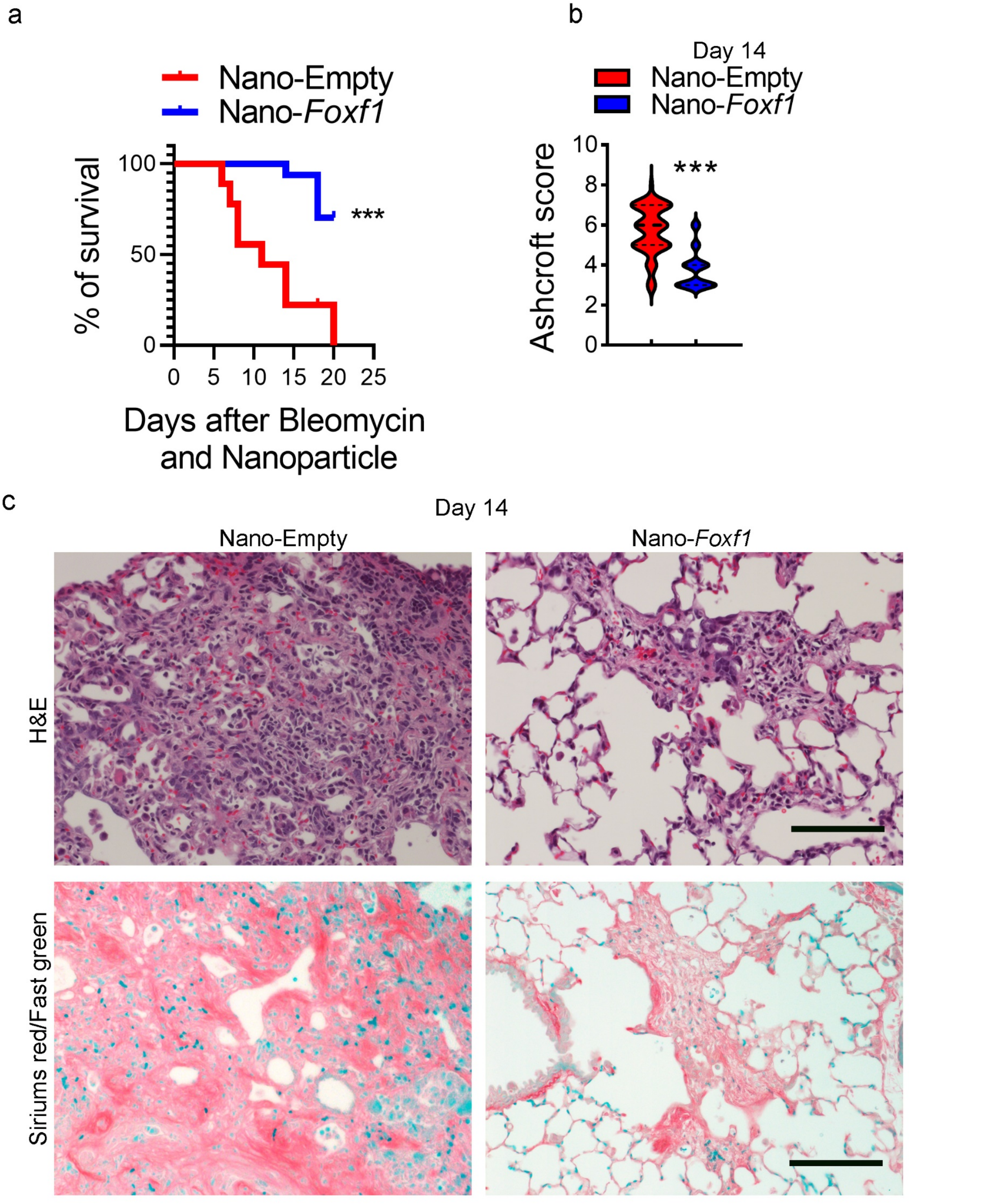
a
Nano-Empty
Nano-*Foxf1*
% of survival
100
50
0
***
0 5 10 15 20 25
Days after Bleomycin and Nanoparticle
b
Day 14
Nano-Empty
Nano-*Foxf1*
Ashcroft score
10
8
6
4
2
0
***
c
Day 14
Nano-Empty
Nano-*Foxf1*
H&E
Siriums red/Fast green

**Supplemental Figure S18. Nanoparticle delivery of non-integrating *Foxf1* expression vector into the lung endothelium at the same day as bleomycin injury attenuates pulmonary fibrosis. (a)** Kaplan-Meier analysis shows increased survival of bleomycin-injured mice after nanoparticle delivery of EEV-*Foxf1* plasmid (Nano-*Foxf1*, n=9) compared to bleomycin-injured mice treated with control EEV-Empty plasmid (Nano-Empty, n=8). Nanoparticles were administered I.V. at the same day as bleomycin treatment I.T. (day 0). ***p = 0.0005. Data were analyzed by Log-rank (Mantel-Cox) test. **(b)** Ashcroft scores were quantified for the fibrotic lungs harvested at day 14 after bleomycin treatment. N=3 mice per group. Data were analyzed by student's t test (two tailed). **(c)** Nano-Foxf1 treatment on day 0 after bleomycin injury decreases lung fibrosis. H&E and Sirius red / fast green staining show decreased lung remodeling in Nano-Foxf1-treated mice compared to mice treated with Nano-Empty (n=3 mice per group). Bar = 100µm. Source data are provided as a Source Data file.

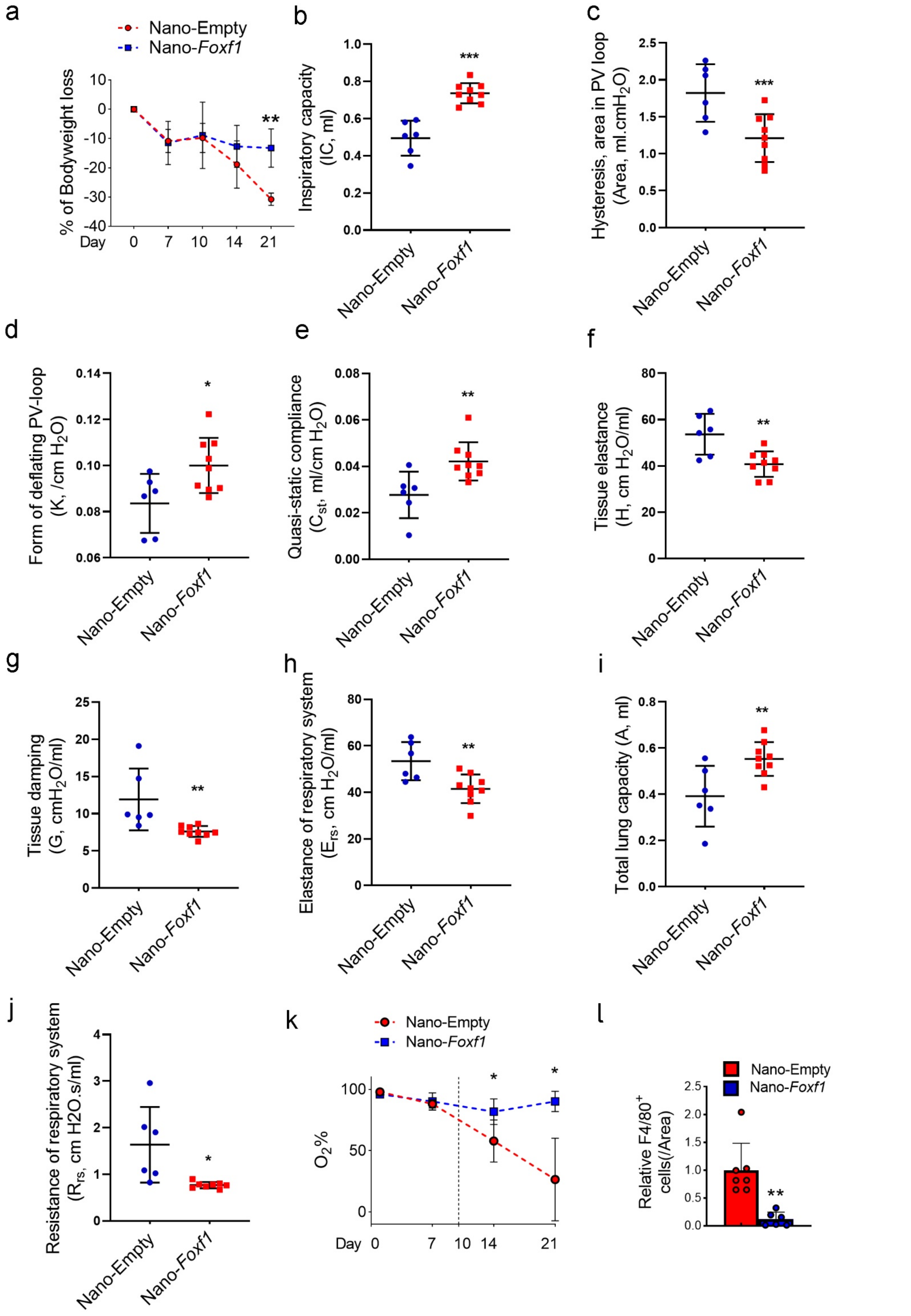
a
Nano-Empty
Nano-Foxf1
% of Bodyweight loss
Day 0 7 10 14 21
**
b
Inspiratory capacity (IC, ml)
***
c
Hysteresis, area in PV loop (Area, ml.cmH2O)
***
d
Form of deflating PV-loop (K, /cm H2O)
*
e
Quasi-static compliance (Cst, ml/cm H2O)
**
f
Tissue elastance (H, cm H2O/ml)
**
g
Tissue damping (G, cmH2O/ml)
**
h
Elastance of respiratory system (Ers, cm H2O/ml)
**
i
Total lung capacity (A, ml)
**
j
Resistance of respiratory system (Rrs, cm H2O.s/ml)
*
k
O2%
Day 0 7 10 14 21
*
*
l
Relative F4/80+ cells(/Area)
**

**Supplemental Figure S19. Nanoparticle delivery of non-integrating *Foxf1* expression vector into the lung endothelium at day 10 after bleomycin injury improves lung functions in mice. (a)** Nanoparticle delivery of Foxf1 decreases bodyweight loss during development of fibrosis. Mice body weight were monitored every week after bleomycin injury. N=10 mice per group. **(b-j)** Nanoparticle delivery of Foxf1 improved lung functions in mice. Lung functions were measured on day 21 after bleomycin administration using FlexiVent system (Nano-Empty, n=6; Nano-*Foxf1*, n=9 biology independent mice). **(k)** Oxygen saturation was improved after nanoparticle treatment on day 21 after bleomycin administration (Day 1 Nano-Empty and Nano-*Foxf1*, n=6; Day 7 Nano-Empty and Nano-*Foxf1*, n=5; Day 14 Nano-Empty, n=6; Nano-*Foxf1*, n=7; Day 21 Nano-Empty, n=5; Nano-*Foxf1*, n=6 biology independent mice). (l) Average number of F4/80-positive cells were quantified using 10 random microscope fields per lung. Data presented as mean ± SD (Nano-Empty, n=7; Nano-*Foxf1*, n=6 biology independent mice). *p<0.05, **p<0.01, ***p<0.001 by student's t test (two tailed). Source data are provided as a Source Data file.

Table S1. List of Primers.

| Mouse Taqman Primers for qRT-PCR | | | |
|---|---|---|---|
| *Actb* | Mm00607939_s1 | *Tnf* | Mm00443258_m1 |
| *Foxf1* | Mm00487497_m1 | *Thbs1* | Mm01335418_m1 |
| *Col3a1* | Mm00802333_m1 | *Acta2* | Mm00725412_s1 |
| *Col1a1* | Mm00801666_g1 | *Ctgf* | Mm01192933_g1 |
| *Rras* | Mm00485862_m1 | *Cthrc1* | Mm01163611_m1 |
| *Ccl2* | Mm00441242_m1 | *Vim* | Mm00449201_m1 |
| *Cxcl1* | Mm04207460_m1 | *Fn1* | Mm01256744_m1 |
| *Il6* | Mm01210733_m1 | | |
| Human Taqman Primers for qRT-PCR | | | |
| *FOXF1* | Hs00230962_m1 | *FN1* | Hs01549976_m1 |
| *ACTB* | Hs99999903_m1 | *COL3A1* | Hs00943809_m1 |
| *ACTA2* | Hs00426835_g1 | *RRAS* | Hs00196699_m1 |
| *VIM* | Hs00958111_m1 | *COL1A1* | HS00164004_m1 |
| Genotyping primers for *TetO7-HA-mFoxf1*$^{tg/+}$ | | | |
| Forward | 5'- TTG GCT GGC AAC GTG GAC G -3' | | |
| Reverse | 5'- TCA CAT CAC ACA CGG CTT GAT G -3' | | |

Table S2. Antibodies used for immunofluorescence

| Antibody | Company | Catalog # | Dilution |
|---|---|---|---|
| FOXF1 | R&D Systems | AF4798 | 1:100 |
| CD31 | Abcam | AB28364 | 1:200 |
| CD31 | R&D Systems | AF3628 | 1:200 |
| aSMA | Sigma Aldrich | a5228 | 1:10,000 |
| RRAS | Cell signaling | 8446s | 1:200 |
| ERG | Abcam | ab92513 | 1:250 |
| Mac3 | BD bioscience | 550292 | 1:100 |
| F4/80 | Biolegend | 122602 | 1:100 |

Table S3. Antibodies used for Flow cytometry.

| Antibody | Conjugate | Company | Catalog # | Dilution |
|---|---|---|---|---|
| CD45 | BV605 | Biolegend | 103140 | 1:100 |
| CD11c | AF700 | eBioscience | 56-0114-82 | 1:100 |
| CD11b | PE-eF610 | eBioscience | 61-0112-82 | 1:100 |
| CD64 | BV421 | Biolegend | 139309 | 1:100 |
| CD24 | APC-eF780 | eBioscience | 47-0242-80 | 1:100 |
| MHC-II | FITC | eBioscience | 11-5321-82 | 1:100 |
| Ly6G | PE-Cy7 | Biolegend | 127618 | 1:100 |
| Ly6C | APC | Biolegend | 128015 | 1:100 |
| Live/Dead | Zombie UV | Biolegend | 423107 | 1:100 |
| CD31 | eF450 | eBioscience | 48-0311-82 | 1:100 |

Table S4. Characteristics of Lung Transplant Donors and Patients with Pulmonary Fibrosis

| Sample | Age | Sex | Smoking | Case of death | Sample ID |
|---|---|---|---|---|---|
| Donor | 50-60 | Male | Former | Intracranial hemorrhage | GSM3489185 |
| Donor | 20-30 | Male | Never | Head trauma from gunshot wound | GSM3489197 |
| Donor | 20-30 | Female | unknown | Seizure | GSM6568653 |
| IPF | 60-70 | Male | unknown | unknown | GSM6568651 |
| IPF | 60-70 | Male | unknown | unknown | GSM6568652 |
| IPF | 60-70 | Male | Yes | unknown | GSM3489183 |
| IPF | 60-70 | Male | No | unknown | GSM3489184 |
| IPF | 60-70 | Male | Yes | unknown | GSM3489188 |
| IPF | 70-80 | Female | No | unknown | GSM3489190 |
| Donor | 60-70 | Female | Never | unknown | GSM3489182 |
| Donor | 50-60 | Male | Former | unknown | GSM3489185 |
| Donor | 20-30 | Female | Never | unknown | GSM3489187 |
| Donor | 50-60 | Female | Never | unknown | GSM3489189 |
| Donor | 40-50 | Female | Never | unknown | GSM3489191 |
| Donor | 20-30 | Female | Active | unknown | GSM3489193 |
| Donor | 40-50 | Female | Never | unknown | GSM3489195 |
| Donor | 20-30 | Female | Active | unknown | GSM3489197 |
