# Supplementary material for: Lung endothelial cells regulate pulmonary fibrosis through FOXF1/R-Ras signaling
Source: Nat Commun. 2023 May 4;14:2560. doi: 10.1038/s41467-023-38177-2 (PMC10156846; doi:10.1038/s41467-023-38177-2)
Supplement: Supplementary file 2 — Reporting Summary [file 41467_2023_38177_MOESM2_ESM.pdf]

nature portfolio

Corresponding author(s): Tanya V Kaalin

Last updated by author(s): 4/6/2023

Reporting Summary

Nature Portfolio wishes to improve the reproducibility of the work that we publish. This form provides structure for consistency and transparency in reporting. For further information on Nature Portfolio policies, see our Editorial Policies and the Editorial Policy Checklist.

Statistics

For all statistical analyses, confirm that the following items are present in the figure legend, table legend, main text, or Methods section.

n/a	Confirmed	
☐	☒	The exact sample size (*n*) for each experimental group/condition, given as a discrete number and unit of measurement
☐	☒	A statement on whether measurements were taken from distinct samples or whether the same sample was measured repeatedly
☐	☒	The statistical test(s) used AND whether they are one- or two-sided<br>*Only common tests should be described solely by name; describe more complex techniques in the Methods section.*
☐	☒	A description of all covariates tested
☐	☒	A description of any assumptions or corrections, such as tests of normality and adjustment for multiple comparisons
☐	☒	A full description of the statistical parameters including central tendency (e.g. means) or other basic estimates (e.g. regression coefficient) AND variation (e.g. standard deviation) or associated estimates of uncertainty (e.g. confidence intervals)
☐	☒	For null hypothesis testing, the test statistic (e.g. *F*, *t*, *r*) with confidence intervals, effect sizes, degrees of freedom and *P* value noted<br>*Give P values as exact values whenever suitable.*
☒	☐	For Bayesian analysis, information on the choice of priors and Markov chain Monte Carlo settings
☒	☐	For hierarchical and complex designs, identification of the appropriate level for tests and full reporting of outcomes
☒	☐	Estimates of effect sizes (e.g. Cohen's *d*, Pearson's *r*), indicating how they were calculated

Our web collection on statistics for biologists contains articles on many of the points above.

Software and code

Policy information about availability of computer code

Data collection	CellRanger pipeline (10X Genomics version 2.1.1) was used to process mouse and human scRNA-seq files into the respective alignment and visualization data.<br>For flow cytometry, we used FACSCanto II or LSR II (BD Biosciences).<br>For FACS sorting, we used FACSAria II (BD Biosciences).<br>Arterial oxygenation was measured using MouseOx+pulse oximeter (STARR Life Sciences).<br>Nikon's Eclipse 90i digital microscope and Nikon's A1R HD25 confocal microscope were used for data collection.

Data analysis	We used GraphPad Prism vs 9 (GraphPad Software, Inc., San Diego, CA) to perform the statistical analysis.<br>Nikon's NIS-Elements AR (Advanced Research, ver. 5) software.<br>FlowJo Software version 10.8.0<br>R (version 3.6.1) using custom scripts, SINCERA, and Seurat (version 3)<br>FlexiWare software (version 7.5, Service Pack 4).<br>BioWardrobe' platform. No version number available.<br>ToppGene Suite (an online based tool, no version number)<br>MouseOxPlus Revision 1.6.X software<br>FACSDiva 9.0.

For manuscripts utilizing custom algorithms or software that are central to the research but not yet described in published literature, software must be made available to editors and reviewers. We strongly encourage code deposition in a community repository (e.g. GitHub). See the Nature Portfolio guidelines for submitting code & software for further information.

Data

Policy information about availability of data

All manuscripts must include a data availability statement. This statement should provide the following information, where applicable:

- Accession codes, unique identifiers, or web links for publicly available datasets
- A description of any restrictions on data availability
- For clinical datasets or third party data, please ensure that the statement adheres to our policy

All the raw sequence data generated in this study is deposited in GEO database (accession number GSE213018; https://www.ncbi.nlm.nih.gov/geo/query/acc.cgi?acc=GSE213018). Single cell RNA sequencing data generated in this study from donor and IPF patient lungs (Supplemental Figure S4) were deposited in GEO database (accession number GSE213017). Single cell RNA sequencing data generated in this study from bleomycin-treated and untreated adult mice lung were deposited in GEO database (accession number GSE213016). Single cell RNA sequencing data of Northwestern University (NW) datasets from donor and IPF lungs were retrieved from GEO database (GSE122960; https://www.ncbi.nlm.nih.gov/geo/). Bulk RNA sequencing data generated in this study from bleomycin-treated control and endFoxf1+/- mouse lung endothelial cells were also deposited in GEO database (GSM6578251 and GSM6578252). Source data are provided with this paper.

Human research participants

Policy information about studies involving human research participants and Sex and Gender in Research.

Reporting on sex and gender	Both sexes were used. We have not noticed any sex-dependent differences.
Population characteristics	NA
Recruitment	NA
Ethics oversight	The Institutional Review Board of the Cincinnati Children's Hospital Medical Center (Federalwide Assurance #00002988) approved all the studies with human tissue samples (IRB protocol #2017-4321). Human lung tissue specimens were obtained from tissue repository at University of Cincinnati Medical Center that provides de-identified human biospecimen procurement and banking services in support of basic, translational, and clinical research. Total number of ten lung tissue samples were received, including six males and four females. All patients signed informed consent prior to tissues collection. Explanted lungs were acquired from donors with end-stage IPF lung disease undergoing transplant or from rejected control donor lungs.

Note that full information on the approval of the study protocol must also be provided in the manuscript.

Field-specific reporting

Please select the one below that is the best fit for your research. If you are not sure, read the appropriate sections before making your selection.

☒ Life sciences ☐ Behavioural & social sciences ☐ Ecological, evolutionary & environmental sciences

For a reference copy of the document with all sections, see nature.com/documents/nr-reporting-summary-flat.pdf

Life sciences study design

All studies must disclose on these points even when the disclosure is negative.

Sample size	Power analysis (DSTPLAN 4.2 software) with the power value of 0.9 predicted that the sample size for the lung fibrosis studies should be approximately 5 mice per group per time point.
Data exclusions	No data were excluded from the experiments.

Replication	All in vitro experiments were performed using three biological and technical replications. All in vivo and in vitro experiments in this study have been repeated independently with similar results more than 3 times.
Randomization	For the in vivo studies, no formal randomization method was used as the genotype of the mice was known before starting the experiment. Mice were randomly distributed in different cages for the entire duration of the experiment. For in vitro studies, samples were randomly allocated into experimental groups.
Blinding	Blinding was implemented for the scoring of the lung sections to assess severity of fibrotic remodeling. Other analysis was done without blinding.

Reporting for specific materials, systems and methods

We require information from authors about some types of materials, experimental systems and methods used in many studies. Here, indicate whether each material, system or method listed is relevant to your study. If you are not sure if a list item applies to your research, read the appropriate section before selecting a response.

Materials & experimental systems

n/a	Involved in the study
☐	☒ Antibodies
☐	☒ Eukaryotic cell lines
☒	☐ Palaeontology and archaeology
☐	☒ Animals and other organisms
☒	☐ Clinical data
☒	☐ Dual use research of concern

Methods

n/a	Involved in the study
☒	☐ ChIP-seq
☐	☒ Flow cytometry
☒	☐ MRI-based neuroimaging

Antibodies

Antibodies used

Flow Cytometry
anti-CD45:BV605 (103140, clone 30-F11, Biolegend, San Diego, CA, USA, 1:100 Dilution), anti-CD11c:AF700 (56-0114-82, clone N418 ,eBioscience, San Diego, CA, USA, 1:100 Dilution), anti-Cd11b:PE-eF610 (61-0112-82, clone M1/70 ,eBioscience, San Diego, CA, USA, 1:100 Dilution), anti-CD64:BV421 (139309, clone X54-5/7.1, Biolegend, San Diego, CA, USA, 1:100 Dilution), anti-CD24:APC-eF780 (47-0242-80, clone M1/69, eBioscience, San Diego, CA, USA, 1:100 Dilution), anti-MHC-II:FITC (11-5321-82, clone M5/114.15.2, eBioscience, San Diego, CA, USA, 1:100 Dilution), anti-Ly6G:PE-Cy7 (127618, clone 1A8, Biolegend, San Diego, CA, USA, 1:100 Dilution), anti-Ly6C:APC (128015, clone HK1.4, Biolegend, San Diego, CA, USA, 1:100 Dilution), Zombie UV (423107, Biolegend, San Diego, CA, USA, 1:1000 Dilution), anti-CD31:eF450 (48-0311-82, clone 390, eBioscience, San Diego, CA, USA, 1:100 Dilution).

Immunofluorescence staining
Anti-FOXF1 (AF4798, R&D Systems, Minneapolis, MN, USA, 1:100 Dilution), anti-CD31 (AB28364, Abcam, Cambridge, MA, USA, 1:200 Dilution), anti-CD31 (AF3628, R&D Systems, Minneapolis, MN, USA, 1:200 Dilution), anti-αSMA (A5228, clone 1A4, Sigma Aldrich, St. Louis, MO, USA, 1:10,000 Dilution), anti-RRAS (8446s, Cell signaling Technology, Danvers, MA, USA, 1:200 Dilution), anti-ERG (AB92513, Abcam, Cambridge, MA, USA, 1:250 Dilution), anti-Mac3 (550292, clone M3/84, BD bioscience, Franklin Lakes, NJ, USA, 1:100 Dilution), Anti-F4/80(122602, clone CI-A3-1, Biolegend, San Diego, CA, USA, 1:100 Dilution).

Validation

All antibodies were commercially available and were validated by the respective manufactures. Additional information can be found at the following links:
anti-CD45 (https://www.biolegend.com/fr-lu/products/brilliant-violet-605-anti-mouse-cd45-antibody-8721)
anti-CD11c (https://www.thermofisher.com/antibody/product/CD11c-Antibody-clone-N418-Monoclonal/56-0114-82)
anti-Cd11b (https://www.thermofisher.com/antibody/product/CD11b-Antibody-clone-M1-70-Monoclonal/61-0112-82)
anti-CD64 (https://www.biolegend.com/en-us/products/brilliant-violet-421-anti-mouse-cd64-fcgammari-antibody-8992)
anti-CD24 (https://www.thermofisher.com/antibody/product/CD24-Antibody-clone-M1-69-Monoclonal/47-0242-82)
anti-MHC-II (https://www.thermofisher.com/antibody/product/MHC-Class-II-I-A-I-E-Antibody-clone-M5-114-15-2-Monoclonal/11-5321-82)
anti-Ly6G (https://www.biolegend.com/en-us/search-results/pe-cyanine7-anti-mouse-ly-6g-antibody-6139?GroupID=BLG7234)
anti-Ly6C (https://www.biolegend.com/fr-lu/products/apc-anti-mouse-ly-6c-antibody-6047)
Zombie UV (https://www.biolegend.com/en-ie/products/zombie-uv-fixable-viability-kit-9336)
anti-CD31 (https://www.thermofisher.com/antibody/product/CD31-PECAM-1-Antibody-clone-390-Monoclonal/48-0311-82)
anti-FOXF1 (https://www.rndsystems.com/products/human-mouse-foxf1-antibody_af4798)
anti-CD31 (https://www.abcam.com/cd31-antibody-ab28364.html)
anti-CD31 (https://www.rndsystems.com/products/mouse-rat-cd31-pecam-1-antibody_af3628)
anti-αSMA (https://www.sigmaaldrich.com/US/en/product/sigma/a5228)
anti-RRAS (https://www.cellsignal.com/products/primary-antibodies/r-ras-antibody/8446)
anti-ERG (https://www.abcam.com/erg-antibody-epr3864-ab92513.html?productWallTab=ShowAll)
anti-Mac3 (https://www.bdbiosciences.com/en-us/products/reagents/flow-cytometry-reagents/research-reagents/single-color-antibodies-ruo/purified-rat-anti-mouse-cd107b.550292)

Eukaryotic cell lines

Policy information about cell lines and Sex and Gender in Research

Cell line source(s)	Cell lines used within the study were obtained from Lonza, ATCC, and Seven Hills:<br>Human Umbilical Vein Endothelial Cells (HUVEC, Lonza, # C2519A)<br>Human Pulmonary Microvascular Endothelial Cells (HPMEC, Lonza, # CC-2527)<br>Human Pulmonary Artery Endothelial Cells (HPAEC, Lonza, # CC-2530)<br>Human Peripheral Blood Macrophages (Lonza, # 4W-700)<br>Human CCD-19Lu fibroblast cell line (ATCC, # CCL-210)<br>Mouse endothelial MFLM-91U cells (Seven Hills, # AMFLM-91U)
Authentication	Cell lines used were not authenticated.
Mycoplasma contamination	There were no Mycoplasma contamination.
Commonly misidentified lines<br>(See ICLAC register)	No commonly misidentified lines were used.

Animals and other research organisms

Policy information about studies involving animals; ARRIVE guidelines recommended for reporting animal research, and Sex and Gender in Research

Laboratory animals	Generation of endothelial-cell specific Foxf1 heterozygous mice (mixed sex): Foxf1fl/fl mice17 were crossed with Pdgfb-iCreERtg/- mice to generate Pdgfb-iCreER/Foxf1fl/+ (abbreviated as endFoxf1+/-) mice in C57BL6 genetic background.<br>Generation of TetO7-HA-mFoxf1tg/+ mice: The 5'- HA-tagged coding region of mouse Foxf1 gene in a Shuttle vector was subcloned into a TetO7-CMV vector. The 2 kb DNA fragment containing TetO7-CMV-HA-Foxf1 was microinjected into the pronucleus of fertilized mouse eggs.<br>Generation of endothelial-cell specific FOXF1 overexpression mice: TetO7-HA-mFoxf1tg/+ mice were crossed with Pdgfb-iCreERtg/- mice and Rosa26-LSL-rtTAtg/+ 57 to generate Pdgfb-iCreER/Rosa26-LSL-rtTA/TetO7-HA-mFoxf1 mice in C57BL6/129sv/FVB hybrid genetic background.<br>Mice were housed in the animal care facility at Cincinnati Children's Hospital Medical Center. 8-9 weeks old mice were used in experiments. All mice were kept under SPF (specific-pathogen free) conditions in 12/12 light/dark cycle, 18-23oC and 40-60% humidity. Both male and females were used for studies.
Wild animals	No wild animals were used.
Reporting on sex	Animal we used in the study were mixed sex.
Field-collected samples	No field-collected samples were used.
Ethics oversight	All animal studies were approved by Cincinnati Children's Research Foundation Institutional Animal Care and Use Committee and covered under our animal protocol (IACUC2016-0070). The Cincinnati Children's Research Foundation Institutional Animal Care and Use Committee is an AAALAC and NIH accredited institution (NIH Insurance #8310801).

Note that full information on the approval of the study protocol must also be provided in the manuscript.

Flow Cytometry

Plots

Confirm that:

- [x] The axis labels state the marker and fluorochrome used (e.g. CD4-FITC).
- [x] The axis scales are clearly visible. Include numbers along axes only for bottom left plot of group (a 'group' is an analysis of identical markers).
- [x] All plots are contour plots with outliers or pseudocolor plots.
- [x] A numerical value for number of cells or percentage (with statistics) is provided.

Methodology

Sample preparation	To generate a single cell suspension, fresh lung tissue was minced and incubated in an enzymatic cocktail containing 60 ml dispase (5U/ml, Stemcell Technologies), 900 µl liberase (1g/l, Sigma Aldrich), 0.03 g DNase (Sigma Aldrich) at 37-degree Celsius for 45 minutes. Digestion was inactivated with sorting buffer (2% FBS, 0.5 mM EDTA pH7.4 in PBS), the cell suspension was passed through 70 µm cell strainer to remove debris. Cell were then centrifuged (1,300 rpm, 5 min, 4-degree Celsius), and resuspended in 2 ml ACK lysis buffer for 1 minute to remove the remaining red blood cells and diluted in 4 ml sorting buffer after incubation. Cells were then centrifuged (1,300 rpm, 5 min, 4-degree Celsius) and resuspended in 200 µl of sorting buffer.

Instrument	Stained cells were analyzed using FACSCanto II or LSR II (BD Biosciences). and were sorted using cell sorting (five-laser FACSAria II; BD Biosciences)
Software	Data were analyzed in BD FACSDiva and FlowJo 10.8.0
Cell population abundance	Sufficient number of cells was used in each (100,000 to 500,000 cells per experiments).
Gating strategy	Gates were placed around cell populations with common characteristics after determining forward scatter, side scatter, single staining, and fluorescence minus one strategy.

☒ Tick this box to confirm that a figure exemplifying the gating strategy is provided in the Supplementary Information.
